# Development of a TSR-Based Method for Protein 3-D Structural Comparison With Its Applications to Protein Classification and Motif Discovery

**DOI:** 10.3389/fchem.2020.602291

**Published:** 2021-01-13

**Authors:** Sarika Kondra, Titli Sarkar, Vijay Raghavan, Wu Xu

**Affiliations:** ^1^The Center for Advanced Computer Studies, University of Louisiana at Lafayette, Lafayette, LA, United States; ^2^Department of Chemistry, University of Louisiana at Lafayette, Lafayette, LA, United States

**Keywords:** protein structure comparison, triangular spatial relationship, structure motifs, protein classification, protein structure and function relation, protein secondary structure, molecular dynamics simulation, protein conformational change

## Abstract

Development of protein 3-D structural comparison methods is important in understanding protein functions. At the same time, developing such a method is very challenging. In the last 40 years, ever since the development of the first automated structural method, ~200 papers were published using different representations of structures. The existing methods can be divided into five categories: sequence-, distance-, secondary structure-, geometry-based, and network-based structural comparisons. Each has its uniqueness, but also limitations. We have developed a novel method where the 3-D structure of a protein is modeled using the concept of Triangular Spatial Relationship (TSR), where triangles are constructed with the C_α_ atoms of a protein as vertices. Every triangle is represented using an integer, which we denote as “key,” A key is computed using the length, angle, and vertex labels based on a rule-based formula, which ensures assignment of the same key to identical TSRs across proteins. A structure is thereby represented by a vector of integers. Our method is able to accurately quantify similarity of structure or substructure by matching numbers of identical keys between two proteins. The uniqueness of our method includes: (i) a unique way to represent structures to avoid performing structural superimposition; (ii) use of triangles to represent substructures as it is the simplest primitive to capture shape; (iii) complex structure comparison is achieved by matching integers corresponding to multiple TSRs. Every substructure of one protein is compared to every other substructure in a different protein. The method is used in the studies of proteases and kinases because they play essential roles in cell signaling, and a majority of these constitute drug targets. The new motifs or substructures we identified specifically for proteases and kinases provide a deeper insight into their structural relations. Furthermore, the method provides a unique way to study protein conformational changes. In addition, the results from CATH and SCOP data sets clearly demonstrate that our method can distinguish alpha helices from beta pleated sheets and *vice versa*. Our method has the potential to be developed into a powerful tool for efficient structure-BLAST search and comparison, just as BLAST is for sequence search and alignment.

## Introduction

Availability of protein sequences and structures has been rapidly increasing. Both play fundamental roles in understanding protein functions. It is well-accepted that protein structures are more conserved than sequences. Understanding the 3-D structure, rather than pure 1-D relationships, provides deeper insights into protein functions. To accelerate discovery in all areas of biological and chemical sciences, efforts have been made in three directions: (1) constructing 3-D structure database, e.g., PDB (Berman et al., 2000) and structure-based protein classification database, e.g., CATH (Greene et al., 2007), FSSP (Holm and Sander, 1996), SCOP (Murzin et al., 1995); (2) developing computational methods, e.g., MD simulations, QM/MM calculations, beyond the resources of experimental data, for generating and optimizing theoretical structures; (3) developing algorithms for 3-D structural comparison or alignment. Efforts have also been made in combining structure comparison algorithms with sequences analysis to achieve better understanding of sequence, structure, and function relationships.

There is a considerable amount of ambiguity in how the existing approaches describe the 3-D relationships between proteins. The general idea of structural alignment can be considered to start from 1960 when myoglobin and hemoglobin structures were compared (Perutz et al., 1960). Systematic structural alignment began with the analysis of heme binding proteins and dehydrogenases by Rossmann et al. (Rossmann and Argos, 1975). The first structural comparison program on the basis of automation was developed in 1980 (Remington and Matthews, 1980). Challenges in quantifying structural similarity led to the large number of methods to address this problem described in the literature over the last 40 years. The common schema of those methods can be summarized into three steps. The first step is to extract features from the atomic coordinates of the 3-D structures. In other words, coordinates can be used for representations by distance, topology, and/or geometry. Based on how the structural features are extracted, the existing protein structural comparison/alignment methods can be divided into five categories: sequence-based, distance-based, secondary structure-based, geometry-based, and network-based at either local or global structure levels. Some of the methods belong strictly to one category, while others use a combination of the methods from two or more categories. The second step is to convert those features into scores using specific algorithms for quantifying similarity between two structures. The last step is to employ statistical analysis, e.g., Z-score and *p*-value, to provide confidence for the structural similarity. A number of algorithms have been developed and/or employed for structural comparisons: Maximal common subgraph detection (Bron and Kerbosch, 1973), Ullmann subgraph isomorphism algorithm (Ullmann, 1976), and geometric hashing (Nussinov and Wolfson, 1991) in geometry-based; Monte Carlo (Holm and Sander, 1993), Combinatorial Extension (CE) (Shindyalov and Bourne, 1998), and Comparative Structural Alignment (CSA) (Wohlers et al., 2012) algorithms in distance-based, a genetic algorithm (Szustakowski and Weng, 2000) and Dictionary of Secondary Structure of Proteins (DSSP) (Kabsch and Sander, 1983) in secondary structure-based comparisons, and amino acid network (AAN) (Alves and Martinez, 2007; Bartoli et al., 2008) including C_α_ network (CAN) and atom distance network (ADN) and interaction selective network (ISN) (Konno et al., 2019) in network-based comparisons. Dynamic programming algorithms have been used in both distance- (Blundell et al., 1988; Taylor and Orengo, 1989; Lackner et al., 2000) and secondary structure-based (Taylor and Orengo, 1989; Yang and Honig, 2000) comparisons.

Results generated from the existing methods vary considerably (Kolodny et al., 2005; Mayr et al., 2007). Studies indicated that different methods should be combined for gaining meaningful structural relationships (Kolodny et al., 2005; Wohlers et al., 2012). Many established comparison techniques evaluate structural similarity by maximizing the number of equivalent residues through either sequence or structural alignment, and minimizing global differences in distance. A major challenge with such an approach is in the identification of equivalent residues. Earlier methods for homologous proteins used sequence string matching techniques to aid in finding the initial structural equivalence (Bron and Kerbosch, 1973). Nonetheless, the alignment tends to be error prone when the sequence similarity is low. Thus, newer approaches are mostly structure-based and they derive the initial equivalences by detecting similarities in the local structural regions, e.g., CE (Shindyalov and Bourne, 1998) or by using secondary structures, e.g., SSM (Krissinel and Henrick, 2004), Vector Alignment Search Tool (VAST) (Madej et al., 1995), or both, e.g., LOCK (Singh and Brutlag, 1997), LOCK 2 (Shapiro and Brutlag, 2004a). It is difficult to simultaneously optimize the number of equivalent residues and the global differences in distance since one may have to be optimized at the expense of the other (Zemla, 2003). An additional challenge can arise when structures are similar in small local regions. These regions of similarity can be overlooked when a single global superposition is applied (Zemla, 2003).

Protein structural alignment, unlike the counter part for protein sequences, has not yet enjoyed a widely accepted comparison or search method. Depending on how they represent structures and how they handle challenges, different methods have their strengths and weaknesses. There has not been a method to obtain the “best” structural comparison. While there are limitations in the existing methods, the limitations also leave much room for exciting new research work to be done. TSR (Triangular Spatial Relationship) was originally designed for 2-D symbolic images (Guru and Nagabhushan, 2001). We have extending the concept of TSR to a new method, designated as TSR-based 3-D structural comparison where the (x, y, z) coordinates associated with C_α_ atoms of amino acids are first converted to all possible triangles represented by vertices, edge lengths and angles, and then the vertices, edge lengths, and angles of each triangle are combined to be represented by an integer through a set of rule-based formulae. We refer to the integer representing a triangle as a key. As a result, the 3-D structure of a protein is represented by a vector of keys. Our approach has several unique features: (i) A unique way to represent structures to avoid performing structural superimposition. It avoids structural rotation and translation, and most importantly, it overcomes the need to compromise between maximizing number of equivalent residues and minimizing the RMSD; (ii) Inter-chain residue distances (intra-chain residue distances) are based on the distance between two C_α_ atoms (respectively, pairs of atoms within a protein). Since a distance computation does not capture the underlying shape information and the respective amino acids are not included in the distance matrix calculations, motif discovery cannot be easily accomplished by searching for similar distance values. In contrast, triangles are probably the simplest primitives to capture the shape. We have included amino acid information in the formula of the key calculations to avoid assigning two triangles with only similar geometries the same key if any of three amino acids is different between those two triangles. Our method allows an effective and accurate identification of similar local structures even when two structures are different at a global level. In addition, our approach can establish whether two or more triangles are connected by a vertex or an edge, thus enabling discovery of more complex shared substructures. Only a few programs [e.g., ASSAM/SPRITE (Nadzirin et al., 2012), IMAAAGINE (Nadzirin et al., 2013), Recursive Automatic Search of MOTif in 3D structures of PROteins (RASMOT-3D PRO) (Debret et al., 2009), SPASM (Kleywegt, 1999), and MSDmotif (Golovin and Henrick, 2008)] were developed with the specific goal of protein motif search. Such motif search is limited to about 12 residues. Triangles were used for alignment-based motif discovery (Nussinov and Wolfson, 1991), identification of similar surface geometries and electrostatic potentials (Kinoshita and Nakamura, 2003) and description of enzyme active sites (Dodson and Wlodawer, 1998). Libraries of multiple fragments, through breaking protein structures down to their constituent parts, have been developed for a precise and complete description of protein backbone conformation (Vetrivel et al., 2017). They differ in the number of fragments, the length of the fragments, the methods used for clustering and the criteria used for clustering. Structural alphabets (SAs) is a library of N structural prototypes (the letters). Each prototype is representative of a backbone local structure of l-residues length. Depending on the targeted accuracy, the length l and the number N can vary significantly. The length l typically ranges between 4 and 9 and the most frequent value of N is close to 20 (Offmann et al., 2007). The first fragment library was developed by Unger et al. (Unger et al., 1989), and subsequent different fragment libraries can found in (Karchin et al., 2004; Offmann et al., 2007). In (Pandini et al., 2010) a simple and explicit description of four-residue long fragments, where the conformation of each was defined by three internal angles, was devised. One of the most developed and comprehensive SA is the Protein Blocks (PBs) approach (de Brevern et al., 2000). This SA is composed by 16 local structure prototypes each representing backbone conformation of a fragment of five contiguous residues (de Brevern et al., 2000; Joseph et al., 2010). It was shown to efficiently approximate every part of the protein structure; (iii) Our method enables the computation of similarity values as function of the numbers of identical and different keys representing two proteins, to show structure relationships. Other methods use RMSD or Z-score. Inter-chain distance methods, e.g., STAMP (Russell and Barton, 1992) that uses the procedure developed by Rossmann and Argos (Rossmann and Argos, 1976), require superimposition for calculating RMSD for structure evaluations. Intra-chain distance methods, e.g., DALI (Holm and Sander, 1993) where superimposition is not needed, generate 2-D distance matrices. Z-scores are calculated by comparing distance matrices and the calculated scores are used for ranking structures. Our approach enables the identification of common keys present in all proteins of a given class, and specific keys belonging to only a given class, providing a deeper insight into (sub)structural relationships. A solely structure-based hierarchical organization can be constructed for both homologous and non-homologous proteins and the specific keys identified can be used to distinguish one subclass from others.

This study can be roughly divided into two major parts: TSR-based vectorization of protein 3-D structures, and application/evaluation. Vectorization includes three main steps: development of key generation formula, and determination and evaluation of optimum parameters for key generation formula (Supplementary Figure 1). The details of the parameters are discussed in the Sections of Methods and Results. Application or evaluation comprises five modules. They are protein clustering, motif identification and discoveries, evaluation of our method in studying protein dynamics, differentiation of secondary structure, and comparison of our method with other popular methods (Supplementary Figure 1). A number of protein structure data sets were prepared to achieve the specific goals of each type of application/evaluation (Supplementary Figure 1). Visualization tools were employed in both vectorization and application/evaluation (Supplementary Figure 1). In our future work, a tool will be developed to achieve protein structure-BLAST search analogous to sequence alignment and sequence-BLAST search.

## Methods

### Key Generation

**Figure 1:**
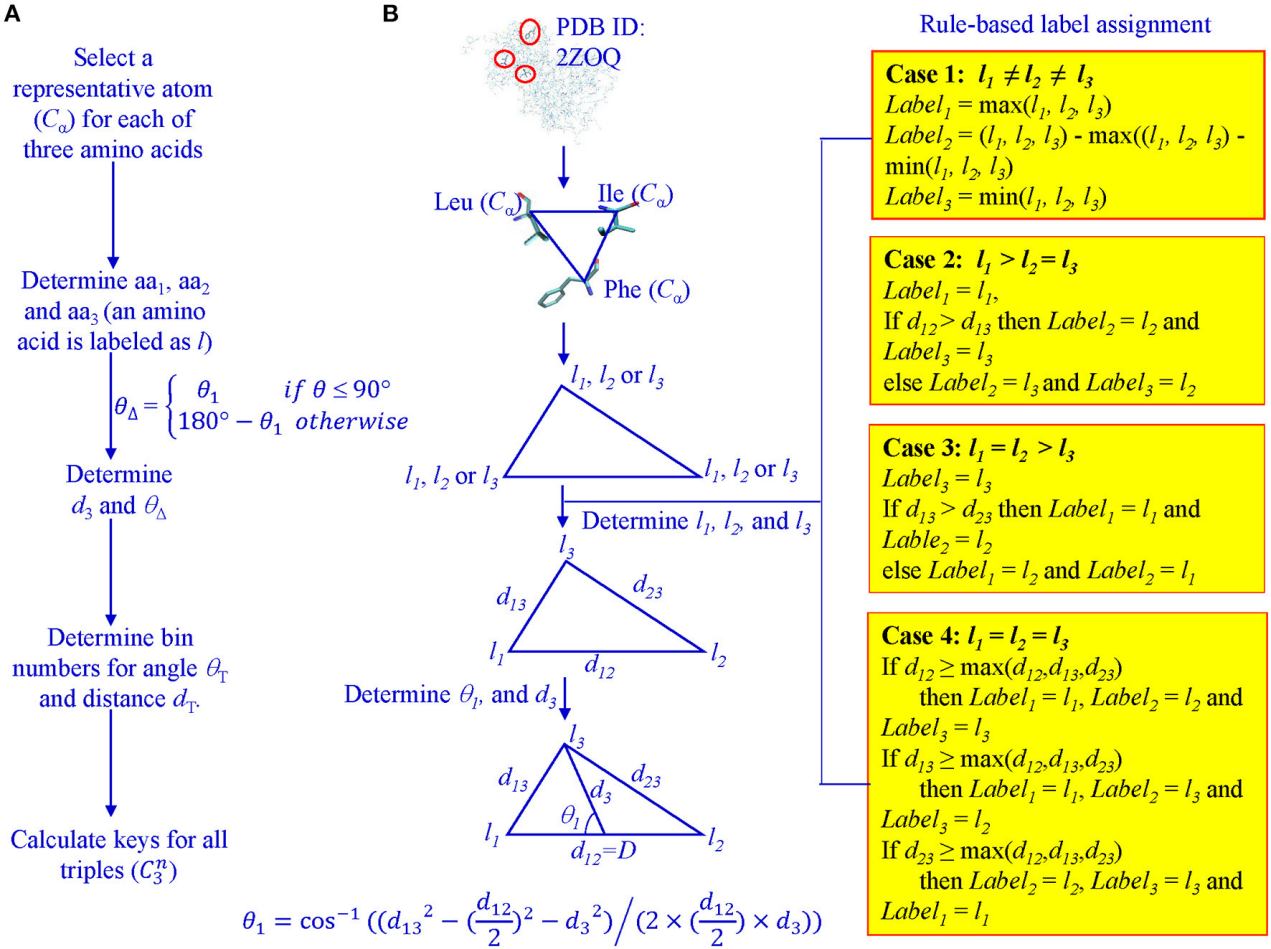
Determination of θ1, D, *l*1, *l*2, and *l*3 for key calculation. **(A)** The workflow from Cα atoms to key calculations; **(B)** A graphic presentation of the rule-based label determination, and definition of θ1.

For every protein, C_α_ atoms from its PDB file were selected. All three lengths and angles of all possible triangles formed by C_α_ were calculated. Each C_α_ of the 20 amino acids was assigned a unique integer identifier in the range (4, 5, …, 23). We transform the integer IDs to *l*_*i*1_, *l*_*i*2_, and *l*_*i*3_ for vertices of triangle *i* based on the rule-based label-determination (Guru and Nagabhushan, 2001). This transformation ensures that corresponding triangles occurring in two different proteins receive the same integer IDs. Once *l*_*i*1_, *l*_*i*2_, and *l*_*i*3_ are determined for triangle *i*, we calculate θ_1_ using Equation 1 and θ_Δ_ based on θ_1_ values (Figure 1).

(1) $$\begin{array}{l} \theta_{1}=\cos^{-1}\left(\left({d_{13}}^{2}-{\left(\frac{d_{12}}{2}\right)}^{2}-{d_{3}}^{2}\right)/\left(2\times \left(\frac{d_{12}}{2}\right)\times d_{3}\right)\right) \end{array}$$

$$\theta_{\Delta }=\left\{ \begin{array}{l} \theta_{1}\;if\;\theta \leq 90^{{}^{\circ}}\\ 180^{{}^{\circ}}-\theta_{1}\;otherwise \end{array}\right.$$

Where

*d*_13_: distance between *l*_*i*1_ and *l*_*i*3_ for triangle *i*
*d*_12_: distance between *l*_*i*1_ and *l*_*i*2_ for triangle *i*
*d*_3_: distance between *l*_*i*3_ and midpoint of *l*_*i*1_ and *l*_*i*2_, for triangle *i*

Once labels: *l*_*i*1_, *l*_*i*2_, *l*_*i*3_, *D*, and θ_Δ_ are determined, we use Equation 2 to calculate the key for each triangle.

(2) $$\begin{array}{l} k=\theta_{T}d_{T}\left(l_{i1}-1\right)m^{2}+\theta_{T}d_{T}\left(l_{i2}-1\right)m+\theta_{T}d_{T}\left(l_{i3}-1\right)\\ \;+\theta_{T}\left(d-1\right)+\left(\theta -1\right) \end{array}$$

where

m: the total number of distinct labels
θ: the bin value for the class in which, the angle representative, falls to achieve discretization we use the Adaptive Unsupervised Iterative Discretization algorithm:
θ_*T*_: the total number of distinct discretization levels (or number of bins) for angle representative
*d*: the bin value for the class in which *D*, the length representative, falls; to achieve discretization we use the Adaptive Unsupervised Iterative Discretization algorithm
*d*_*T*_: the total number of distinct discretization levels (or number of bins) for length representative

The determination of bin boundary values and numbers of bins will be discussed in the Results section. We refer to the value of θ_Δ_ as Theta and *D* as MaxDist. Theta is calculated based on θ_1_ and it is defined as the angle that is <90° between the line from the midpoint of the longest edge to the vertex which other two edges intersect and half of the longest edge (Figure 1). MaxDist is defined as the distance of the longest edge of a triangle (Figure 1). In summary, the key value assigned to a triangle is a function of five parameters: *l*_*i*1_, *l*_*i*2_, *l*_*i*3_, Theta, and MaxDist (Supplementary Figure 1). In the context of protein structures, the use of MaxDist is a scale factor, since, without MaxDist, two triangles of the same shape, but of different size (similar triangles), could not be distinguished; that is, they will be assigned the same key value.

The key calculation is performed by a function that is one-to-one and onto. The function's inputs are treated as digits in a number system. Integers associated with Theta intervals form the lowest order digit, followed by integers associated with MaxDist intervals. Then the integers associated with amino acids are given the next three positions by giving a special rule-based ordering of the integers denoting the three amino acids of a triangle. This guarantees that if only the lowest order input differs by one, the function output also differs by one. If only second order input differs by one, then output differs by θ_*T*_. By the same argument, variations by one in 3rd, 4th and highest order positions will cause output to differ, respectively, by the product of θ_*T*_ and *d*_*T*_, the product of θ_*T*_, *d*_*T*_, and *m*, and the product of θ_*T*_, *d*_*T*_, and *m*^2^. This ensures that changes to any of the digits in the input will change the output (i.e., one-to-one). The onto property can be guaranteed by understanding that given any output value, each of the function inputs can be uniquely recovered. For example, the output value modulo θ_*T*_ gives lowest order input. Taking the previous quotient value's modulo with the product of θ_*T*_ and *d*_*T*_ intervals gives the second order digit of the input and so on. For any output, digits of the input are unique (i.e., onto). If there are q amino acids in the protein data set, the time complexity of our method in the worst case is O(nq^3^) where n is the number of proteins in a given data set.

### Protein Structure Similarity and Distance Calculation

We apply the Generalized Jaccard coefficient measure (Jaccard, 1901), Equation 3, for the calculation of similarity between two proteins (Supplementary Figure 1).

(3) $$\begin{array}{l} \text{Ja}{\text{c}}_{gen}=\overset{n}{\underset{i=1}{\sum }}\epsilon_{i}/\overset{n}{\underset{i=1}{\sum }}z_{i} \end{array}$$

where *n* is the total number of unique keys in proteins *p*_1_and *p*_2_

Equivalence ϵ for a given key *k*_*i*_ in two different proteins *p*_1_ and *p*_2_ is defined as $\epsilon_{i}=k_{i}^{p_{1}}\cap k_{i}^{p_{2}}$ where *n* is defined by the minimum count of the corresponding keys.

Difference *z* for a given key *k*_*i*_ in a pair of proteins is defined as $z_{i}=k_{i}^{p_{1}}\cup k_{i}^{p_{2}}$

where ∪ is defined by the maximum count of the corresponding keys. The count of a key is the number of times that key occurs (occurrence frequency) within a protein.

We also use a variant of the Generalized Jaccard coefficient measure, which we refer to as the modified Generalized Jaccard coefficient measure, Equation 4, to calculate similarity.

(4) $$\begin{array}{l} m\text{Ja}{\text{c}}_{gen}=\overset{n}{\underset{i=1}{\sum }}\epsilon_{i}/min\left(\overset{n}{\underset{i=1}{\sum }}z_{i},max\left(N_{p1},N_{p2}\right)\right) \end{array}$$

Where *N*_*p*1_is a total number of key in *p*_1_

*N*_*p*2_ is a total number of key in *p*_2_

If we do pairwise structural comparison, then the worst case run time complexity is O(n^2^m^3^) where n is the number of proteins in a data set and m is the number of amino acids. Once a similarity matrix is generated, the distance matrix is generated simply by taking each value in the similarity matrix and subtracting it from 1.

### Preparation of Protein Structure Data Sets

We have used seven data sets of varying sizes, from a range of 10–20 structures to ~4,500 structures, in this study. The PDB IDs, chain information and functional classification of these seven data sets are provided as Supplementary Files 1–7, for proteases, theoretical structures (The details are in the following section of molecular dynamics simulation), CDK2, CATH, SCOP, DD (DD data set was selected based on literature), and a small data set with 101 protein structures, respectively (Supplementary Figure 1). The data sets are prepared based on the specific questions we want to address or specific hypotheses we want to test. The main purposes of the protease data set are to test our method on protein clustering and structural motif identification and discovery (Supplementary Figure 1). CDK2 and the theoretical structures are prepared to study small conformational changes upon binding of a ligand or an interacting protein or due to post-translational modifications or mutations (Supplementary Figure 1). CATH, SCOP and DD data sets are used for evaluating our method for distinguishing different types of secondary structures (Supplementary Figure 1). The 101-protein data set is designed for direct comparison of our method with other popular methods for structural comparison (Supplementary Figure 1).

We selected nearly all available structures of proteases from PDB. To demonstrate the accuracy of our method on protein clustering, we chose proteases/hydrolases (1,872 structures) with at least 50 structures with a few of exceptions, e.g., plasmin because of the limited structures in PDB (Supplementary File 1). Fourteen CDK2 structures were selected from PDB, and the structures were trimmed from either N-terminus or C-terminus to make sure that all structures have identical amino acid sequences, except for the point mutations or deletions specified (Supplementary File 3). The reason why we trimmed the CDK2 structures is to distinguish the effects of label changes on structures from conformational changes. 4,520 structures were selected from CATH database (Greene et al., 2007) based on the criterion that every structure has 151 to 200 amino acids in the secondary structures (Supplementary File 4). Similarly, 1,400 structure were chosen from SCOP database (Murzin et al., 1995) and every structure has 201–300 amino acids in either alpha helices or beta pleated sheets (Supplementary File 5). To directly compare our method with other protein structural comparison methods, we randomly selected 101 proteins from a total of 3,604 proteins with roughly similar amino acid numbers (from 217 to 281 aa) because this smaller data set allows us to examine each structure in great detail for interpreting the results from comparison of our method with popular methods (Supplementary File 7). We also purposely included as many protein subclasses as possible to increase the structural diversities. The proteins of this small data set are from 10 protein subclasses: serine proteases (chymotrypsin, trypsin, elastase, subtilisin, plasmin, prothrombin), kinases (SRC and CDK) and phosphatases (Ser/Thr phosphatase and Tyr phosphatase). CE (Shindyalov and Bourne, 1998) and TM-align (Zhang and Skolnick, 2005) servers allow pairwise sequence alignment and pairwise protein 3-D structural comparison. We have automated the all-against-all structural comparisons and were able to extract RMSD and Z-scores from the analyses using CE (Shindyalov and Bourne, 1998) and TM-align (Zhang and Skolnick, 2005). Sequence alignment and phylogenetic analysis were done using MEGA7 (Kumar et al., 2016). Artificial protein structures were generated using MODELER (Šali and Blundell, 1993).

### Molecular Dynamics Simulations of ERK1 and CDK8 Were Performed Using the Amber Package

Molecular dynamic (MD) simulations were based on the procedure described by Simmerling et al. (Simmerling et al., 2002). We have followed the procedure of our previous work to study molecular dynamics of ERK1 (Xu et al., 2019), CDK8 (Xu et al., 2014), and CDK8-CycC complex (Xu et al., 2014; Odoux et al., 2016). The prmtop and inpcrd files of phosphotyrosine 204 (Ptr204) of human ERK1 were generated based on literature (Homeyer et al., 2006; Steinbrecher et al., 2012). The initial structure of the unphosphorylated human ERK1 was built through MODELER (Šali and Blundell, 1993) using the phosphorylated ERK1 (PDB ID: 2ZOQ) (Kinoshita et al., 2008) as the template. We have built nitrotyrosine prmtop and inpcrd files of ERK1 using the Gaussian 09 and Amber software packages (Xu et al., 2019). The prmtop and inpcrd files of human CDK8 were generated based on the crystal structure of CDK8 (PDB ID: 3RGF) (Schneider et al., 2011). All calculations used Amber's all-atom force field (ff14SB) as implemented in Amber 16 software (Case et al., 2005). The SANDER and CPPTRAJ modules (Roe and Cheatham, 2013) of Amber were used, respectively, for computation and analysis. Specially, a total of 1,000 steps of initial energy minimization, including 500 steepest descent steps (ncyc = 500) followed by 500 conjugate gradient steps (maxcyc-ncyc) using a large cutoff (cut = 999 angstroms) and non-periodic simulation (ntb = 0), were performed to adjust the structures of CDK8 or ERK1. To give the system time to adjust as temperature is raised to the production temperature, the minimized system was slowly heated from 0 to 325 Kelvin (K) in seven increments of 50 K over 50 ps (5 ps for the first six steps and 20 ps for the seventh step). The equilibration MD simulations were conducted for a total of 5 ns at a constant 325 K. For CDK8-CycC simulations, the solvated CDK8-CycC complex was equilibrated by carrying out a short minimization, 50 ps of heating and 50 ps of density equilibration with weak restraints on the complex followed by 500 ps of constant pressure equilibration at 300 K. Finally, a total of 2 ns production simulation was performed. A representative 66 frames (structures) of CDK8 and ERK1 were extracted from the uniformed steps of the entire MD simulations (Supplementary File 2). We also built a different set by extracting representative frames from all ranges of RMSD to increase structure diversity.

### Visualization

Visualization of our protein structure clustering is based on Average Linkage (Ackerman and Ben-David, 2016) and k-means (Lloyd, 1982) clustering (Supplementary Figure 1). The complexity of multiple dimensional relations among 3-D structures is reduced and represented by Multidimensional Scaling (MDS) method (Kruskal and Wish, 1978) (Supplementary Figure 1). Clustal W module built in Vector NTI (Lu and Moriyama, 2004) was applied to conduct pairwise sequence alignments (Supplementary Figure 1). Structural images were prepared using the Visual Molecular Dynamics (VMD) package (Humphrey et al., 1996) (Supplementary Figure 1).

## Results

### Determining the Numbers of Bins for Theta and MaxDist for Key Generation

#### Calculate the Bin Boundaries Using Adaptive Unsupervised Iterative Discretization Algorithm

**Figure 2:**
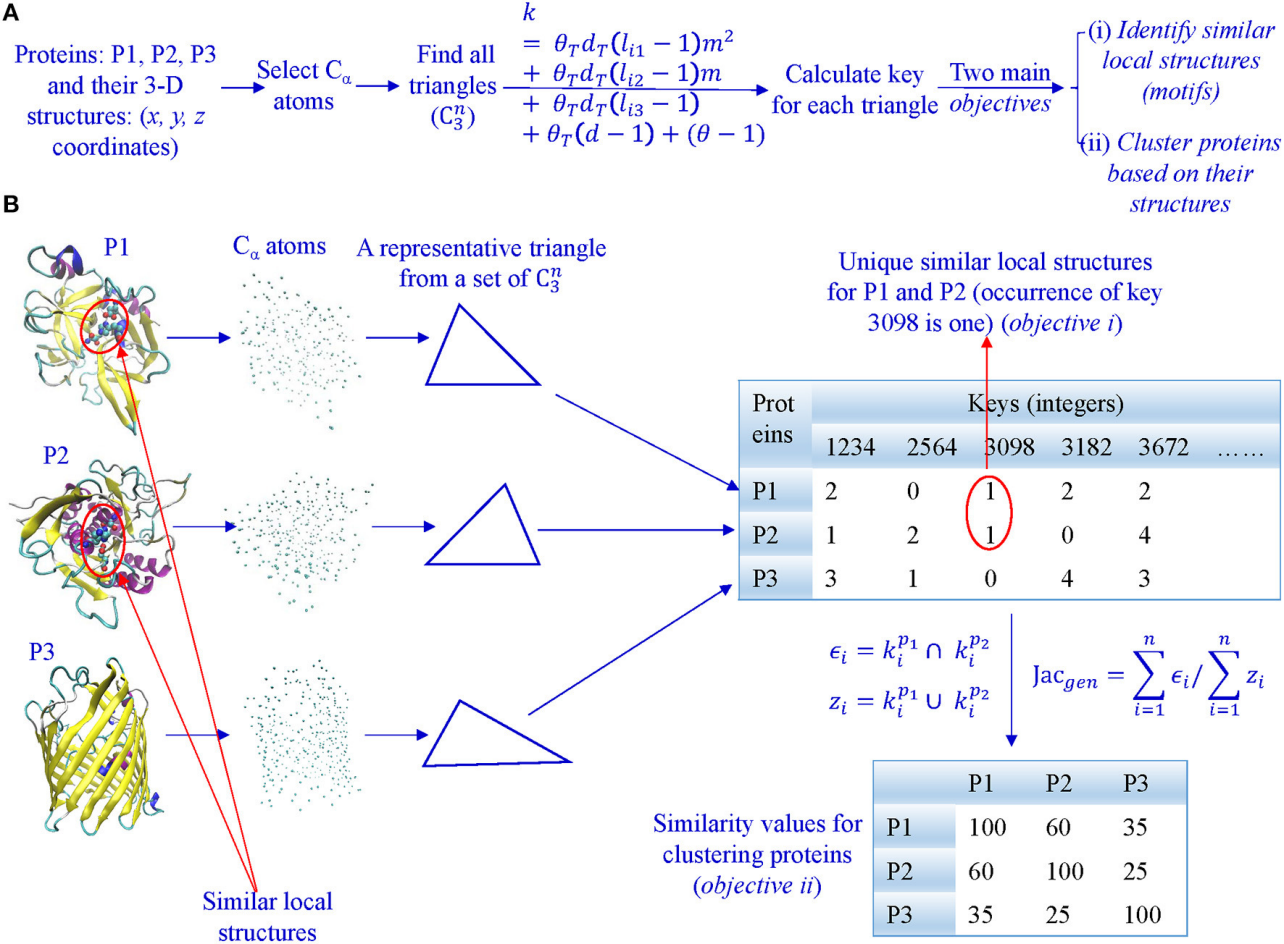
The overview of our TSR-based method for protein 3-D structural comparison at global and local levels. **(A)** It shows the steps involved in converting 3-D structures to keys, and objectives of our work; **(B)** All Cα atoms were selected from each of the representative 3-D structures, and lengths and angles of all possible triangles ($C_{3}^{n}$) were calculated. Each triangle is converted to an integer (a key) based on its lengths, angles, and amino acids. Consequently, each protein 3-D structure is represented by a vector of integers with their frequencies. A similarity matrix is calculated for clustering proteins, and identical keys with low frequencies in a certain class are found to be the candidates for motifs.

In our algorithm, we first select all C_α_ atoms and find all possible triangles formed by C_α_ atoms (Figures 2A,B). Second, we calculate keys using Equation 2 and key occurrence frequencies. Third, we quantify similarity or dissimilarity of two structures using the Generalized Jaccard similarity through computing identical and non-identical keys, and their frequencies (Figure 2) or the modified Generalized Jaccard similarity methods. Our approach does not require prior superimposition of 3-D protein structures and is customized to be sensitive to size of the triangles. The main objectives of our work are to do structure-based protein classification, and motif identification and discovery (Figure 2A).

To calculate meaningful keys, the foundation is to design an experiment to determine numbers of bins for Theta and MaxDist. To do so, we randomly selected 12 different non-overlapping sample sets from PDB, each containing 30–50 proteins. For each sample set, we calculated all angles and lengths. Theta-count plots show that count of triangles generally increases with the increase in the value of Theta (Supplementary Figure 2). The trend is the same, if we plot either all three angles against count or MaxDist against count (Supplementary Figure 3). Both the plots show skewed distributions. Based on the plots of Theta-count and MaxDist-count, we observed sample variations. We also learned that an equal width binning method will end up with a different number of triangles falling in each bin depending on whether the specified interval of values is for Theta or for MaxDist. To maximize the possibility of the same or similar number of triangles in each bin and to ensure that all occurrences of the same value are placed in the same bin, we used the Adaptive Unsupervised Iterative Discretization algorithm to calculate the bin boundaries (Liu et al., 2002; Witten et al., 2016). Within-bin variances of Theta and MaxDist for each sample set were calculated for different choices of total numbers of bins (Supplementary Figures 4, 5). The top five numbers of bins with the smallest variances were chosen for each sample set. The two with the greatest numbers of bins were selected from the top five numbers of bins (Supplementary Figure 6a), and then minimum, medium, and maximum numbers of bins and the numbers of bins with the highest frequencies were calculated (Supplementary Figure 6b). We identified the top three binning results for MaxDist as having numbers of bins: 12, 26, and 35 (Supplementary Figure 6c), and the top four binning results for Theta as having numbers of bins: 7, 15, 21, and 29 (Supplementary Figure 6d). Therefore, we have a total of 12 candidates of bin combinations.

#### Determine the Optimum Numbers of Bins From the 12 Candidate Bins Using the Small Testing Data Sets Selected From the Proteins With Their Functional Classifications as the Ground Truth

To further determine optimum numbers of bins for key generation, we came up with six small protein sample sets, each set containing 16–24 proteins in four different protein families with 4–6 members per family. We used all combinations of the four numbers of Theta bins and the three numbers of MaxDist bins to determine the best choice for combination of numbers of bins. If two or more combinations for the numbers of bins generate clustering that matches their functional classification, we chose the combination having higher numbers of the bins, since the latter allows us to distinguish small differences between the structures. It should be pointed that we also made minor changes on the final numbers of MaxDist and Theta bins mainly based on the fact that the physical interactions between two amino acids are generally stronger if they are closer. Our data shows that the combination, 29 bins for Theta and 35 bins for MaxDist produced the best result, in most cases, for clustering these six protein sample sets (Supplementary Figures 7–10). We found that the other combinations of Theta and MaxDist we tested can also correctly cluster these protein data sets (data not shown). To further verify that Theta 29 and MaxDist 35 are the optimum numbers of bin, we examined whether our method can cluster a large sample set correctly, and the result shows that the clustering of a total 178 proteins, from six families of about 30 proteins from each family, perfectly matches their functional classifications (Supplementary Figure 11, Supplementary Files 8,8A). The combination, Theta 29 and MaxDist 35, was used for majority of the analyses in the following sections. We have provided alternative bin boundaries, of three for MaxDist and four for Theta, as the choices for the investigators to test their own samples. Proteases, kinases, and phosphatases play essential roles in signal transduction (Hunter, 2000; Salvesen et al., 2016). Mutations of these enzymes are often associated with diseases, and they offer valuable targets in many therapeutic settings (Cohen, 2002; Tonks, 2006; Bond, 2019). In addition, the catalytic mechanism of serine proteases has been well-established (Carter and Wells, 1988; Dodson and Wlodawer, 1998). Therefore, we decided to employ our method in the study of proteases and kinases/phosphatases aimed for structure-based protein classification, as well as motif identification and discovery.

### Our TSR-Based Structural Comparison Method Can Be Used for Protein Clustering and Motif Discoveries

#### Application of Our Method in Clustering Proteases

**Figure 3:**
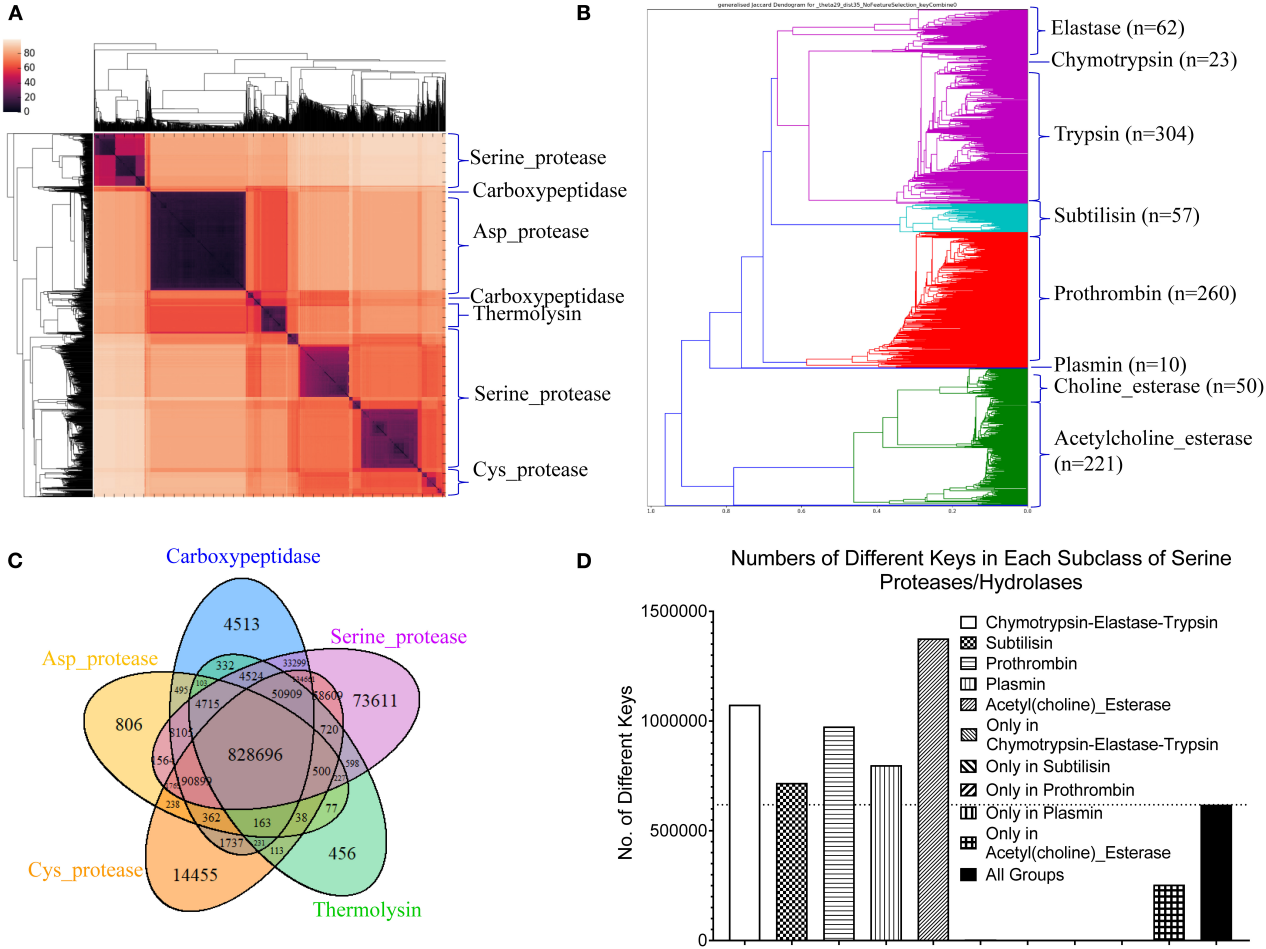
The clustering of proteases by our TSR-based structural comparison method. **(A)** The heatmap shows the cluster of proteases. The dissimilarity values are indicated in the upper left corner in all clustering heatmaps. The PDB IDs, and chain and class information can be found in Supplementary File 1. The complete list of the clustering result in the same order as the clustering map are provided in Supplementary File 1A; **(B)** The Venn diagram shows counts of the keys that are specific to each class of proteases, and all possibly overlapped regions among protease classes; **(C)** The dendrogram shows the clustering of the serine proteases. Number of the proteins in each subclass is indicated; **(D)** Total numbers of the keys and numbers of the specific keys to each class of serine proteases were calculated. Number of the common keys belonging to all classes of serine proteases were calculated. b and d, Key frequencies are not included for calculating the number of the keys corresponding to each class of proteases **(B)**, and each subclass of serine proteases **(D)**. Total distinct keys of a class were calculated from all the proteins in that class. These specific calculations are applied for (sub)classes of kinases and phosphatases. We designate the common keys at (sub)class levels.

Proteases hydrolyze peptide bonds of proteins, and were classified into four major classes: serine, cysteine, aspartate, and metal proteases (Rawlings and Barrett, 1993) before 1970; Now proteases extend to six distinct classes (López-Otín and Bond, 2008). Glutamate and threonine proteases are the two new classes. We found nearly all available structures of serine (986), aspartate (517), cysteine (131), and metal (105 carboxypeptidase and 133 thermolysin) proteases from PDB. We want to use this data set containing a total 1,872 structures as one of the case studies for demonstrating potential applications of our method in the area of protein structural comparison. More specifically, to evaluate performance of our method on protein structure-based clustering, we want to examine whether similar structures are clustered together while structures with low sequence and/or structure similarity are separated into different clusters. Our results show a nearly perfect clustering for aspartate, and cysteine proteases and thermolysins (Figure 3A) (Supplementary File 1A). Serine proteases were clustered into two subgroups, and carboxypeptidases were also clustered into two subgroups (Figure 3A). To find common keys belonging to all protease classes, specific keys for each class, and for two or more classes, we generated a Venn diagram (Figure 3B). The largest is the common key section, a total 828,696 distinct keys common to all classes, ranging from 59.5% (828,696/1,393,400) of total distinct keys for serine proteases to 92.9% (828,696/892,401) for thermolysins. The percentage of the keys specific to each class is small, ranging from 0.051% (456 out of 892,401) for thermolysins to 5.3% (73,611 out of 1,393,400) for serine proteases. This observation indicates that different classes share a large fraction of identical or similar triangles, and only small fraction of triangles is needed to distinguish one class from another.

Serine proteases can be divided into two types based on their functions: digestive system (chymotrypsin, elastase, trypsin, subtilisin), and regulatory system (thrombin, plasmin). We included acetylcholine and choline esterases in the study of serine proteases because of their nearly identical catalytic mechanism to serine proteases. Additionally, both acetylcholine and choline esterases, and serine proteases belong to the hydrolase family. They are 500–600 aa in size and larger than digestive and regulatory serine proteases (200–300 aa). We performed a deeper analysis on serine proteases. Our method shows eight clusters of serine proteases that agree with their functional classifications (Figure 3C). Our result shows the structures of chymotrypsin, trypsin and elastase are more similar. Serine proteases (or hydrolases) were separated into two groups in previous protease clustering. One of these two groups includes acetylcholine and choline esterases, and the other group contains digestive and regulatory serine proteases. Not surprisingly, the subclasses of serine proteases share a large fraction of common keys, and the number of the keys specific for each subgroup, except the group of acetylcholine and choline esterases, is small (Figure 3D). The exception for acetylcholine and choline esterases is probably due to their larger protein size. If we search for Common keys belonging to every protein of serine protease subclasses, except acetylcholine and choline esterases, only a very small fractions are Common keys regardless of whether we consider key frequency (2.4% out of total keys by average) or not (0.65% out of total different keys by average) (Supplementary Figure 12a). Those Common key have greater average Theta (Supplementary Figure 12b) and smaller average MaxDist (Supplementary Figure 12c) than the Uncommon keys. On an average, the frequency of those Common keys is two to three times higher than that of the Uncommon keys (Supplementary Figure 12d). In conclusion, our method is able to perform accurate clustering of serine proteases, and different subclasses share a high percent (59.5–92.9%) of the common keys. In contrast, only a small portion of the keys, called Common keys, are present in every protein, demonstrating high structural variations among proteins. The substructures corresponding to the Common keys have distinct features, e.g., Theta, MaxDist, and frequency, from those corresponding to the Uncommon keys.

#### Application of Our Method in Structure Motif Identification and Discoveries

**Figure 4:**
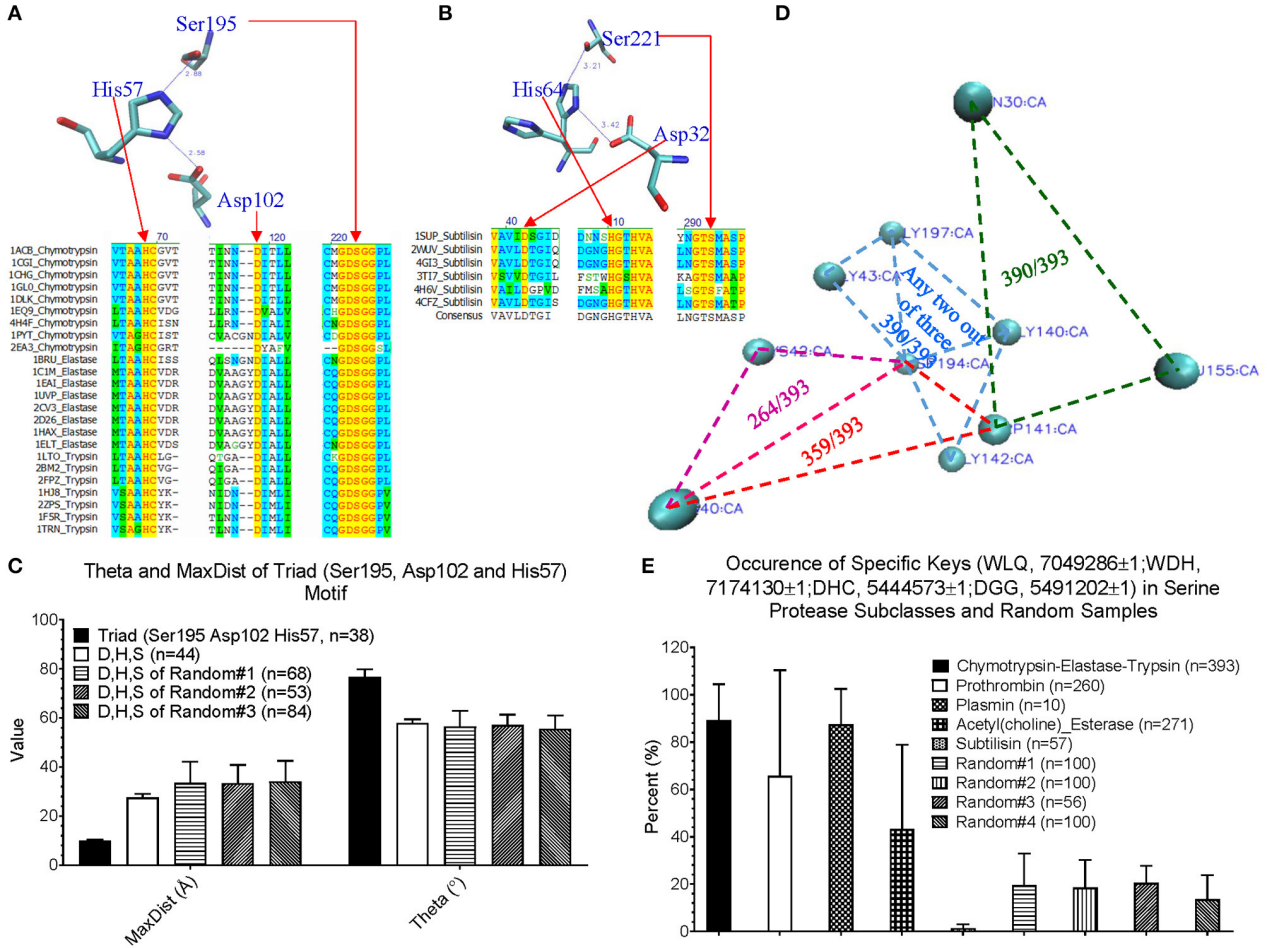
An impact of our method in motif identification and discovery of serine proteases. **(A)** The sequence alignment of the representative digestive serine proteases: chymotrypsin, trypsin and elastase, and a representative triad of chymotrypsin; **(B)** The sequence alignment of the representative subtilisins, and a representative triad of subtilisin; **(C)** The Theta and MaxDist for triad of serine proteases, and all the triangles formed by Asp, His, and Ser of the serine proteases and three sample sets randomly selected from PDB were calculated. Average, standard deviation and the number of proteins are indicated; **(D)** The representative triangles corresponding to the keys: 7049286 (green), 7174130 (red), 5444573 (pink), and 5491202 (light blue) are shown (PDB ID: 4H4F). Numbers of chymotrypsin-elastase-trypsin out of a total 393 having the keys are shown; **(E)** The percentage of occurrence of the keys: 7049286, 7174130 5444573, and 5491202 were calculated. Average, standard deviation and number of proteins are indicated. Key value difference by 1 (±1) allows minor flexibility for Theta to be considered as presence of the given key. This applies for all other figures where ±1 is specified.

Before applying our method for new motif discovery, we want to see whether our method can successfully identify known motifs. The active site, triad, of serine proteases has been well-studied (Rawlings and Barrett, 1994; Blow, 1997; Dodson and Wlodawer, 1998). It contains three amino acids: His57, Asp102, and Ser195 for human chymotrypsin (PDB ID: 4H4F) (Batra et al., 2013). Trypsin and elastase have corresponding His, Asp and Ser residues that can be aligned well with chymotrypsin (Figure 4A). However, subtilisin (PDB ID: 1SUP) (Gallagher et al., 1996) has an identical triad (Asp32, His64, and Ser221), but different order and positions at the amino acid sequence level (Figure 4B). We calculated the keys for the triad of chymotrypsin, trypsin, elastase and subtilisin, and they all have identical or nearly identical keys, demonstrating the success of our method in the identification of triad. If we use fewer numbers of bins for either Theta or MaxDist, the key for triad of serine proteases may not be unique. Next, we asked a question “What are the unique features of the triad triangle compared with all other triangles formed from His, Asp and Ser?” To answer it, we calculated Theta and MaxDist for the triad and for all possible His-Asp-Ser triangles. Our calculations show that the triad has a much shorter MaxDist and larger Theta than the average of all possible His-Asp-Ser triangles of serine proteins, and a similar relationship is noticed with respect to three protein samples randomly selected from PDB (*p* < 0.001, ANOVA) (Figure 4C).

We are more interested in demonstrating the ability of our method in discovering new motifs. The success of our study on triad provides a foundation for the next step of new motif discovery. Amino acid sequences of digestive, regulatory serine proteases and (acetyl)choline esterases are diverse and no amino acids are conserved (Supplementary Figure 13a). At the structural level, we found four distinct keys, and one of them appearing twice: one key of 7,04,9286 (Trp-Leu-Gln), one key of 7,17,4130 (Trp-Asp-His), one key of 5,44,4573 (Asp-His-Cys), and two keys of 5,49,1202 (Asp-Gly-Gly). A representative of these keys of a serine protease (PDB ID: 4H4F) (Batra et al., 2013) is shown is Figure 4D, and the amino acids corresponding to these five keys are between two β pleated sheets and not at the protein surface. These five keys are present in a high percentage of digestive serine proteases (*p* < 0.001 for Chymotrypsin-Elastase-Trypsin and Plasmin, and *p* < 0.0267 for prothrombin, ANOVA) (Figure 4E). Specifically, they have a high frequency for 7,04,9286 (390 digestive serine proteases out of a total 393), 7,17,4130 (359/393), and 5,49,1202 (390/393), and a relative low frequency for 5,44,4573 (264/393) (Figure 4D). Plasmins also have a fairly high likelihood of containing these five keys; ~60% of prothrombins, and ~40% of acetylcholine and choline esterases have these five keys. In contrast, most subtilisins do not have them (Figure 4E). To demonstrate that those keys are specific for digestive serine proteases, we came up with four sample sets randomly selected from PDB, and found that ~20% or less of the proteins from the random samples have them (Figure 4E). Next, we looked at individual keys; the majority of prothrombins, plasmins, and (acetyl)choline esterases have the keys: 7,04,9286 and 5,49,1202 (Supplementary Figures 13b–e). About 30–60% of the prothrombins, plasmins, and acetylcholine esterases have 7,17,4130, while nearly all choline esterases do not have it. For the key 5,44,4573, ~80% of plasmins have it, but a majority of the prothrombins, and acetylcholine and choline esterases do not have it. Taken together, we conclude that the five keys are specific for digestive serine proteases. Their presence and the percentage of occurrence of the individual keys can distinguish subclasses of serine proteases. Because these five keys have the potential to be used as one of the features specific for serine proteases, we want to understand if structural relations exist among them. Based on our limited structural analysis (PDB ID: 4H4F) (Batra et al., 2013), we show a hydrogen bond between 5,44,4573 and 5,49,1202, and two hydrogen bonds between 7,04,9286 and 5,49,1202 (Supplementary Figure 13f), suggesting hydrogen bonds can bring the keys close.

### Our TSR-Based Structural Comparison Method Can Detect Subtle Conformational Changes Upon Binding of a Ligand or an Interacting Protein or Due to Post-translational Modifications or Point Mutations

We have demonstrated, using our protease data set, that our method can be used to identify known structural motifs and to discover new motifs. The unique key for the known motif, triad, is essential for the catalytic activity of serine proteases (Rawlings and Barrett, 1994; Blow, 1997; Dodson and Wlodawer, 1998). However, we are not able to directly tie the new motifs of proteases to their functions. The main objective of this section is to explore our method in understanding subtle conformational changes due to post-translational modifications, point mutations or binding of a ligand or a partner with the focus on interpreting the conformational changes in the context of biological functions. This section is organized as follows. First, we evaluate stability of our method in studying protein dynamics using theoretical structures built from MD simulations. Second, we reinforce what we have found from theoretical structures using experimentally solved protein structures. Third, we evaluate our method based on the clinical success of Gleevec, a drug therapy of chronic myeloid leukemia, that specifically inhibits BCR-ABL, but not c-Src which is a structurally similar kinase (Deininger et al., 2005).

#### Evaluate Stability of Our Method in Studying Protein Dynamics

To evaluate our method in studying dynamics of protein structures, we have built theoretical structures using the experimentally determined structures as the templates. We will focus on the discuss of two proteins: ERK1 and CDK8. ERK1 is an important kinase in Ras-Raf-MEK signaling (Lewis et al., 1998). We have recently demonstrated that Tyr210 of ERK1 can be nitrated by mass spectrometry (Zhang et al., 2019). The nitration induces a formation of a tyrosine nitration-dependent intra-residue hydrogen bond (Xu et al., 2019) and this post-translational modification leads to a novel CHIP-dependent ERK1 degradation pathway (Zhang et al., 2019). CDK8 and its partner CycC are the essential regulators of cell cycle (Xu and Ji, 2011; Poss et al., 2013). We have systematically investigated the effect of point mutations of CDK8 on local structures using MD simulation (Xu et al., 2014). We have previously reported that CDK8-CycC serves as a regulator linking dietary perturbations to lipid metabolism (Xie et al., 2015; Gao et al., 2018). Here, we will present clustering results, identify the structural motifs, and explain the dynamics of the structural motifs in the context of biological functions of the proteins.

**Figure 5:**
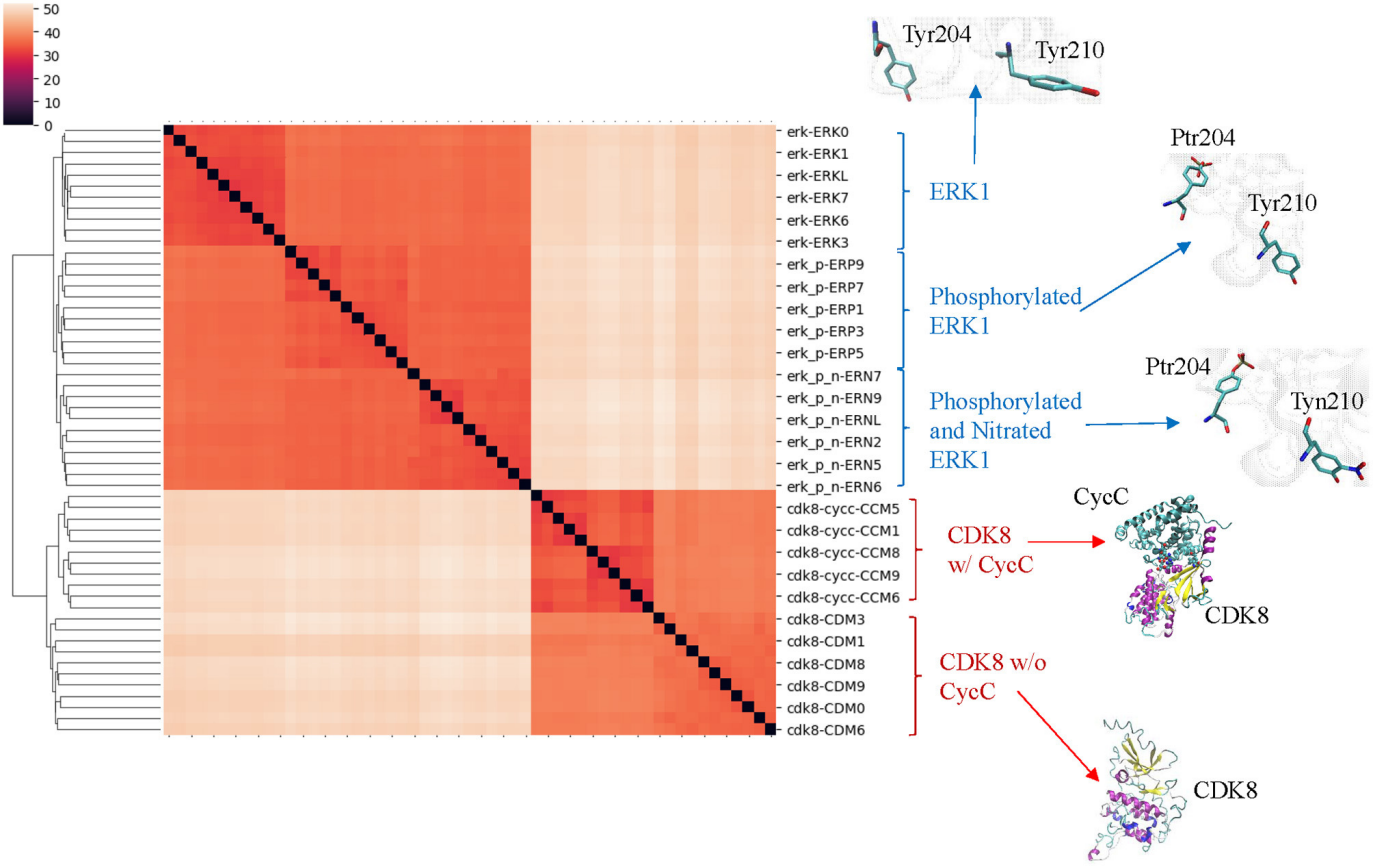
The clusters of ERK1 and CDK8 structures extracted from the MD simulations using our TSR-based structural comparison method match with their functional classifications. Phosphorylation site of ERK1 is at Tyr204, and phosphorylated Tyr204 is named as Ptr204. Nitration of ERK1 occurs at Tyr210, and nitrated Tyr210 is named as Tyn210. The amino acids with and without post-translational modifications at positions 204 and 210 of ERK1 are shown. The representative CDK8 structures with and without its partner CycC are shown. The dissimilarity values are indicated in the upper left corner in the clustering heatmaps.

**Figure 6:**
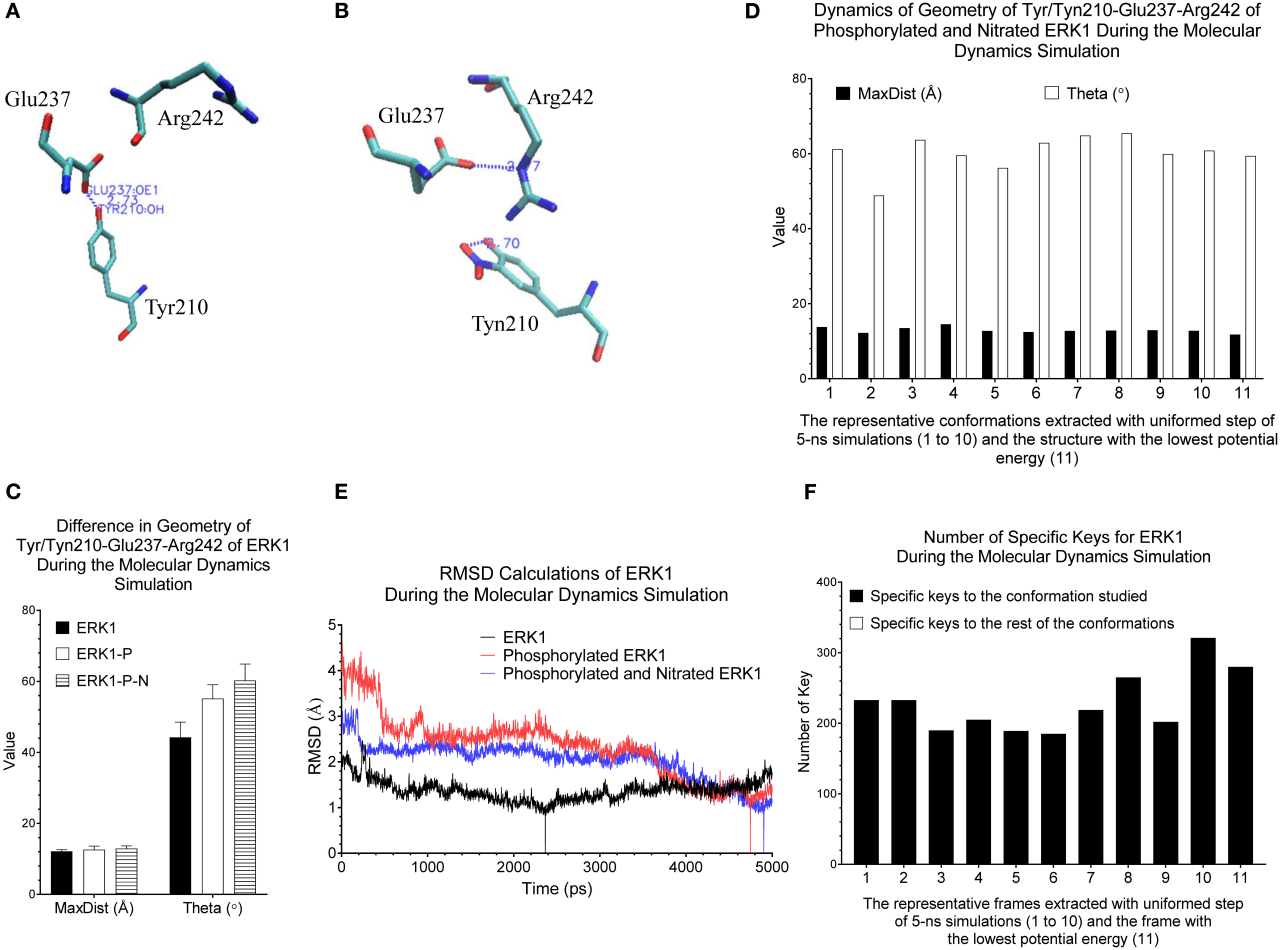
Our method can detect fine structural changes of ERK1 due to the post-translational modifications. **(A)** Three amino acids, Tyr210, Glu237, and Arg242, of the crystal structure (PDB ID: 2ZOQ) of ERK1 are shown. The hydrogen bond between Tyr210 and Glu237 is labeled; **(B)** The frame of ERK1-P-N having the lowest potential energy was extracted from 5-ns MD simulation. The intra-residue hydrogen bond between Tyn210 and Glu237, and the inter-residue hydrogen bind between Glu237 and Arg242 are labeled; **(C)** The MaxDist and Theta of the triangle of Tyr/Tyn/210-Glu237-Arg242 of ERK1, ERK1-P and ERK1-P-N were calculated. Mean ± SD are indicated; **(D)** Ten frames from the uniformed steps of 5-ns simulations were extracted and they are named as frames 1–10. The frame of ERK1-P-N with the lowest potential energy is labeled as frame 11; **(E)** RMSD values of ERK1, ERK1-P, and ERK1-P-N were calculated using their corresponding frames with the lowest potential energy, during 5-ns MD simulations, as the reference, **(F)** The numbers of specific keys exclusively belonging to each of the 11 frames (1–11) of ERK1-P-N were calculated and are presented. The numbers of specific keys exclusively belonging to all frames, except for the one frame indicated, were also calculated and are presented. The frame with the lowest potential energy is labeled as 11.

Our clustering result shows ERK1 and CDK8 are grouped into two major clusters (Figure 5). If we look closer at ERK1 and CDK8, ERK1 has three subclusters: ERK1 without phosphorylation and nitration (ERK1), phosphorylated ERK1 (ERK1-P), and phosphorylated and nitrated ERK1 (ERK1-P-N), whereas CDK8 has two subclusters: CDK8 with CycC and CDK8 alone (Figure 5). We have also noticed that the very early ~300 frames of ERK1-P during the simulation are separated from the early to late frames (from ~900 to ~5,000) of ERK1-P and are grouped with ERK1 together (data not shown). This could be due to the fact that the initial structure of ERK1 was built using ERK1-P as the template. The frames (from ~900 to ~5,000) of ERK1-P are well-separated from ERK1 and ERK1-P-N. Taken together, the results reveal that our method can detect subtle conformational changes due to the post-translational modification or binding of an interacting protein. Next, we asked a question “What unique local structures are exclusively belonging to ERK1, ERK1-P, or ERK-P-N?” Similarly, we want to know what the structural changes of CDK8 are caused by the binding of CycC. We will discuss ERK1 first and then CDK8. Tyr210 of ERK1 is hydrogen bonded to Glu237 (Kinoshita et al., 2008) (Figure 6A). Nitration of Tyr210 broke the hydrogen bond between Tyr210 and Glu237 due to a formation of intra-hydrogen bond after nitration occurred at Tyr210 (Xu et al., 2019; Zhang et al., 2019) (Figure 6B). Arg242 is close to Glu237 after the hydrogen bond between Tyr210 and Glu237 is broken (Xu et al., 2019) (Figure 6B). Therefore, we decided to study the substructure formed from Tyr/Tyn(nitrated Tyr)210, Glu237 and Arg242. We found that phosphorylation of Tyr204 and nitration of Tyr210 do not alter MaxDist, but increase Theta (Figure 6C). Very interestingly, phosphorylation at Tyr204 of ERK1 has an impact on the geometry of the triangle of Tyr/Tyn210-Glu237-Arg242. The dynamics of the triangle of Tyn210-Glu237-Arg242 of ERK1-P-N is shown in Figure 6D. All the representative structures have the same bin of MaxDist (5), but different bins of Theta (13, 16, 17, 18, 19, 20) (Figure 6D). It indicates that the triangle of Tyn210-Glu237-Arg242 of ERK1-P-N has larger or smaller bin labels of Theta depending on time points during the simulations (Figure 6D) although it has larger Theta on average than that of ERK1 and ERK1-P (Figure 6C). Figure 6E shows conformational changes in RMSD during the simulations using the frame with the lowest potential energy as the reference. To achieve a similar goal using a different representation from RMSD, our method illustrates conformational changes by calculating number of triangles exclusively for a particular frame over the period of the simulations (Figure 6F). We can also identify those triangles and map them in the 3-D structures. When we attempted to find triangles exclusively belonging to the rest of 10 frames, we nearly cannot find such triangles (Figure 6F), indicating high degree of conformational changes during the simulations.

**Figure 7:**
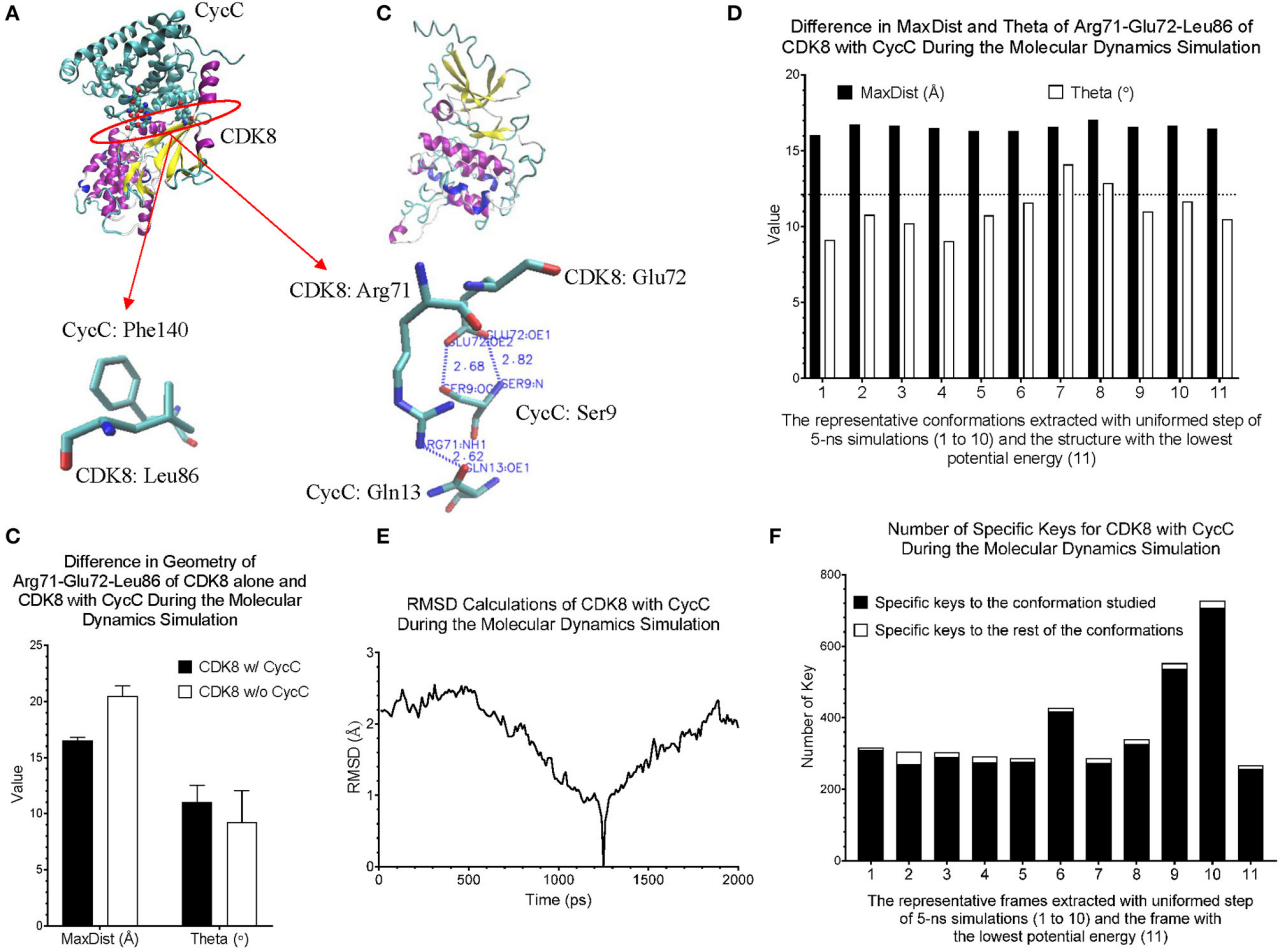
Our method can detect fine structural changes of CDK8 upon binding of CycC. **(A)** The crystal structure of CDK8 and CycC complex is shown (PDB ID: 3RGF). The interface between CDK8 and CycC is labeled. The hydrogen bonds and van der Waals interactions between CDK8 and CycC are shown; **(B)** The frame of CDK8 having the lowest potential energy was extracted from 5-ns MD simulation and is shown; **(C)** The MaxDist and Theta of the triangle of Arg71-Glu72-Leu86 of CDK8 alone and CDK8 with CycC were calculated. Mean ± SD are indicated; **(D)** Ten frames from the uniformed steps of 200-ps simulations were extracted and they are named as frames 1–10. The frame of CDK8 with the lowest potential energy is labeled as frame 11; **(E)** RMSD values of CDK8 in the complex of CDK8 and CycC were calculated using its frames with the lowest potential energy during 200-ps MD simulations as the references, **(F)** The numbers of specific keys exclusively belonging to each of the 11 frames (1–11) of CDK8 were calculated and are presented. The numbers of specific keys exclusively belonging to all frames, except for the one frame indicated, were also calculated and are presented. The frame with the lowest potential energy is labeled as 11.

CDK8 interacts with CycC through hydrogen bond and van der Waals interactions (Schneider et al., 2011). There are two hydrogen bonds between CDK8-Glu72 and CycC-Ser9, one hydrogen bond between CDK8-Arg71 and CycC-Gln13, and van der Waals interaction between CDK8-Leu86 and CycC-Phe140 (PDB ID: 3RGF) (Schneider et al., 2011) (Figure 7A). The structure of CDK8 with the lowest potential energy during the 5-ns simulation is shown in Figure 7B as the comparison with the CDK8 structure in the complex of CDK8 and CycC (Figure 7A). The triangle of Arg71-Glu72-Leu86 of CDK8 with CycC has smaller MaxDist and larger Theta than that of CDK8 without CycC (Figure 7C), indicating local conformational changes upon binding of CycC to CDK8. The calculations of MaxDist and Theta show the dynamics of triangles of Arg71-Glu72-Leu86 of CDK8 in the complex of CDK8 and CycC (Figure 7D). The triangles from frames 1–11 fall into the same bin (6) of MaxDist and two different bins (1, 2) of Theta. Frames 7 and 8 have Theta bin of 2 and the rest of the frames have 1 (Figure 7D). This subtle conformational change may not be able to be detected if we use fewer numbers of bins. The backbone RMSD plots show a gradual decrease in RMSD in the beginning stages of simulation and a gradual increase in the late simulation trajectories (Figure 7E). In contrast to showing conformational changes using RMSD, our method shows the number of specific keys for each frame. Our specific key calculations reveal more conformational changes in frames 6, 9 and 10 (Figure 7F).

#### Evaluate Stability of Our Method in Detecting Subtle Conformational Changes Upon Binding of a Ligand or an Interacting Protein or Due to Post-translational Modifications or Point Mutations

**Figure 8:**
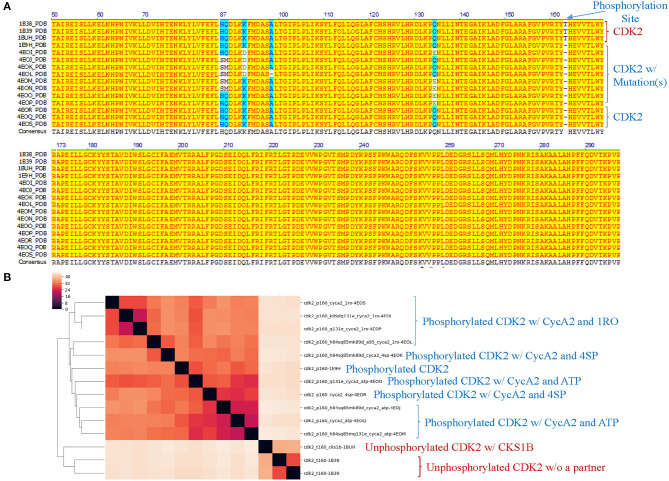
Our structure-based protein comparison method can detect conformational changes of CDK2 with the same/similar amino acid sequences due to post-translational modifications or upon binding of a ligand and a partner. **(A)** Fourteen CDK2 structures were found from PDB. The PDB structures were converted to amino acid sequences using pdb2fasta developed by Yang Zhang's lab. We trimmed the structures from N-terminus or C-terminus to make sure all CDK2 structures have the same amino acid sequence except mutations specified. The CDK2s with the mutations are labeled as well. The phosphorylation site at Thr160 is indicated. Note: phosphorylated Thr, TPO, is not shown in the amino acid sequence alignment; **(B)** The CDK2 structures are clustered using our method. p160 represents phosphorylation at Thr160 and t160 means Thr at the position of 160 (unphosphorylated). The CDK2 interacting proteins: cks1b and cyca2 are indicated. aa1xx(x)aa2 means aa1 at position of xx(x) was mutated to aa2, e.g., k89d means lysine at position 89 was mutated to aspartate. a95 indicates a deletion of alanine at position 95. Three ligands, ATP, 4SP, and 1RO, are labeled. a-b, Unphosphorylated CDK2 sequences are labeled in brown and phosphorylated CDK2s are labeled in blue.

**Figure 9:**
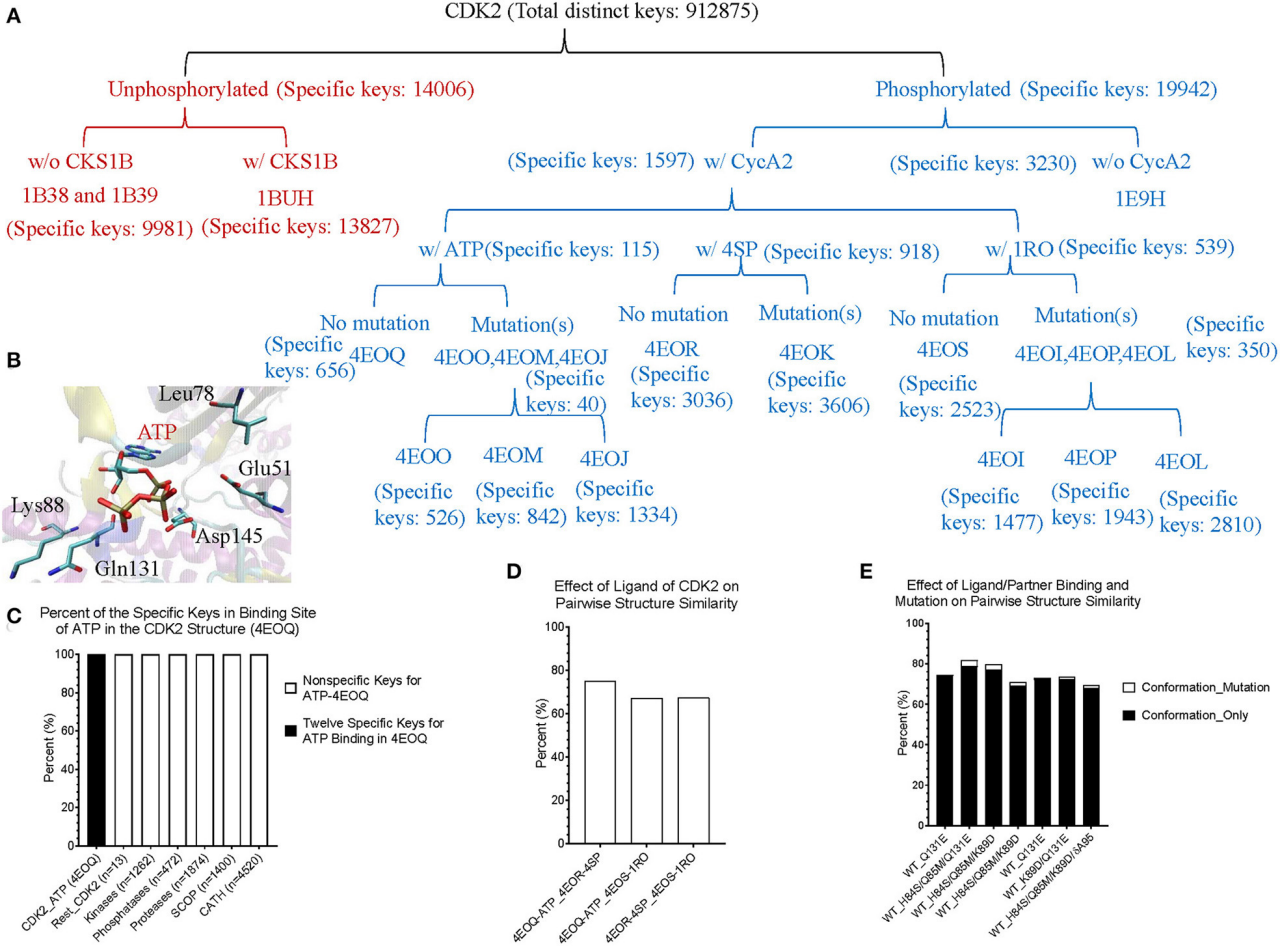
The results from the crystal structures of CDK2 reinforce that our structure-based protein comparison method can detect conformational changes of CDK2 with the same/similar amino acid sequences due to post-translational modifications or upon binding of a ligand and a partner. **(A)** It shows a hierarchical organization of CDK2 structures based on post-translational modifications, binding of a ligand or a partner, and with or without mutation(s). The total number of the distinct keys and numbers of specific keys for each (sub)group are identified and shown; **(B)** It shows ATP binding site of CDK2 (PDB ID: 4EOQ). Some of the amino acids associated with 12 specific keys for 4EOQ are shown; **(C)** The 12 keys specific to 4EOQ are shown. The identities of these 12 keys from 4EOQ can be found in Supplementary File 9. The PDB IDs, and chain and class information for kinases and phosphatases can be found in Supplementary File 10; **(D)** It shows pairwise percent similarity of CDK2 structures with identical amino acid sequence and without a mutation, but with different ligands: ATP, 4SP and 1RO; **(E)** It shows pairwise percent similarity of CDK2 structures with mutation(s) compared with their corresponding structures without mutations. WT, wild type means no mutation. Conformation_Only means structural comparison with a different label for each mutation and Conformation_Mutation means structural comparison with changing mutated label(s) back to their original one(s) for distinguishing the sources of dissimilarity.

Theoretical structures of ERK1 and CDK8 enable us to demonstrate the effect of post-translational modifications and binding of a partner on dynamics of local structures in the context of protein biological functions. Can we see similar effect from the experimentally determined structures? Point mutations often change local structures. Some mutations may have biological consequences while others may not. We assign each amino acid a different integer. As the result, either conformational changes or different labels will lead to a decrease in structure similarity. For this reason, we want to estimate what percent of difference between two structures is due to conformational changes caused by point mutations and what percent of difference is due to assignment of different labels. To achieve those goals, we have built a small data set of CDK2 by searching the structures in PDB. All the trimmed CDK2 structures have identical amino acid sequences except for the specified point mutations or deletion (Figure 8A). CDK2 structures can be divided into two major groups: phosphorylated and unphosphorylated. Our method clearly demonstrates two separated clusters of CDK2: one for the phosphorylated and the other for the unphosphorylated (Figure 8B). For unphosphorylated, there are two subgroups: one with an interacting partner, CKS1B, and the other without a partner, which can be distinguished by our method (Figure 8B). For phosphorylated CDK2, there are four major subgroups: without a ligand, with a ligand of ATP, 4SP, or 1RO. Each major subgroup can be further divided into minor subgroups: with or without point mutation/deletion. The result demonstrates a reasonably good clustering where the clusters match their functional classification except a mismatch for CDK2 with a ligand of 4SP (PDB ID: 4EOR) (Figure 8B). Next, we asked a question “Can we identify the substructures exclusively belonging to a given major group, a given major subgroup, and a given minor subgroup?” We have organized CDK2 data set in a hierarchical structure based on with or without post-translational modification, binding of a ligand or mutations/deletions (Figure 9A). Figure 9A shows the numbers of the specific keys belonging to each (sub)group. We asked a deeper question: “Can we link some of the specific keys we identified to their biochemical functions?” To address this question, we have focused on one structure: 4EOQ that is phosphorylated CDK2 without a mutation (Echalier et al., 2012). We found 12 out of 656 specific keys exclusively belonging to 4EOQ and the amino acids associated with these 12 keys are close to ATP (Supplementary File 9). Those amino acids close to ATP are shown in Figure 9B (PDB ID: 4EOQ). As expected, these 12 keys as a unique key set are only present in 4EOQ, CDK2 with CycA2 and ATP (Figure 9C). The rest 13 CDK2, other kinases (*n* = 1,262) (Supplementary File 10), phosphatases (*n* = 472) (Supplementary File 10), proteases (*n* = 1,872) (Supplementary File 1), the structures from CATH database (*n* = 4,520) (Supplementary File 4), and the structures from SCOP database (*n* = 1,400) (Supplementary File 5) do not have such 12-key set. Interestingly, three mutations: H84S/Q85M/Q131E, H84S/Q85M/K89D, and Q131E do not have this 12-key set although CycA2 and ATP are found in all these mutated CDK2 structures (Figure 9C). This result clearly demonstrates that single or multiple point mutations alter the geometries of the amino acids closely interacting with ATP. Now, we have learned that our method can detect local conformational changes induced by mutations. To determine percent of dissimilarity in structural comparisons caused by coordinate changes or label assignment changes due to mutation, we first compare the structure without mutations but with different ligands. They share 70–75% structure similarity (Figure 9D). It indicates that binding of different ligands induced 25–30% substructural changes. When we compared the structures containing the mutation(s) with their corresponding structures without the mutations, they share 73.06% similarity on an average (Figure 9E). It means that the mutation(s) lead to 26.94% local structural changes. If we assign the labels of the mutated amino acid(s) to the original one(s), but keep all coordinates unchanged, the similarity increased to 74.57% as predicted. Hence, on an average of 1.49% (74.57–73.06%) out of 26.94% structural changes is due to the label changes and the rest of 25.45% are due to mutation-induced actual structural changes.

#### Link the Subtle Conformational Changes Upon Binding of a Drug to the Drug Binding Site

**Figure 10:**
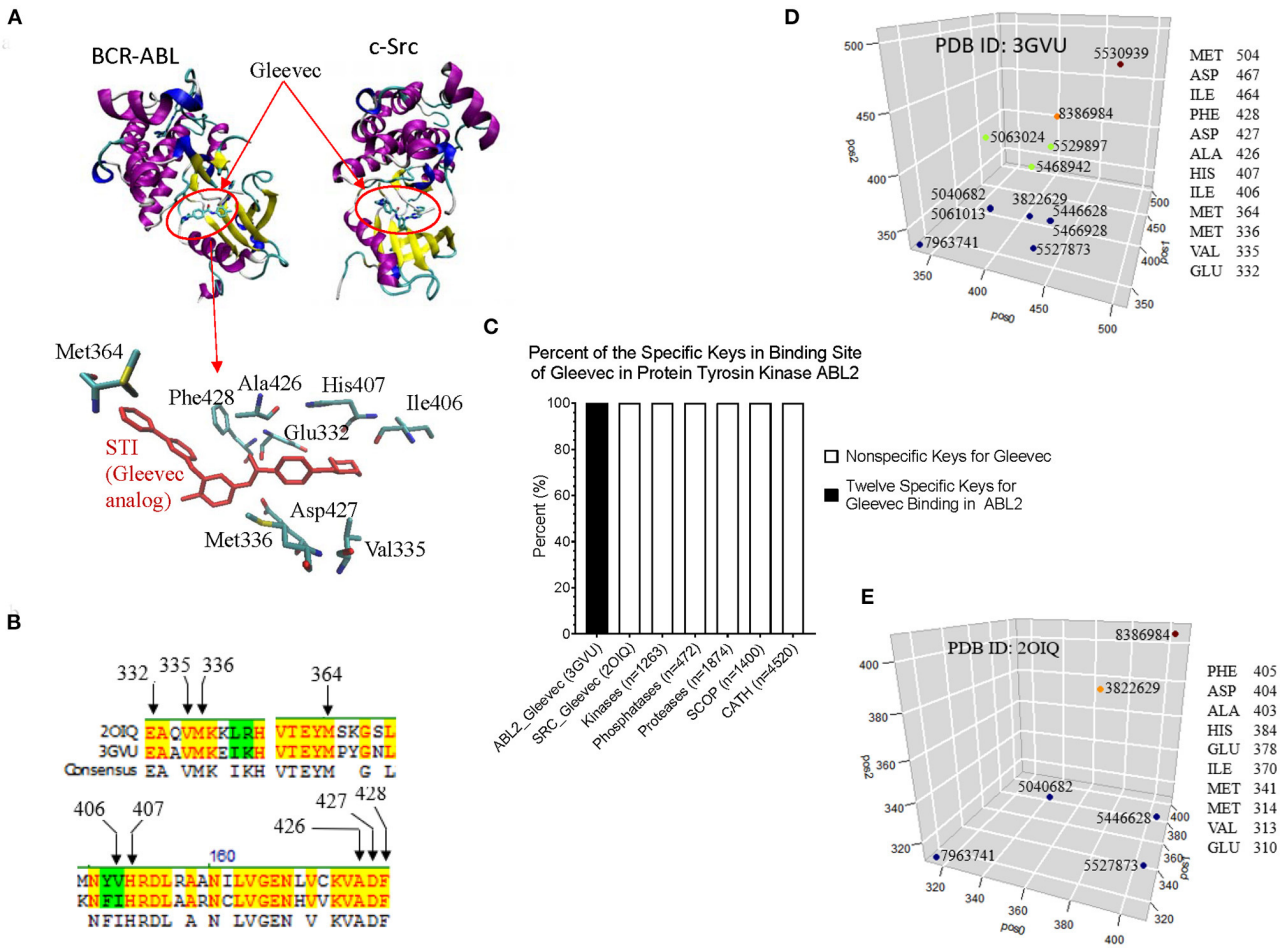
The example of interactions between BCR-ABL and Gleevec, and between c-Src and Gleevec reveals that our method can detect subtle structural differences associated with drug binding affinity. **(A)** It shows the Gleevec binding sites of BCR-ABL (PDB ID: 3GVU) and c-Src (PDB ID: 2OIQ). Some of the amino acids that have close interactions with Gleevec are shown; **(B)** It shows part of sequence alignment of BCR-ABL and c-Src. The amino acids in the Gleevec binding site are labeled; **(C)** The 12-key set shown is specific for BCR-ABL (PDB ID: 3GVU); **(D,E)** The identities of 12 keys specific for BCR-ABL **(D)** and 6 out of these 12 keys found in c-Src **(E)** are shown. The x (pos0), y (pos1), and z (pos2) are positions of three amino acids constituting triangles. pos0, pos1, and pos2 are determined by our rule-based formula.

From CDK2 set, we have shown that our method can detect conformational changes in the ligand bind site caused by the mutations outside the ligand binding site. We have not directly linked this finding to their functions. To more directly link the local structure to the function, we investigated the interactions between BCR-ABL and Gleevec, and between c-Src and Gleevec using our method. The clinical success of Gleevec is partly due to the restricted specificity of the drug that blocks BCR-ABL (Deininger et al., 2005). BCR-ABL and c-Src have similarity structures. The Gleevec binding sites of BCR-ABL and c-Src are similar, however subtle conformational changes can have a huge impact on drug binding. The abnormal fusion protein BCL-ABL is found in almost all patients with chronic myeloid leukemia. The IC_50_ value of Gleevec is 0.025–0.2 mM for BCR-ABL compared with > 100 mM for c-Src, indicating that Gleevec binds BCR-ABL 500–4,000 times stronger than c-Src (Deininger et al., 2005). This is the main reason why we have investigated BCL-ABL, c-Src, and Gleevec. Figure 10A shows the binding site of Gleevec of BCR-ABL and c-Src. The amino acids that interact with Gleevec are shown in Figure 10A and they are conserved in BCR-ABL and c-Src (Figure 10B). We identified that 12 keys as the key set from Gleevec-interacting amino acids are exclusively belonging to BCR-ABL (PDB ID: 3GVU) (Figure 10C). The identities of these 12 keys including amino acids and their positions, MaxDist, and Theta are included in Supplementary File 11. The 12-key set are not found in 1,263 kinases (BCR-ABL was excluded) (Supplementary File 10), 472 phosphatases (Supplementary File 10), 4,520 proteins from CATH (Supplementary File 4), and 1,400 proteins from SCOP (Supplementary File 5) (Figure 10C). Six of 12 keys are also found in c-Src [PDB ID: 2OIQ (Seeliger et al., 2007)]. The amino acids in the Gleevec binding site and their positions for 12 keys of BCR-ABL and 6 keys of c-Src are shown in Figures 10D,E, respectively.

### Our TSR-Based Structural Comparison Method Can Distinguish Alpha Helices From Beta Pleated Sheets

Secondary structure underpins the architectural organization in proteins (Lesk and Hardman, 1982; Konagurthu et al., 2012). In 1951, Pauling and Corey first defined two main secondary structural elements, alpha helix and beta sheet, based on the intra-backbone hydrogen bond patterns in proteins (Pauling et al., 1951). Based on structures in PDB, residues in known protein structures are ~30% in helices, 20% in strands and 50% in neither. To date, secondary structures have been extensively employed in structure visualization (Humphrey et al., 1996), classification (Murzin et al., 1995; Orengo et al., 1997; Sillitoe et al., 2015), comparison (Krissinel and Henrick, 2004; Shapiro and Brutlag, 2004a), and prediction (Holm and Sander, 1996). SCOP (The Structural Classification of Proteins) (Murzin et al., 1995; Brenner et al., 1996; Lo Conte et al., 2000) and CATH (Class, Architecture, Topology, Homologous superfamily) (Orengo et al., 1997; Greene et al., 2007) are two popular databases of domain structure-based hierarchical classification of proteins. Structure-based protein classification, compared with sequence-based approach, is able to detect distant relationships, because most protein folds are well-conserved (Tseng and Li, 2012) although domain folds cannot guarantee the identification of biological functions (Orengo et al., 1999). We will evaluate our method on clustering proteins with the focus on their secondary structures in this section.

#### Our TSR-Based Structural Comparison Method Can Distinguish Alpha Helices From Beta Pleated Sheets of the CATH Data Set

Four classes: mainly alpha, mainly beta, alpha beta, and few secondary structures are defined in the CATH database. With staying focus on secondary structures, we have chosen the structures from three out of four classes: mainly alpha (alpha), mainly beta (beta), and alpha beta (alpha-beta). We have selected 1,529 structures from the class of alpha that have a range of 151–200 amino acids in alpha helices. Similarly, we found 396 and 2,595 structures from the classes of beta and alpha-beta and those structures have 151–200 amino acids in beta pleated sheets, and either in alpha helices or beta pleated sheets, respectively. The MDS analysis shows that our method cannot distinguish alpha, alpha-beta, and beta (Supplementary Figure 14a). Because we cluster structures based on their shapes (secondary structures) not based on their functional classifications, we hypothesize fewer bins for MaxDist and Theta or assigning the same integer for structurally similar amino acids, e.g., serine vs. threonine, called amino acid grouping in this paper will improve clustering. To test these two hypotheses, we have chosen the fewest bins of MaxDist and Theta: 7 and 12, respectively from the 12 combinations of Theta and MaxDist bins discussed earlier (Supplementary Figure 6). We did not observe an improvement of clustering using Theta 7 and MaxDist 12 (Supplementary Figure 14b) compared with Theta 29 and MaxDist 35 (Supplementary Figure 14a), nor using amino acid grouping (Supplementary Figures 14c–e). We have also used hierarchical and k-means clustering methods and did not observe an obvious improvement by using fewer bins or applying amino acid grouping (data not shown). To understand why our method cannot distinguish alpha, alpha-beta, and beta, we generated Venn diagrams. The largest is the overlapped section of all three classes. This section has a total of 1,412,096 and 104,647 distinct keys, and occupies 96.6 and 98.5% of the total distinct keys (1,412,096/1,462,148) (Supplementary Figure 13f) and (104,647/106,267) (Supplementary Figure 14g) for Theta 29/MaxDist 35 and Theta 7/MaxDist 12, respectively. Small percentages of the keys were found to exclusively belong to alpha (2,725/1,462,148 = 0.19%), beta (4,961/1,462,148 = 0.34%) and alpha-beta (4,713/1,462,148 = 0.32%) classes for Theta 29 and MaxDist 35 (Supplementary Figure 14f). Using fewer bins dramatically decreases the number of total distinct keys from 1,462,148 of Theta 29/MaxDist 35 (Supplementary Figure 14f) to 106,267 of Theta 7/MaxDist 12 (Supplementary Figure 14g), indicating the difference in discretizing triangles by varying numbers of bins. We also observed the largest overlapped sections of all three classes by employing amino acid grouping (data not shown). Amino acid grouping also decreases the number of total distinct keys as expected (data not shown). The Venn diagrams explain why our method cannot discriminate alpha, alpha-beta, and beta.

**Figure 11:**
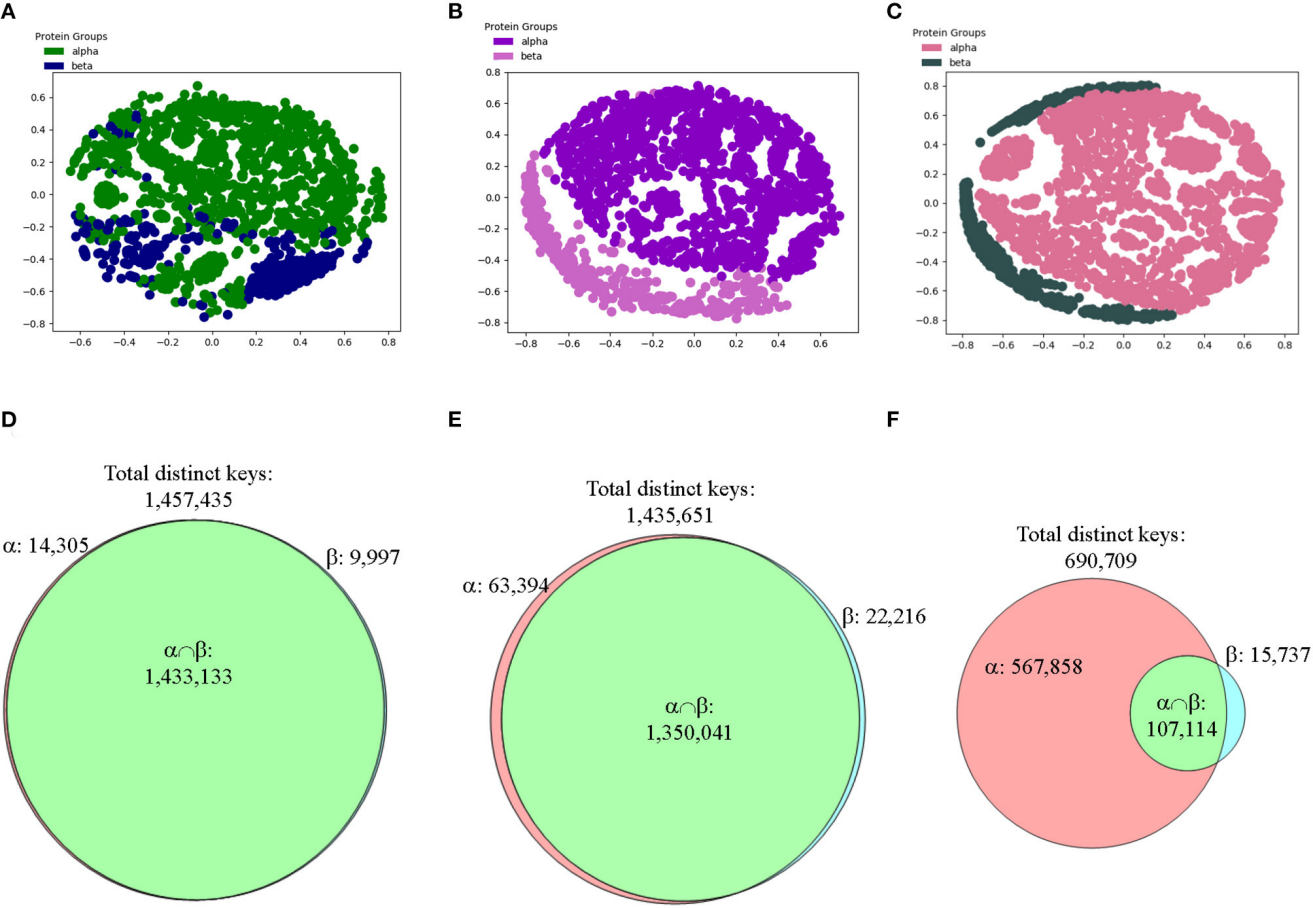
The results from the CATH data set show that our method can distinguish two main types of secondary structures: alpha and beta after applying feature selections. **(A–C)** The MDS analyses were used to show separation of structures from the CATH classes of alpha and beta that have 151–200 amino acids in alpha helices for alpha class or in beta pleated sheets for beta class without **(A)** and with feature selections (**B**, IESS; **C**, IASS). The detailed information, e.g., PDB IDs, chain and class information can be found in Supplementary File 4; **(D–F)** The Venn diagrams using Theta 29/MaxDist 35 show counts of the keys that are specific to each class, and are in the intersection of alpha and beta classes of the CATH data set prior to **(D)** and after feature selections (**E**, IESS keys; **F**, IASS keys). The numbers of total distinct keys, the specific keys for α and β, and the keys in the intersection are indicated.

Majority of the structures share ~20–40% similarity using Theta 29/MaxDist 35 (Supplementary Figure 14h), demonstrating structure diversity of the CATH proteins. Applying amino acid grouping or using fewer numbers of bins increases similarity (Supplementary Figure 14h) because the numbers of common keys increase compared with Theta 29 and MaxDist 35 (Supplementary Figure 14i) even when we did not account for decreases of total distinct keys. We did not observe significant changes when PCA was employed to analyze effect of applying amino acid grouping or using fewer number of bins (Supplementary Figure 14j). Since the structures in alpha-beta class contain helices and beta strands, we suspected helices in alpha-beta class could not be distinguished from helices in alpha class. This could be the similar situation for beta strands in classes of beta and alpha-beta. Thus, we came up with a more specific hypothesis that our method can distinguish alpha from beta, or *vice versa*. To test it, we have removed the structures in alpha-beta class from the CATH data set. The results demonstrated that our method still cannot distinguish the structures in alpha class from those in beta class (Figure 11A).

To focus on the substructure containing only helices and sheets, we decided to perform feature selections first and then perform clustering study. We define two different types of keys: intra-secondary structure (IASS) and inter-secondary structure (IESS), to make the procedure of our feature selection clear. IASS keys are defined as the keys from the triangles either within alpha helices or beta strands. IESS keys are defined as the keys from the triangles within and between secondary structure segments. IASS keys are a subset of IESS keys. After the feature selection, neither IASS nor IESS keys were able to distinguish three classes (Supplementary Figures 15a–c) even though the Venn diagram analyses clearly show gradual enrichment of α-, β-, αβ-specific keys after feature selections (Supplementary Figures 15d–f). However, alpha and beta classes are nicely separated if alpha-beta class is removed from the study and feature selection is applied (Figures 11A–C). The result from the CATH data set demonstrates that our method can distinguish alpha from beta, and *vice versa*. We observed the increase in the numbers of specific keys belonging to either alpha or beta after applying feature selections (Figures 11D–F). The Venn diagrams clearly explain why the IASS or IESS keys can be used to distinguish two major types of secondary structures (Figures 11D–F).

#### Our TSR-Based Structural Comparison Method Can Distinguish Alpha Helices From Beta Pleated Sheets of the SCOP Data Set

**Figure 12:**
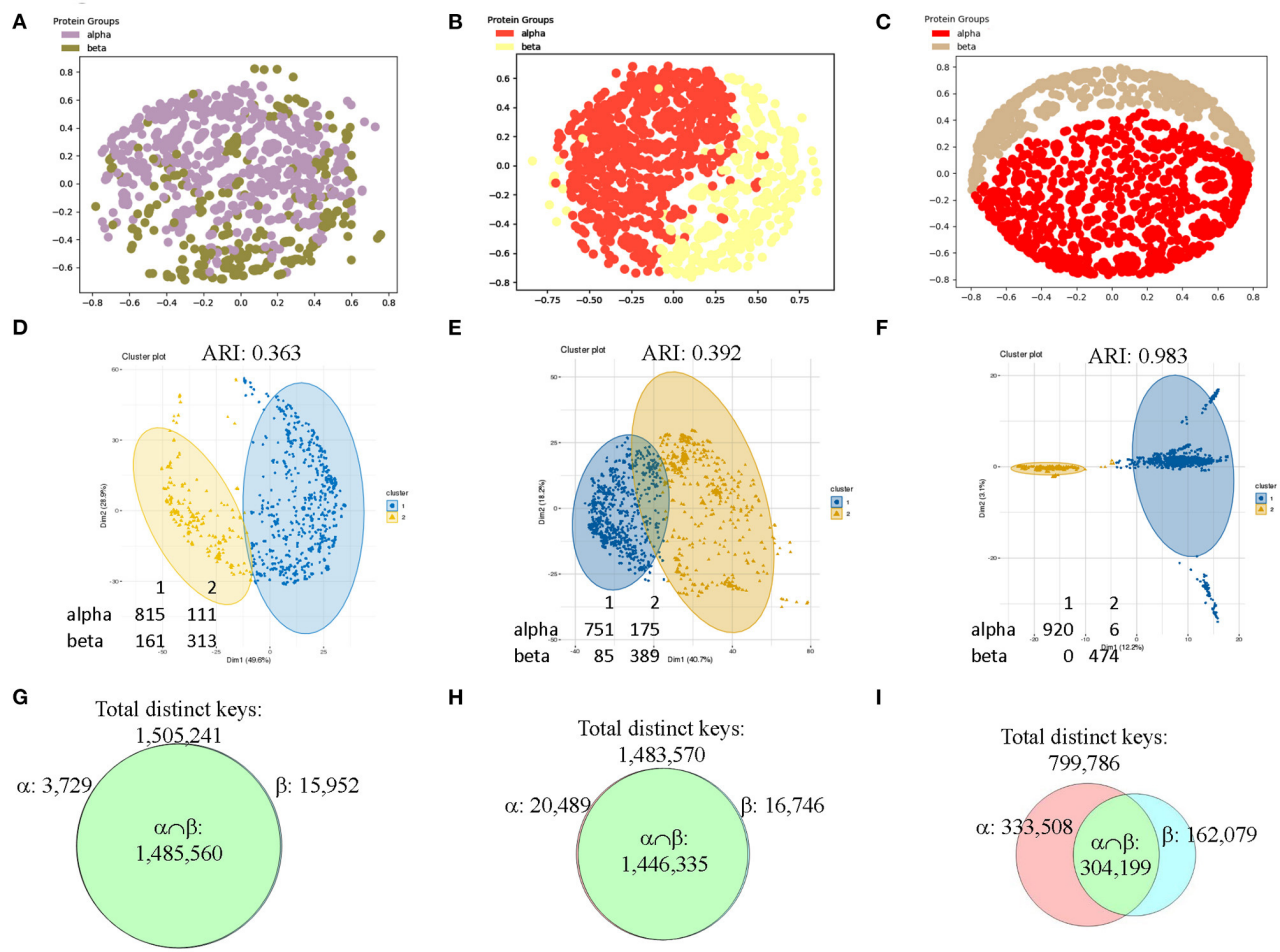
The results from the SCOP data set show that our method can distinguish two main types of secondary structures: alpha and beta after applying feature selections. **(A–C)** The MDS analyses were used to show separation of structures from the SCOP classes of alpha and beta that have 201–300 amino acids in alpha helices for alpha class or in beta pleated sheets for beta class without **(A)** and with feature selections (**B**, IESS; **C**, IASS). The detailed information, e.g., PDB IDs, chain and class information can be found in Supplementary File 5; **(D–F)** The structures studied in a-c were also investigated by k-means clustering method without **(D)** and with feature selections (**E**, IESS; **F**, IASS). The numbers of the mismatched structures and Adjusted Rand Index (ARI) are indicated; **(G–I)** The Venn diagrams using Theta 29/MaxDist 35 show counts of the keys that are specific to each class, and are in the intersection of alpha and beta classes of the SCOP data set prior to **(D)** and after feature selections (**E**, IESS keys; F, IASS keys). The numbers of total distinct keys, the specific keys for α and β, the keys in the intersection are indicated.

To make sure the observations we found from CATH data set are generalizable, we decided to verify it using SCOP data set. Since we used the structures with 151–200 amino acids in the secondary structures from CATH data set, we decided to use different sizes of secondary structures for SCOP data set. We have chosen the structures from SCOP database that have 201–300 amino acids in alpha helices (926 structures) or in beta pleated sheet (474 structures). The result shows that our method can distinguish the structures from classes of alpha and beta if feature selections are applied prior to MDS analysis (Figures 12A–C). We also used hierarchical (data not shown) and k-means (Figures 12D–F) clustering methods and PCA (data not shown) on the SCOP data set. Clustering using k-means algorithm shows a gradual decrease in numbers of mismatched structures and an increase in Adjusted Rand Index (ARI) (Hubert and Arabie, 1985) (Figures 12D–F). ARI is used to evaluate the quality of the clustering by the different methods. Similarly, the Venn diagrams explain why the selected feature set works for SCOP data set as well (Figures 12G–I). The SCOP data set contains structures with diverse amino acid sequence similarity. To further determine effect of amino acid sequence similarity on clustering, we have performed k-means clustering and PCA to compare the structures having low sequence similarity (<40%) (Supplementary File 12) with those having high sequence similarity (40–95%) (Supplementary File 13). The k-means analysis (Supplementary Figures 16a–f) and PCA (Supplementary Figures 17a–f) show no obvious differences between the data sets having structures with low and high sequence similarities.

#### Our TSR-Based Structural Comparison Method Can Distinguish Alpha Helices From Beta Pleated Sheets of the DD Data Set

To further confirm the conclusions based on from CATH and SCOP data sets, we decided to choose a data set from literature. DD data set was proposed by Ding and Dubchak (Ding and Dubchak, 2001) and modified by Shen and Chou (Shen and Chou, 2006). Since then, DD data set has been used in many protein fold classification studies. There are 311 protein sequences in the training set and 386 protein sequences in the testing set with no two proteins having more than 35% of sequence identity. The structures in DD data set were selected from different structural classes containing α, β, α/β, and α + β. We have used a subset of DD data set by choosing the structures only from α and β classes (Supplementary File 6). We obtained the same conclusion. Taken together, we conclude that our method can distinguish between two main types of secondary structures and a feature selection is needed for such purpose.

## Discussions

### Comparison of Our TSR-Based Method With Other Methods

Approximately 200 papers have been published on structural comparison/alignment since 1980. Among these algorithms, DALI (Holm and Sander, 1993), SSAP (Orengo and Taylor, 1996), CE (Shindyalov and Bourne, 1998), TM-align (Zhang and Skolnick, 2005; Xu and Zhang, 2010), VAST (Gibrat et al., 1996), PrlSM (Yang and Honig, 2000), SSM (Krissinel and Henrick, 2004) LOCK (Singh and Brutlag, 1997)/LOCK 2 (Shapiro and Brutlag, 2004b), ASSAM/SPRITE (Nadzirin et al., 2012), IMAAAGINE (Nadzirin et al., 2013), RASMOT-3D PRO (Debret et al., 2009), and SPASM (Kleywegt, 1999) have been widely used. We have compared our method with other methods in two different ways: one way is to study the data sets from the published papers, and the other way is to study the data sets we have generated using our and the published methods.

#### Comparison of Our TSR-Based Method With Other Methods Using the Data Sets From the Published Papers

Hou and his colleagues constructed a map of the “Protein Structure Space” by using the pairwise structural similarity scores and found that Prdx2 (PDB ID: 1QMV, Chain A) and ArsC (PDB ID: 1J9B, Chain A) have similar structures (Hou et al., 2005), and both belong to the GO 0016491 (oxidoreductase). Hou et al. stated that the DALI algorithm will assign them as structurally different proteins (similarity score: 242.3, Z-score:1.7, RMSD: 3.5 Å) (Hou et al., 2005). The sequence alignment shows that Prdx2 and ArsC have low amino acid identity and high similarity (Supplementary Figure 19a). The clustering result generated using our method agrees with their functional classification (Supplementary Figure 19b). These two types of oxidoreductases share a large fraction of the common keys at Prdx2-ArsC level (Supplementary Figure 19c) and a smaller fraction of the Common keys at the individual protein level (Supplementary Figure 19d). The Common keys have shorter MaxDist and higher Theta and key frequency values compared to all keys and to the Uncommon keys (Supplementary Figure 19e). Holmes and her colleagues found that Actin (PDB ID: 1ATN) and Hsp70 (PDB ID: 3HSC) have similar structures although there is very little sequence identity between the two proteins (Flaherty et al., 1991). Our method shows these two classes share a very large section of the common keys at the class level (Supplementary Figure 20a) and a small fraction of the Common keys at the individual protein level (Supplementary Figure 20b). The cluster obtained matches their classifications by function (Supplementary Figure 20c) and sequence. It is not surprising that they have high amino acid similarity in contrast with a low identity (Supplementary Figure 20d). The Common keys have higher frequency than the Uncommon keys (Supplementary Figure 20e). Our method clearly shows four distinct clusters when we combined Arsc and Prdx2 with Actin and Hsp70. 65, 444, 521, and 5,371 distinct keys can be used to distinguish Prdx2, Arsc, Hsp70, and Actin, respectively from the rest of the classes (Supplementary Figure 21). “Protein Structure Space” and DALI methods consider α helices similar. In contrast, our method will show that α helices are different even though they have similar topology if they have different amino acid compositions. “Protein Structure Space” and DALI methods are designed to classify proteins based on their topology while our method is based on geometry and function. The specific keys we identified allow us to distinguish one sub(class) from others in a structure-based hierarchical organization of proteases. Even for the proteins with low amino acid similarity, we found a high percentage of the common keys at the (sub)class levels (Figures 3B,D). It suggests that the total variety of protein structures is considerably smaller than the variety of protein sequences, in agreement with the prediction from the literature (Hasegawa and Holm, 2009). It also indicates there is room for us to increase our numbers of MaxDist and Theta bins. In contrast to the high percentages of the common keys at the class level, we found low percentages of the Common keys at the individual protein levels (Supplementary Figure 12a), demonstrating diversity of protein structures. The unique key calculation, comparison and search features of our method allow not only building of hierarchical protein structure relations, but also provide crucial insights into the nature of protein structure relations at global and local levels. Additionally, identification of the keys specific to a certain (subclass) of proteins will help to achieve a specific therapeutic outcome by minimizing off-target toxicity.

#### Direct Comparison of Our TSR-Based Method With the Selected Popular Methods for Clustering Proteins

**Figure 13:**
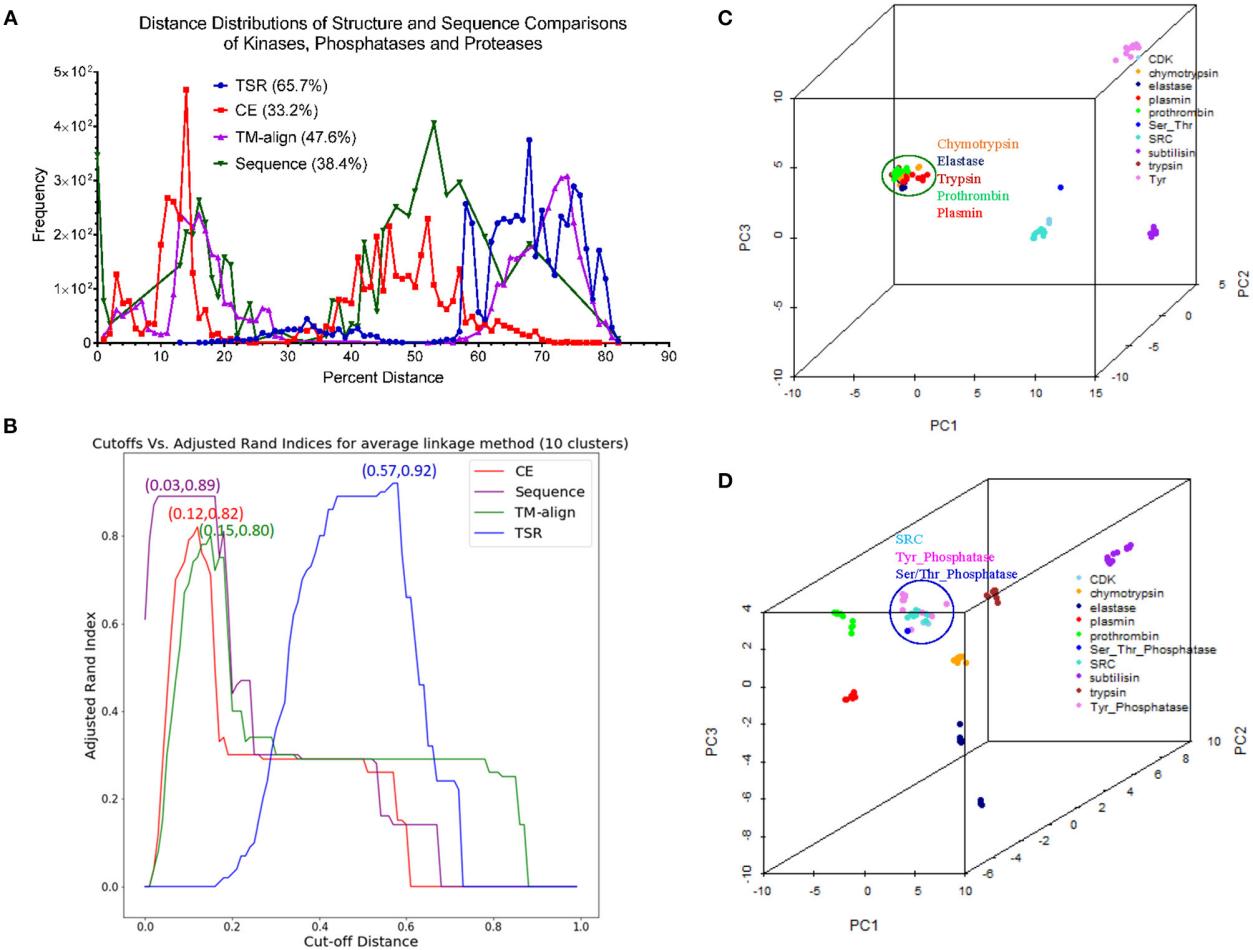
The studies of structure and sequence dissimilarity of proteases, kinases and phosphatases demonstrate differences between methods of structure and sequence comparisons. **(A)** The distance distributions of the different methods of structure and sequence comparison. Our method uses percentage to show the differences in structures while CE, TM-align use RMSD to show the structural differences. The sequence alignment uses genetic distance. The distance values were normalized to the percentage of distance. The weighted percent distances are indicated. The distance values of pairwise comparison using our method are provided in Supplementary File 14; **(B)** The plot shows relation between the cutoffs and ARI of different methods for the average linkage clustering method. The number of the clusters was set to ten. The cut-off distances were normalized to 1.0. The cutoff values for the best ARI are indicated; **(C)** The diagram shows the separation of the clusters using the first three principle components from the RMSD matrix generated by CE method. The protein structures clustered together are circled and labeled; **(D)** The diagram shows the separation of the clusters using the first three principle components from the distance matrix generated by our method. The protein structures clustered together are circled and labeled.

We created a small data set (101 protein structures) for the purpose of directly comparing our method with other popular methods of protein structural comparison [DALI (Holm and Sander, 1993), CE (Shindyalov and Bourne, 1998), and TM-align (Zhang and Skolnick, 2005)]. The clusters of the protein structures using DALI method nearly perfectly agree with their functional classifications except two separated clusters observed for elastases (Supplementary Figure 22). Interestingly, two clusters of elastase are also found in CE (Supplementary Figure 23), TM-align (Supplementary Figure 24) and our (Supplementary Figure 25) methods. Our method groups one Tyr phosphatase (PDB ID: 3ZMQ) with Ser/Thr phosphatase (PDB ID: 2DFJ) together (Supplementary Figure 25). Two separated clusters of plasmins are found in both CE (Supplementary Figure 23) and TM-align (Supplementary Figure 24) methods. An additional two separated clusters of chymotrypsin are found in TM-align method (Supplementary Figure 24). The RMSD matrices of this small data set were obtained from CE (Shindyalov and Bourne, 1998) and TM-align (Zhang and Skolnick, 2005) while RMSD matrix was not generated by DALI server (Holm, 2019). When we compared chymotrypsin (PDB ID: 1P2O) with subtilisin (PDB ID: 2ST1) using DALI server, we cannot obtain the output of RMSD or Z-score. It was said that a similarity value with Z-score lower than 2 is spurious. This prevents us from further comparison of our method with DALI method. Two separated elastase clusters found in all four structural comparison methods motivated us to perform multiple sequence alignment of these proteins. The Clustal W sequence alignment also shows two separated elastase clusters (data not shown). After sequence alignment using Clustal W, we used Neighbor-Joining algorithm to build a phylogenetic tree. The phylogenetic tree perfectly matches their functional classifications (Supplementary Figure 26). Two separated clusters of elastases are grouped together. Minor differences in clustering using different methods inspired us to further study distance distributions. CE, TM-align and sequence alignment exhibit two separated peaks in the plots of distance distributions (Figure 13A). In contrast, our method has only one major peak between 60–80% distance (Figure 13A). Although all the methods clustered protein structures reasonably well, our method (66.7%) has much larger percent weighted distance than TM-align (47.6%), sequence alignment (38.4%) and CE (33.2%) methods (Figure 13A). The larger weighted distance value indicates that our method can identify finer substructural differences from two or more similar structures considered by other methods. We also noticed that significantly different protein sequences have distance of zero (Figure 13A), suggesting redundancy at the protein sequence level. However, those proteins with distance of zero in sequences do not have distance of zero in the structural comparison (Figure 13A) (Supplementary File 14), suggesting more diversity in protein structures than sequences. To gain better insights into the comparisons among the methods, we used a manual cutoff, for every method under test, that maximizes the ARI (Hubert and Arabie, 1985; Yeung and Ruzzo, 2001) with respect to the specified number of clusters. Based on the ARI values, our method (0.92, the highest ARI) is better than CE (0.82, the highest ARI) and TM-align (0.80, the highest ARI), and CE performs slightly better than TM-align (Figure 13B). We also noticed that our method has much the larger normalized cutoff (0.57) than other methods (0.15, 0.12, 0.03) to achieve the best ARI (Figure 13B). This reinforces the observation from the distance distributions that our method generally exposes finer distinctions in clustering structures than other methods. To gain deeper understanding of cluster compactness between the different methods, we performed a principle components analysis (PCA) (Jolliffe, 1986) through reducing dimensionality of the distance matrices. The first four principle components capture most of the variation of the distance matrices of both CE (92.5%) and TM-align (98.0%) methods (Supplementary Figure 27a). In contrast, 92.5 and 62.4% of the variations require 10 principle components for our method and sequence alignment, respectively (Supplementary Figure 27a). We found the serine proteases except subtilisin are closely clustered together in CE (Figure 13C), TM-align (Supplementary Figure 27b) and sequence comparison (Supplementary Figure 27c). In contrast, all the clusters of the serine proteases are well-separated in our method (Figure 9D). Interestingly, the clusters of the kinases and phosphatases are closer to each other in our method (Figure 13D) than other methods (Figure 13C and Supplementary Figures 27b,c). Kinases and phosphatases were not clustered well by the PCA (Supplementary Figure 27c) compared with the sequence analysis using Clustal W and Neighbor-Joining algorithms (Supplementary Figure 26). Some of the serine proteases share high sequence similarity while kinases and phosphatases do not have high sequence similarity. Taken together, clustering using CE and TM-align methods appears to largely depend on sequence similarity while clustering using our method depends more on similarity of substructures than sequence similarity.

#### Applying Mirror Image in Our Key Calculation Formula Improves the Ability to Identify Substructures Specifically for a Certain sub(class) of Proteins

**Figure 14:**
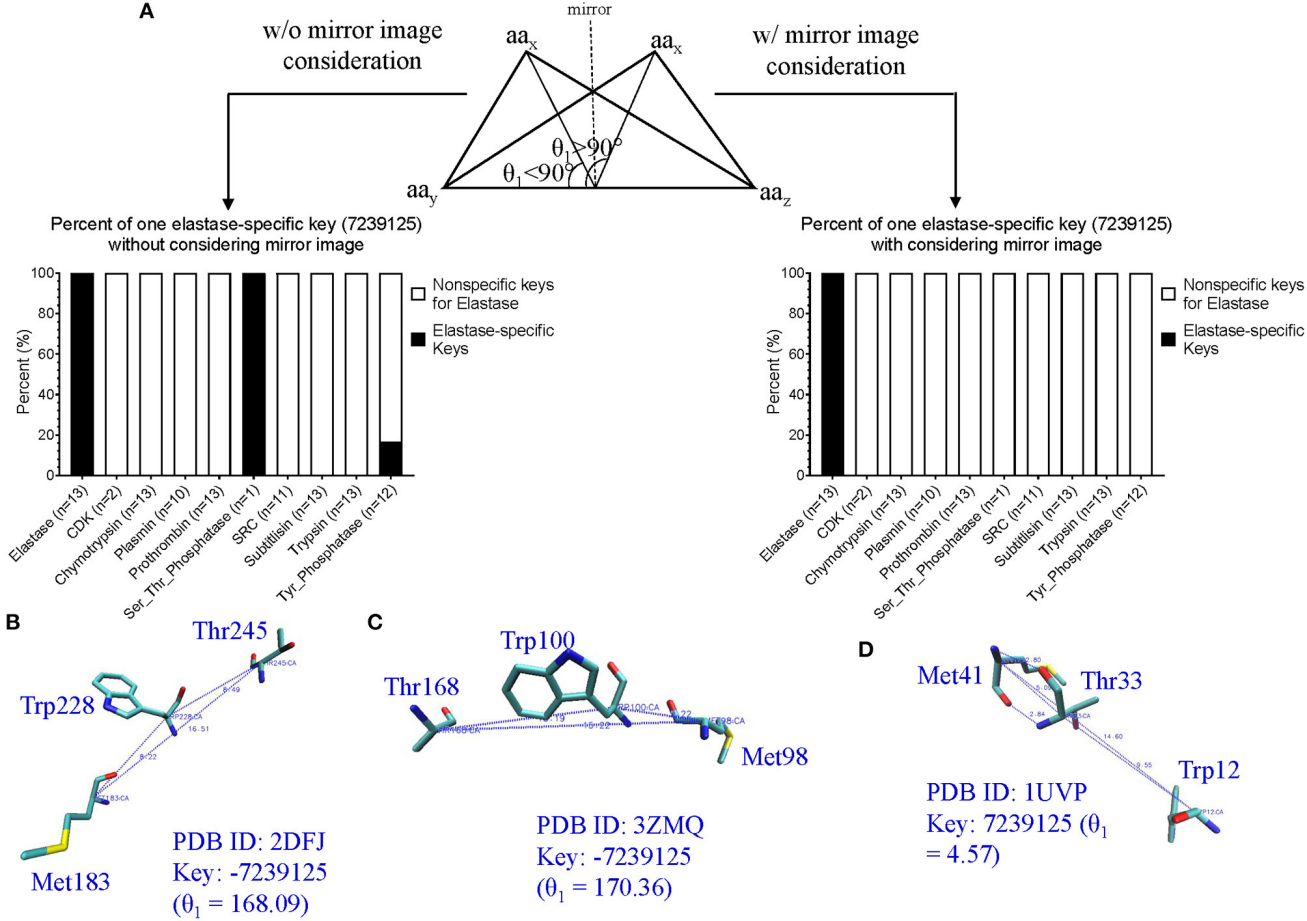
The effect of the mirror-image keys on specific keys for elastases. **(A)** Two triangles are considered as mirror images if they meet the criteria: (i) the same amino acids, (ii) having θ_1_ < 90° for one triangle and θ_1_ > 90° for another triangle, (iii) the bin is the same for θ of the triangle with θ_1_ < 90° and 180°-θ of the triangle with θ_1_ > 90°. The specific keys are represented as percentage; **(B–D)** The representative keys are shown for Ser/Thr phosphatase, Tyr phosphatase and elastase, respectively. The PDB IDs, θ_1_, and keys are indicated.

As stated earlier, our method compares every substructure of one protein with every substructure in a different protein. This feature allows us to calculate common and specific substructures for deeper understanding of protein structure clusters. In addition, two separated clusters of elastases observed in our structural comparison inspired us to check whether we can identify unique substructures specifically belonging to elastases. We were able to identify unique substructures for each protein subclass except for elastases (Supplementary Figure 28). This observation indicates structure diversity of elastases. In our original key calculation formula, the two angles will be assigned to the same bin if Theta is equal to 180°–Theta. Thus, the two triangles are assigned to the same key if they are mirror images. To further discretize keys, we assign two mirror-image triangles two different keys. If Theta is <90, key is a positive integer. Otherwise, key is a negative integer with the same value as its mirror-image triangle (Figure 14A). After applying mirror image in our key calculation formula, we were able to identify one unique key (7,23,9125) exclusively belonging to elastases (Figure 14A). The mirror-image triangles are found in Ser/Thr and Tyr phosphatases and they have the key of −7,23,9125 (Figure 14A). The three amino acids (Met, Trp, and Thr) of ±7,23,9125 from a representative of Ser/Thr phosphatase, Tyr phosphatase, and elastase each are shown in Figures 14B–D and those amino acids are at the protein surfaces. Two hydrogen bonds are observed in key of 7,23,9125 but no hydrogen bonds are found in key of −7,23,9125.

#### Our TSR-Based Method Is Relatively Insensitive to Substructure Rearrangement

**Figure 15:**
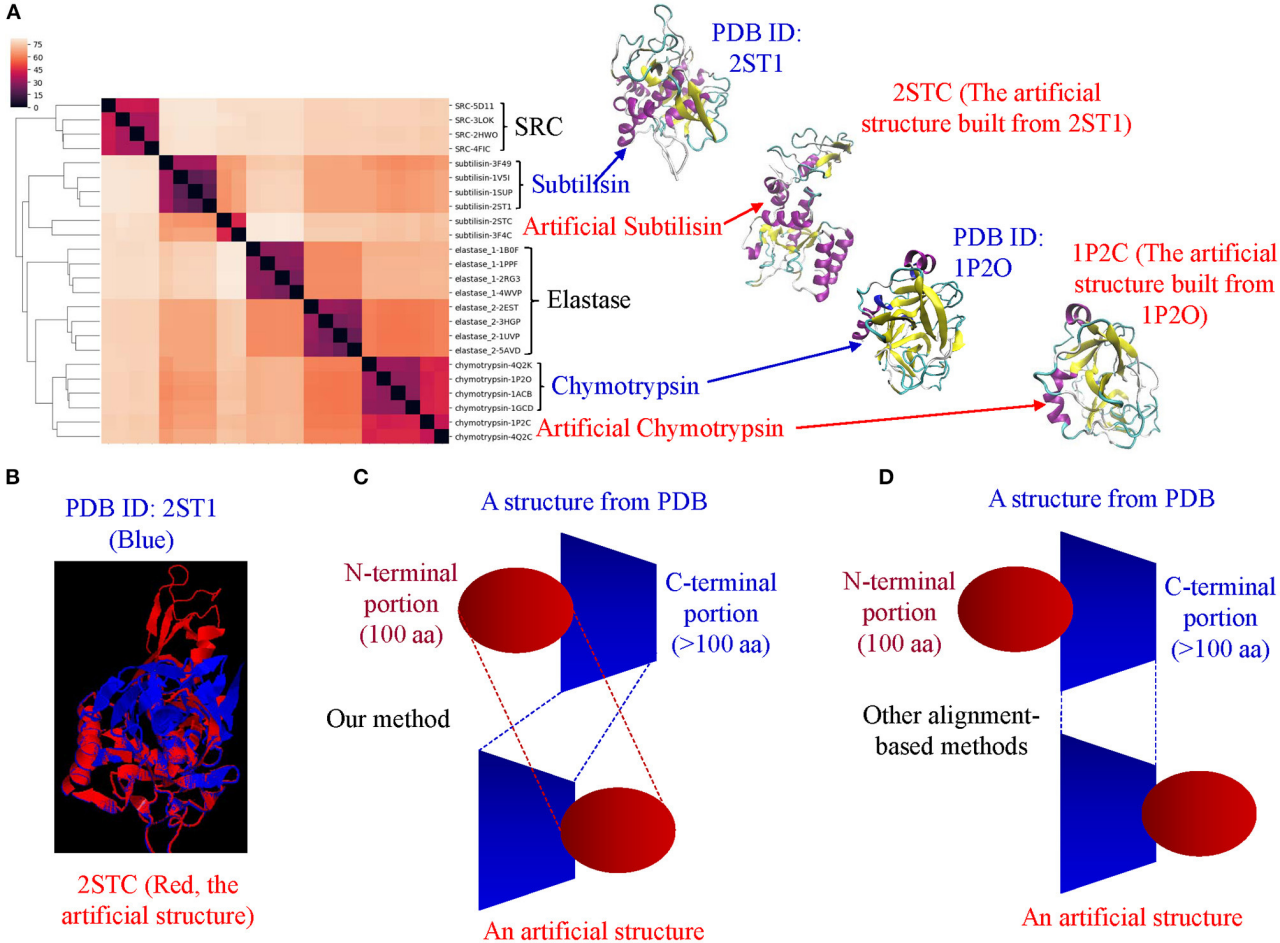
The clustering of the original and artificial structures demonstrates the difference of structural comparison methods. **(A)** The heatmap shows the clustering output of the original and artificial structures of SRC, subtilisin, elastase, and chymotrypsin. The artificial structures were generated by cleaving the peptide bond between 100th and 101th amino acids of the original structure and covalently rejoined the first 100 amino acids to the last amino acid. The representative original and artificial structures of subtilisin and chymotrypsin are shown; **(B)** The superimposed original and its artificial structures from TM-align method are shown; **(C,D)** The model shows the sensitivity of different methods to structural rearrangement. The dash lines indicate direct comparison of corresponding substructure.

One fundamental difference between our and other methods is that our method is relatively insensitive to substructure rearrangement of a protein. Other methods appear to be more sensitive to such type of structure rearrangement. To show this difference, we created artificial structures of chymotrypsin and subtilisin by rearranging the first 100 amino acids at the N-terminus to its original C-terminal end without changes of the first 100 amino acid sequence. Our method can distinguish the original and artificial structures of both chymotrypsin and subtilisin (Figure 15A). We were able to show the unique substructures belonging to the original structures, and also the specific substructures found only in the artificial structures (data not shown). Although the unique substructures are identified, our method is able to group the original and artificial structures together (Figure 15A). Interestingly, two originally separated elastase clusters are grouped together after we introduce structure diversity to chymotrypsin and subtilisin (Figure 15A). Other methods will successfully find corresponding amino acids of the same large portion between the original and artificial structures, but may have a challenge to identify the corresponding amino acids from the portion that was rearranged (Figure 15B). Figures 15C,D illustrates the fundamental difference between our and other alignment-based methods. In conclusion, each method has its advantages, and disadvantages. Our method can accurately cluster protein structures in a reasonably good way as other structural comparison methods. One important contribution is that we can provide in depth interpretation of our clustering results by thoroughly studying common and specific keys (substructures). Since our method has successfully shown that protein structure clusters match their functional classifications, our method depends more on substructures and less on protein sequences, and is relatively insensitive to structure rearrangement. These features represent a solid foundation that makes our method attractive for structure motif discovery.

### Our TSR-Based Method Has Its Unique Ways to Allow Structural Flexibility for Motif Discoveries or Protein Clustering

Most structure comparison methods consider protein folds as rigid bodies and quantify the structural similarity based on an average of atomic distances calculated using backbone coordinates. However, certain regions of a protein structure can be prone to variations, which arise due to structural flexibility for certain functions. In our approach, similar, but not identical, triangles could have identical keys due to the number of bin used in the key calculation. Additionally, we have been using key ± 1 for motif identification or discovery to allow structural flexibility. We can also adjust number of bin to meet the criteria to achieve certain desired structural flexibility. We present 12 combinations of bins from three MaxDist and four Theta for clustering and motif discovery. We recommend to use fewer bins from 12 candidates of bin combinations, e.g., Theta 7 and MaxDist 12, if the goal is to study structure topology and more bins, e.g., Theta 29 and MaxDist 35, for functional study or clustering of structurally similar proteins. One supporting evidence can be found in Supplementary Figure 29a where k-means algorithm clusters ERK1 and CDK8 structures that perfectly match with their functional classifications using Theta 29 and MaxDist 35. It agrees with the result using the hierarchical clustering method (Figure 5). We found one mismatch (ERK1-P-N) when Theta 7 and MaxDist 12 were used (Supplementary Figure 29b).

## Conclusions and Future Directions

### Conclusions

- We have developed a novel TSR-based structure comparison method where labels and shapes of triangles constructed with every three C_α_ atoms of a protein are formulated into integers called “keys.” 3-D structure of a protein is represented by a vector of integers.
- The method successfully clusters proteins that agree with their functional classifications in most cases. The clusters of protein structures can be further interpreted by different categories of keys.
- It can be used for structural motif identification and discovery.
- It can detect subtle conformational changes upon binding of a ligand or an interacting protein or due to post-translational modifications or point mutations. It provides a unique way to represent dynamic aspects of protein structures.
- It can distinguish alpha helices from beta pleated sheets and *vice versa*.
- Our method has its uniqueness compared with the popular methods.
- The new motifs or substructures we identified specifically for proteases and kinases provide a deeper insight into their structure and function relations.

We have provided PDB IDs and chain information for all the data sets in this study as Supplementary Files. The key files, and similarity/distance values will be available upon the request. If any specific keys are not described in detail, the information on amino acids and their positions will be made available as well upon the request. The source code is available for use of research purposes in github (https://github.com/SarikaKV/TSR-3D).

### Future Directions

To understand and make effective use of the vast structures in PDB, there is a need to develop tools for accurately and efficiently comparing protein structures and organizing proteins in rational fashion, e.g., sequence-, function-, and structure-based classifications. The challenges of aligning pairs of protein structures have attracted a significant level of research efforts. However, as yet, a good method which allows to search similar 3-D structures in a protein structure database for a given query of protein structure, does not exist; these can be thought of as the 3-D equivalent search of the 1-D sequence search using BLAST (Altschul et al., 1997) against protein sequence databases (e.g., NCBI). Our method makes it possible to systematically classify the structures available in PDB, to perform structure-BLAST search like BLAST search for amino acid sequences. We currently use only C_α_ atoms, a common practice. However, it involves loss of information with respect to geometries of side chains and structural relationships between side chains, and between side chains and main chains. In the future, we plan to incorporate side chain information into our current method for achieving more accurate protein structure classification, motif discovery, and for studying protein and drug, and protein and protein interactions.

## Data Availability Statement

The original contributions presented in the study are included in the article/Supplementary Materials, further inquiries can be directed to the corresponding author/s.

## Author Contributions

WX and VR initiated this project. WX designed this study and drafted the manuscript. WX, SK, and TS carried out structure-based protein clustering, motif identifications, discoveries, and prepared the figures. All authors read and revised the manuscript.

## Conflict of Interest

The authors declare that the research was conducted in the absence of any commercial or financial relationships that could be construed as a potential conflict of interest.

## References

[B1] Ackerman M. Ben-David S. (2016). A characterization of linkage-based hierarchical clustering. J. Mach. Learn. Res. 17, 8182–8198. Available online at: https://dl.acm.org/doi/10.5555/2946645.3053512

[B2] Altschul S. F. Madden T. L. Schaffer A. A. Zhang J. Zhang Z. Miller W.. (1997). Gapped BLAST and PSI-BLAST: a new generation of protein database search programs. Nucleic Acids Res. 25, 3389–3402. 10.1093/nar/25.17.3389 9254694 PMC146917

[B3] Alves N. A. Martinez A. S. (2007). Inferring topological features of proteins from amino acid residue networks. Phys. A Stat. Mech. Appl. 375, 336–344, 10.1016/j.physa.2006.09.014

[B4] Bartoli L. Fariselli P. Casadio R. (2008). The effect of backbone on the small-world properties of protein contact maps. Phys Biol. 4, L1–L5. 10.1088/1478-3975/4/4/L01 18185011

[B5] Batra J. Szabó A. Caulfield T. R. Soares A. S. Sahin-Tóth M. Radisky E. S. (2013). Long-range electrostatic complementarity governs substrate recognition by human chymotrypsin c, a key regulator of digestive enzyme activation. J. Biol. Chem. 288, 9848–9859. 10.1074/jbc.M113.457382 23430245 PMC3617285

[B6] Berman H. M. Bhat T. N. Bourne P. E. Feng Z. Gilliland G. Weissig H.. (2000). The protein data bank and the challenge of structural genomics. Nat. Struct. Biol. 7(Suppl.), 957–959. 10.1038/80734 11103999

[B7] Blow D. M. (1997). The tortuous story of Asp…His…Ser: structural analysis of α-chymotrypsin. Trends Biochem. Sci. 22, 405–408. 10.1016/S0968-0004(97)01115-8 9357317

[B8] Blundell T. Carney D. Gardner S. Hayes F. Howlin B. Hubbard T.. (1988). Knowledge-based protein modelling and design. Eur. J. Biochem. 172, 513–520. 10.1111/j.1432-1033.1988.tb13917.x 3280310

[B9] Bond J. S. (2019). Proteases: history, discovery, and roles in health and disease. J. Biol. Chem. 294, 1643–1651. 10.1074/jbc.TM118.004156 30710012 PMC6364759

[B10] Brenner S. E. Chothia C. Hubbard T. J. P. Murzin A. G. (1996). Understanding Protein structure: using scop for fold interpretation. Methods Enzymol. 266, 635–643. 10.1016/S0076-6879(96)66039-X 8743710

[B11] Bron C. Kerbosch J. (1973). Algorithm 457: finding all cliques of an undirected graph. Commun. ACM. 16, 575–577. 10.1145/362342.36236726169718

[B12] Carter P. Wells J. A. (1988). Dissecting the catalytic triad of a serine protease. Nature 332, 564–568. 10.1038/332564a0 3282170

[B13] Case D. A. Cheatham T. E. 3rd Darden T. Gohlke H. Luo R. Merz K. M. Jr.. (2005). The Amber biomolecular simulation programs. J. Comput. Chem. 26, 1668–1688. 10.1002/jcc.20290 16200636 PMC1989667

[B14] Cohen P. (2002). Protein kinases — the major drug targets of the twenty-first century? Nat. Rev. Drug Discovery. 1:309. 10.1038/nrd773 12120282

[B15] de Brevern G. Etchebest C. Hazout S. (2000). Bayesian probabilistic approach for predicting backbone structures in terms of protein blocks. Proteins 41, 271–287. 10.1002/1097-0134(20001115)41:3<271::AID-PROT10>3.0.CO;2-Z 11025540

[B16] Debret G. Martel A. Cuniasse P. (2009). RASMOT-3D PRO: a 3D motif search webserver. Nucleic Acids Res. 37, W459–W464. 10.1093/nar/gkp304 19417073 PMC2703991

[B17] Deininger M. Buchdunger E. Druker B. J. (2005). The development of imatinib as a therapeutic agent for chronic myeloid leukemia. Blood. 105, 2640–2653. 10.1182/blood-2004-08-3097 15618470

[B18] Ding C. H. Dubchak I. (2001). Multi-class protein fold recognition using support vector machines and neural networks. Bioinformatics 17, 349–358. 10.1093/bioinformatics/17.4.349 11301304

[B19] Dodson G. Wlodawer A. (1998). Catalytic triads and their relatives. Trends Biochem. Sci. 23, 347–352. 10.1016/S0968-0004(98)01254-7 9787641

[B20] Echalier A. Cot E. Camasses A. Hodimont E. Hoh F. Jay P.. (2012). An integrated chemical biology approach provides insight into Cdk2 functional redundancy and inhibitor sensitivity. Chem. Biol. 19, 1028–1040. 10.1016/j.chembiol.2012.06.015 22921070

[B21] Flaherty K. M. McKay D. B. Kabsch W. Holmes K. C. (1991). Similarity of the three-dimensional structures of actin and the ATPase fragment of a 70-kDa heat shock cognate protein. Proc. Natl. Acad. Sci. U.S. A. 88, 5041–5045. 10.1073/pnas.88.11.5041 1828889 PMC51803

[B22] Gallagher T. Oliver J. Bott R. Betzel C. Gilliland G. L. (1996). Subtilisin BPN' at 1.6 a resolution: analysis for discrete disorder and comparison of crystal forms. Acta Crystallogr. Sect. D. 52, 1125–1135. 10.1107/S0907444996007500 15299573

[B23] Gao X. Xie X.-J. Hsu F.-N. Li X. Liu M. Hemba-Waduge U.-S.. (2018). CDK8 mediates the dietary effects on developmental transition in Drosophila. Developmental Biology. 444, no. 2, 62-70. 10.1016/j.ydbio.2018.10.001 30352217 PMC6263851

[B24] Gibrat J. F. Madej T. Bryant S. H. (1996). Surprising similarities in structure comparison. Curr. Opin. Struct. Biol. 6, 377–385. 10.1016/S0959-440X(96)80058-3 8804824

[B25] Golovin A. Henrick K. (2008). MSDmotif: exploring protein sites and motifs. BMC Bioinform. 9:312. 10.1186/1471-2105-9-312 18637174 PMC2491636

[B26] Greene L. H. Lewis T. E. Addou S. Cuff A. Dallman T. Dibley M.. (2007). The CATH domain structure database: new protocols and classification levels give a more comprehensive resource for exploring evolution. Nucleic Acids Res. 35, (Suppl. 1), D291–D297. 10.1093/nar/gkl959 17135200 PMC1751535

[B27] Guru D. S. Nagabhushan P. (2001). Triangular spatial relationship: a new approach for spatial knowledge representation. Pattern Recogn. Lett. 22, 999–1006, 10.1016/S0167-8655(01)00043-5

[B28] Hasegawa H. Holm L. (2009). Advances and pitfalls of protein structural alignment. Curr. Opin. Struct. Biol. 19, 341–348. 10.1016/j.sbi.2009.04.003 19481444

[B29] Holm L. (2019). Benchmarking fold detection by DaliLite v.5. Bioinformatics 35, 5326–5327. 10.1093/bioinformatics/btz536 31263867

[B30] Holm L. Sander C. (1993). Protein structure comparison by alignment of distance matrices. J. Mol. Biol. 233, 123–138. 10.1006/jmbi.1993.1489 8377180

[B31] Holm L. Sander C. (1996). The FSSP database: fold classification based on structure-structure alignment of proteins. Nucleic Acids Res. 24, 206–209. 10.1093/nar/24.1.206 8594580 PMC145583

[B32] Homeyer N. Horn A. H. C. Lanig H. Sticht H. (2006). AMBER force-field parameters for phosphorylated amino acids in different protonation states: phosphoserine, phosphothreonine, phosphotyrosine, and phosphohistidine. J. Mol. Model. 12, 281–289. 10.1007/s00894-005-0028-4 16240095

[B33] Hou J. Jun S.-R. Zhang C. Kim S.-H. (2005). Global mapping of the protein structure space and application in structure-based inference of protein function. Proc. Natl. Acad. Sci. U.S. A. 102, 3651–3656. 10.1073/pnas.0409772102 15705717 PMC548596

[B34] Hubert L. Arabie P. (1985). Comparing partitions. J. Classification 2, 193–218. 10.1007/BF01908075

[B35] Humphrey W. Dalke A. Schulten K. (1996). VMD: Visual molecular dynamics. J. Mol. Graph. 14, 33–38. 10.1016/0263-7855(96)00018-5 8744570

[B36] Hunter T. (2000). Signaling−2000 and beyond. Cell. 100, 113–127. 10.1016/S0092-8674(00)81688-8 10647936

[B37] Jaccard P. (1901). Etude comparative de la distribution florale dans une portion des Alpes et des Jura. Bull. Soc. Vaudoise Sci. Nat. 37, 547–579.

[B38] Jolliffe I. T. (1986). Principal Component Analysis. New York, NY: Springer. 10.1007/978-1-4757-1904-8

[B39] Joseph A. P. Agarwal G. Mahajan S. Gelly J. C. Swapna L. S. Offmann B.. (2010). A short survey on protein blocks. Biophys. Rev. 2, 137–147. 10.1007/s12551-010-0036-1 21731588 PMC3124139

[B40] Kabsch W. Sander C. (1983). Dictionary of protein secondary structure: pattern recognition of hydrogen-bonded and geometrical features. Biopolymers 22, 2577–2637. 10.1002/bip.360221211 6667333

[B41] Karchin R. Cline M. Karplus K. (2004). Evaluation of local structure alphabets based on residue burial. Proteins 55, 508–518. 10.1002/prot.20008 15103615

[B42] Kinoshita K. Nakamura H. (2003). Identification of protein biochemical functions by similarity search using the molecular surface database eF-site. Protein Sci. 12, 1589–1595. 10.1110/ps.0368703 12876308 PMC2323945

[B43] Kinoshita T. Yoshida I. Nakae S. Okita K. Gouda M. Matsubara M.. (2008). Crystal structure of human mono-phosphorylated ERK1 at Tyr204. Biochem. Biophys. Res. Commun. 377, 1123–1127. 10.1016/j.bbrc.2008.10.127 18983981

[B44] Kleywegt G. J. (1999). Recognition of spatial motifs in protein structures. J. Mol. Biol. 285, 1887–1897. 10.1006/jmbi.1998.2393 9917419

[B45] Kolodny R. Koehl P. Levitt M. (2005). Comprehensive evaluation of protein structure alignment methods: scoring by geometric measures. J. Mol. Biol. 346, 1173–1188. 10.1016/j.jmb.2004.12.032 15701525 PMC2692023

[B46] Konagurthu A. S. Lesk A. M. Allison L. (2012). Minimum message length inference of secondary structure from protein coordinate data. Bioinformatics 28, i97–i105. 10.1093/bioinformatics/bts223 22689785 PMC3371855

[B47] Konno S. Namiki T. Ishimori K. (2019). Quantitative description and classification of protein structures by a novel robust amino acid network: interaction selective network (ISN). Sci. Rep. 9:16654 10.1038/s41598-019-52766-6

[B48] Krissinel E. Henrick K. (2004). Secondary-structure matching (SSM), a new tool for fast protein structure alignment in three dimensions. Acta Crystallogr. D Biol. Crystallogr. 60, 12(Pt 1), 2256–2268. 10.1107/S0907444904026460 15572779

[B49] Kruskal J. B. Wish M. (1978). Multidimensional scaling. SAGE Publications, Inc. 10.4135/9781412985130

[B50] Kumar S. Stecher G. Tamura K. (2016). MEGA7: molecular evolutionary genetics analysis version 7.0 for bigger datasets. Mol. Biol. Evol. 33, 1870–1874. 10.1093/molbev/msw054 27004904 PMC8210823

[B51] Lackner P. Koppensteiner W. A. Sippl M. J. Domingues F. S. (2000). ProSup: a refined tool for protein structure alignment. Protein Eng. 13, 745–752. 10.1093/protein/13.11.745 11161105

[B52] Lesk A. Hardman K. (1982). Computer-generated schematic diagrams of protein structures. Science 216, 539–540. 10.1126/science.7071602 7071602

[B53] Lewis T. S. Shapiro P. S. Ahn N. G. (1998). Signal transduction through MAP kinase cascades, in Advances in Cancer Research, eds vande Woude G. F. Klein G. (Cambridge, MA: Academic Press), 49–139. 10.1016/S0065-230X(08)60765-4 9561267

[B54] Liu H. Hussain F. Tan L. C. Dash M. (2002). Discretization: an enabling technique. Data Mining Knowledge Discov. 6, 393–423. 10.1023/A:1016304305535

[B55] Lloyd S. (1982). Least squares quantization in PCM. IEEE Trans. Inform. Theory. 28, 129–137. 10.1109/TIT.1982.1056489

[B56] Lo Conte L. Ailey B. Hubbard T. J. P. Brenner S. E. Murzin A. G. Chothia C. (2000). SCOP: a structural classification of proteins database. Nucleic Acids Res. 28, 257–259. 10592240 10.1093/nar/28.1.257 PMC102479

[B57] López-Otín C. Bond J. S. (2008). Proteases: multifunctional enzymes in life and disease. J. Biol. Chem. 283, 45, 30433–30437. 10.1074/jbc.R800035200 18650443 PMC2576539

[B58] Lu G. Moriyama E. N. (2004). Vector NTI, a balanced all-in-one sequence analysis suite. Brief. Bioinformatics. 5, 378–388. 10.1093/bib/5.4.378 15606974

[B59] Madej T. Gibrat J.-F. Bryant S. H. (1995). Threading a database of protein cores. Proteins 23, 356–369. 10.1002/prot.340230309 8710828

[B60] Mayr G. Domingues F. S. Lackner P. (2007). Comparative analysis of protein structure alignments. BMC Struct. Biol. 7:50. 10.1186/1472-6807-7-50 17672887 PMC1959231

[B61] Murzin A. G. Brenner S. E. Hubbard T. Chothia C. (1995). SCOP: a structural classification of proteins database for the investigation of sequences and structures. J. Mol. Biol. 247, 536–540. 10.1016/S0022-2836(05)80134-2 7723011

[B62] Nadzirin N. Gardiner E. J. Willett P. Artymiuk P. J. Firdaus-Raih M. (2012). SPRITE and ASSAM: web servers for side chain 3D-motif searching in protein structures. Nucleic Acids Res. 40, 380–W386. 10.1093/nar/gks401 22573174 PMC3394286

[B63] Nadzirin N. Willett P. Artymiuk P. J. Firdaus-Raih M. (2013). IMAAAGINE: a webserver for searching hypothetical 3D amino acid side chain arrangements in the protein data bank. Nucleic Acids Res. 41, W432–W440. 10.1093/nar/gkt431 23716645 PMC3692123

[B64] Nussinov R. Wolfson H. J. (1991). Efficient detection of three-dimensional structural motifs in biological macromolecules by computer vision techniques. Proc. Natl. Acad. Sci. 88, 10495–10499. 10.1073/pnas.88.23.10495 1961713 PMC52955

[B65] Odoux A. Jindal D. Tamas T. C. Lim B. W. H. Pollard D. Xu W. (2016). Experimental and molecular dynamics studies showed that CBP KIX mutation affects the stability of CBP:c-Myb complex. Comput. Biol. Chem. 62, 47–59, 10.1016/j.compbiolchem.2016.03.004 27082784

[B66] Offmann B. Tyagi M. de Brevern G. A. (2007). Local protein structures. Curr. Bioinform. 2, 165–202. 10.2174/15748930778166210521731588

[B67] Orengo C. A. Michie A. D. Jones S. Jones D. T. Swindells M. B. Thornton J. M. (1997). CATH–a hierarchic classification of protein domain structures. Structure 5, 1093–1109. 10.1016/S0969-2126(97)00260-8 9309224

[B68] Orengo C. A. Taylor W. R. (1996). SSAP: sequential structure alignment program for protein structure comparison. Meth. Enzymol. 266, 617–635. 10.1016/S0076-6879(96)66038-8 8743709

[B69] Orengo C. A. Todd A. E. Thornton J. M. (1999). From protein structure to function. Curr. Opin. Struct. Biol. 9, 374–382. 10.1016/S0959-440X(99)80051-7 10361094

[B70] Pandini A. Fornili A. Kleinjung J. (2010). Structural alphabets derived from attractors in conformational space. BMC Bioinformatics. 11:97. 10.1186/1471-2105-11-97 20170534 PMC2838871

[B71] Pauling L. Corey R. B. Branson H. R. (1951). The structure of proteins: two hydrogen-bonded helical configurations of the polypeptide chain. Proc. Natl. Acad. Sci. 37, 205–211. 10.1073/pnas.37.4.205 14816373 PMC1063337

[B72] Perutz M. F. Rossmann M. G. Cullis A. F. Muirhead H. Will G. North A. C. T. (1960). Structure of haemoglobin: a three-dimensional fourier synthesis at 5.5-[angst]. resolution, obtained by X-ray analysis. Nature 185, 416–422. 10.1038/185416a0 18990801

[B73] Poss Z. C. Ebmeier C. C. Taatjes D. J. (2013). The mediator complex and transcription regulation. Crit. Rev. Biochem. Mol. Biol. 48, 575–608. 10.3109/10409238.2013.840259 24088064 PMC3852498

[B74] Rawlings N. D. Barrett A. J. (1993). Evolutionary families of peptidases. Biochem. J. 290, 205–218. 10.1042/bj2900205 8439290 PMC1132403

[B75] Rawlings N. D. Barrett A. J. (1994). Families of serine peptidases, “Methods in Enzymology, 19-61: Academic Press, 10.1016/0076-6879(94)44004-2 PMC7133253 7845208

[B76] Remington S. J. Matthews B. W. (1980). A systematic approach to the comparison of protein structures. J. Mol. Biol. 140, 77–99. 10.1016/0022-2836(80)90357-5 6774102

[B77] Roe D. R. Cheatham T. E. (2013). PTRAJ and CPPTRAJ: software for processing and analysis of molecular dynamics trajectory data. J. Chem. Theor. Comput. 9, 3084–3095. 10.1021/ct400341p 26583988

[B78] Rossmann M. G. Argos P. (1975). A comparison of the heme binding pocket in globins and cytochrome b5. J. Biol. Chem. 250, 7525–7532. 1165251

[B79] Rossmann M. G. Argos P. (1976). Exploring structural homology of proteins. J. Mol. Biol. 105, 75–95. 10.1016/0022-2836(76)90195-9 186608

[B80] Russell R. B. Barton G. J. (1992). Multiple protein sequence alignment from tertiary structure comparison: assignment of global and residue confidence levels. Proteins 14, 309–323. 10.1002/prot.340140216 1409577

[B81] Šali A. Blundell TL (1993). Comparative protein modelling by satisfaction of spatial restraints. J. Mol. Biol. 234, 779–815. 10.1006/jmbi.1993.1626 8254673

[B82] Salvesen G. S. Hempel A. Coll N. S. (2016). Protease signaling in animal and plant-regulated cell death. FEBS J. 283, 2577–2598. 10.1111/febs.13616 26648190 PMC5606204

[B83] Schneider E. V. Böttcher J. Blaesse M. Neumann L. Huber R. Maskos K. (2011). The Structure of CDK8/CycC implicates specificity in the CDK/cyclin family and reveals interaction with a deep pocket binder. J. Mol. Biol. 412, 251–266. 10.1016/j.jmb.2011.07.020 21806996

[B84] Seeliger M. A. Nagar B. Frank F. Cao X. Henderson M. N. Kuriyan J. (2007). c-Src binds to the cancer drug imatinib with an inactive Abl/c-Kit conformation and a distributed thermodynamic penalty. Structure 15, 299–311. 10.1016/j.str.2007.01.015 17355866

[B85] Shapiro J. Brutlag D. (2004a). FoldMiner and LOCK 2: protein structure comparison and motif discovery on the web. Nucleic Acids Res. 32, W536–W541. 10.1093/nar/gkh389 15215444 PMC441527

[B86] Shapiro J. Brutlag D. (2004b). FoldMiner: structural motif discovery using an improved superposition algorithm. Protein Sci. 13, 278–294. 10.1110/ps.03239404 14691242 PMC2286532

[B87] Shen H.-B. Chou K.-C. (2006). Ensemble classifier for protein fold pattern recognition. Bioinformatics 22, 1717–1722. 10.1093/bioinformatics/btl170 16672258

[B88] Shindyalov I. N. Bourne P. E. (1998). Protein structure alignment by incremental combinatorial extension (CE) of the optimal path. Protein Eng. 11, 739–747, 10.1093/protein/11.9.739 9796821

[B89] Sillitoe I. Lewis T. E. Cuff A. Das S. Ashford P. Dawson N. L.. (2015). CATH: comprehensive structural and functional annotations for genome sequences. Nucleic Acids Res. 43, D376–D381. 10.1093/nar/gku947 25348408 PMC4384018

[B90] Simmerling C. Strockbine B. Roitberg A. E. (2002). All-atom structure prediction and folding simulations of a stable protein. J. Am. Chem. Soc. 124, 11258–11259. 10.1021/ja0273851 12236726

[B91] Singh A. P. Brutlag D. L. (1997). Hierarchical protein structure superposition using both secondary structure and atomic representations. Proc. Int. Conf. Intell. Syst. Mol. Biol. 5, 284–293. 9322051

[B92] Steinbrecher T. Latzer J. Case D. A. (2012). Revised AMBER parameters for bioorganic phosphates. J. Chem. Theory Comput. 8, 4405–4412. 10.1021/ct300613v 23264757 PMC3524595

[B93] Szustakowski J. D. Weng Z. (2000). Protein structure alignment using a genetic algorithm. Proteins 38, 428–440. 10.1002/(SICI)1097-0134(20000301)38:4<;428::AID-PROT8>;3.0.CO;2-N 10707029

[B94] Taylor W. R. Orengo C. A. (1989). Protein structure alignment. Journal of Molecular Biology. 208, 1–22. 10.1016/0022-2836(89)90084-3 2769748

[B95] Tonks N. K. (2006). Protein tyrosine phosphatases: from genes, to function, to disease. Nat. Rev. Mol. Cell Biol. 7:833. 10.1038/nrm2039 17057753

[B96] Tseng Y. Y. Li W.-H. (2012). Classification of protein functional surfaces using structural characteristics. Proc. Natl. Acad. Sci. 109, 1170–1175. 10.1073/pnas.1119684109 22238424 PMC3268291

[B97] Ullmann J. R. (1976). An algorithm for subgraph isomorphism. J. ACM. 23, 31–42, 10.1145/321921.321925

[B98] Unger R. Harel D. Wherland S. Sussman J. L. (1989). A 3D building blocks approach to analyzing and predicting structure of proteins. Proteins 5, 355–373. 10.1002/prot.340050410 2798411

[B99] Vetrivel I. Mahajan S. Tyagi M. Hoffmann L. Sanejouand Y.-H. Srinivasan N.. (2017). Knowledge-based prediction of protein backbone conformation using a structural alphabet. PLoS ONE 12:e0186215. 10.1371/journal.pone.0186215 29161266 PMC5697859

[B100] Witten I. H. Frank E. Hall M. A. Pal C. J. (2016). Data Mining: Practical Machine Learning Tools and Techniques. Amsterdam: Elsevier Science.

[B101] Wohlers I. Malod-Dognin N. Andonov R. Klau G. W. (2012). CSA: comprehensive comparison of pairwise protein structure alignments. Nucleic Acids Res. 40, W303–W309. 10.1093/nar/gks362 22553365 PMC3394275

[B102] Xie X.-J. Hsu F.-N. Gao X. Xu W. Ni J.-Q. Xing Y.. (2015). CDK8-cyclin C mediates nutritional regulation of developmental transitions through the ecdysone receptor in Drosophila. PLoS Biol. 13:e1002207. 10.1371/journal.pbio.1002207 26222308 PMC4519132

[B103] Xu J. Zhang Y. (2010). How significant is a protein structure similarity with TM-score = 0.5? Bioinformatics 26, 889–895. 10.1093/bioinformatics/btq066 20164152 PMC2913670

[B104] Xu W. Amire-Brahimi B. Xie X.-J. Huang L. Ji J.-Y. (2014). All-atomic molecular dynamic studies of human CDK8: insight into the A-loop, point mutations and binding with its partner CycC. Comput. Biol. Chem. 51, 1–11. 10.1016/j.compbiolchem.2014.03.003 24754906 PMC4122639

[B105] Xu W. Ji J. Y. (2011). Dysregulation of CDK8 and cyclin C in tumorigenesis. J. Genet. Genomics. 38, 439–452. 10.1016/j.jgg.2011.09.002 22035865 PMC9792140

[B106] Xu W. Zhang Y. Achi O. Y. Knierim K. D. Hanks J. G. Wang Y. (2019). Tyrosine nitration of human ERK1 introduces an intra-hydrogen bond by molecular dynamics simulations. Struct. Chem. 30, 1459–1470. 10.1007/s11224-019-01306-z

[B107] Yang A. S. Honig B. (2000). An integrated approach to the analysis and modeling of protein sequences and structures. I. Protein structural alignment and a quantitative measure for protein structural distance. J. Mol. Biol. 301, 665–678. 10.1006/jmbi.2000.3973 10966776

[B108] Yeung K. Y. Ruzzo W. L. (2001). Principal component analysis for clustering gene expression data. Bioinformatics 17, 763–774. 10.1093/bioinformatics/17.9.763 11590094

[B109] Zemla A. (2003). LGA: a method for finding 3D similarities in protein structures. Nucleic Acids Res. 31, 3370–3374. 10.1093/nar/gkg571 12824330 PMC168977

[B110] Zhang Y. Huang X. Wang J. Wang X. Liu X. Chen Y.. (2019). Nitration-induced ubiquitination and degradation control quality of ERK1. Biochem. J. 476, 1911–1926. 10.1042/BCJ20190240 31196894 PMC6604951

[B111] Zhang Y. Skolnick J. (2005). TM-align: a protein structure alignment algorithm based on the TM-score. Nucleic Acids Res. 33, 2302–2309. 10.1093/nar/gki524 15849316 PMC1084323

