# Supplementary material for: Development of a TSR-Based Method for Protein 3-D Structural Comparison With Its Applications to Protein Classification and Motif Discovery
Source: Front Chem. 2021 Jan 13;8:602291. doi: 10.3389/fchem.2020.602291 (PMC7838567; doi:10.3389/fchem.2020.602291)
Supplement: Supplementary file 1 [file Table_1.DOCX]

**The list of the Supplementary Files**

Supplementary_File1_Protease_Dataset

Supplementary_File1A_Protease_Clustering_List

Supplementary_File2_MD_ERK1_CDK8

Supplementary_File3_CDK2

Supplementary_File4_CATH

Supplementary_File5_SCOP

Supplementary_File6_DD

Supplementary_File7_101

Supplementary_File8_S10

Supplementary_File8A_S10_List

Supplementary_File9_CDK2_4EOQ_12Keys

Supplementary_File10_Kinase_Phosphatase

Supplementary_File11_3GVU_BCR_ABL_12Keys

Supplementary_File12_SCOP_40

Supplementary_File13_SCOP_40_95

Supplementary_File14_101_Distance
