# Supplementary material for: Development of a TSR-Based Method for Protein 3-D Structural Comparison With Its Applications to Protein Classification and Motif Discovery
Source: Front Chem. 2021 Jan 13;8:602291. doi: 10.3389/fchem.2020.602291 (PMC7838567; doi:10.3389/fchem.2020.602291)

Figure Legend for Supplementary Figures

Figure S1. The flow chart of this study. Vectorization, Application/Evaluation and Visualization are highlighted in blue. Different shapes represent the main modules of Vectorization (Isosceles trapezoid with the shorter parallel edge down) and Application/Evaluation (Isosceles trapezoid with the shorter parallel edge up), structure data sets (Parallelogram), algorithms (Rounded rectangle) and visualization (Trapezoid-like with a curve as an edge). GJC: Generalized Jaccard coefficient.

Figure S2. Distributions of Theta of 12 protein samples randomly selected from PDB.

Figure S3. Distributions of MaxDist of 12 protein samples randomly selected from PDB. Top five numbers of bins for MaxDist with the smallest variances are indicated.

Figure S4. Variances of numbers of bins of Theta of 12 protein samples randomly selected from PDB. Top five numbers of bins for Theta with the smallest variances are indicated.

Figure S5. Variances of numbers of bins of MaxDist of 12 protein samples randomly selected from PDB. Top five numbers of bins for MaxDist with the smallest variances are indicated.

Figure S6. Determination of numbers of bins for Theta and MaxDist. a, Top two numbers of bins selected from top five numbers of bins with the smallest variances for each sample (Samples 1-12) based on the calculations from Theta, MaxDist, all three angles or all three edge lengths; b, The minimum, median and maximum of numbers of bins for Theta and MaxDist were calculated

from the top two numbers of bins. The numbers of bins with the highest frequency for samples 1-12 are shown; c, The top three numbers of bins for MaxDist were chosen mainly based on analyses from a, and b; d, The top four numbers of bins for Theta were chosen mainly based on the analyses from a, and b.

Figure S7. Comparison of protein 3-D structure-based clustering with sequence-based classification. a-b, 16 proteins were randomly selected from four groups: CBP, STAT, Kinase and Protease, and clustered by structural comparison (a) and classified by sequence alignment (b); c-d, 16 proteins were randomly selected from four groups: hemoglobin, cyclin, adenylyl cyclase and CREB, and clustered by structural comparison (c) and classified by sequence alignment (d). a-d, PDB IDs are indicated.

Figure S8. Comparison of protein 3-D structure-based clustering with sequence-based classification. a-b, 24 proteins were randomly selected from four groups: glucose transporter, heat shock protein, actin and immunoglobulin, and clustered by structural comparison (a) and classified by sequence alignment (b); c-d, 24 proteins were randomly selected from four groups: RNase, reaction center, transferase and MHC, and clustered by structural comparison (c) and classified by sequence alignment (d). a-d, PDB IDs are indicated.

Figure S9. Comparison of protein 3-D structure-based clustering with sequence-based classification. a-b, 24 proteins were randomly selected from four groups: glycerol dehydratase, cyclin-dependent kinase, triose phosphatase isomer, and restriction enzyme, and clustered by structural comparison (a) and classified by sequence alignment (b). a-b, PDB IDs are indicated.

Figure S10. Comparison of protein 3-D structure-based clustering with sequence-based classification. a-b, 24 proteins were randomly selected from four groups: retinoblastoma, Ras, epidermal growth factor receptor, and G protein coupled receptor, and clustered by structural comparison (a) and classified by sequence alignment (b). a-b, PDB IDs are indicated.

Figure S11. Protein 3-D structure-based clustering of 178 proteins selected from 6 functional classes. 178 proteins were selected from six groups: peptidase, fibroblast growth factor 1 (FGF1), factor X, fructose 1,6-bisphosphatase (F16B), vitamin D3 receptor (D3R) and nuclear receptor coactivator 2 (NRCO2). We selected the proteins with similar amino acid numbers for each group. Some of the PDB IDs are indicated in the clustering map. All PDB IDs, chain and class information in this data set are provided in Supplementary File 8. The complete list of the clustering result in the same order as the clustering map are provided in Supplementary File 8A.

Figure S12. Numbers of the Total, Total Different, Total Common and Total Different Common keys and differences in Theta, MaxDist and frequency between the Total, Common and Uncommon keys of the serine proteases. a, Number of the Total, Total Different, Total Common and Total Different Common keys of the serine proteases; b, Differences in Theta between the Total, Common and Uncommon keys of the serine proteases; c, Differences in MaxDist between the Total, Common and Uncommon keys of the serine proteases; d, Differences in frequency between the Total, Common and Uncommon keys of the serine proteases. a-d, Numbers of the proteins in each subclass of the serine proteases are indicated.

Figure S13. The sequence alignment, and specific keys and their corresponding structure of subclasses of serine proteases. a, The sequence alignment of the representative sequences selected from serine protease subclasses; b-e, The specific keys for the serine protease subclasses: prothrombin (b), plasmin (c), choline esterase (d), acetylcholine esterase (e). Protein numbers for the subclasses are indicated; f, A representative structure (PDB ID: 4H4F) shows hydrogen bonds between two keys or within the keys.

Figure S14. The studies using MDS, calculations of Venn diagram, similarity distribution, and common keys, and PCA reveal that our method cannot distinguish classes of alpha, beta and alpha-beta. a, MDS analysis using the structures from three classes of CATH database that contain 151 to 200 amino acids in the secondary structures using Theta 29 and MaxDist 35. The PDB IDs and chain information can be found in Supplementary File 4; b, The structures studied in a, are investigated in b, using Theta 7 and MaxDist 12; c-e, The same structures studied in a and b, are also used in the studying effect of amino acid grouping on clustering. For the case with amino acid grouping, we have used three schemas named as aa1 (c), aa2 (d) and aa3 (e). For aa1, there are 13 groups and they are (Ser, Thr), (Ala, Val), (Leu, Ile), (Phe, Trp), (Asp, Glu), (Asn, Gln), (Lys, Arg), (Gly), (Pro), (Cys), (Met), (Trp), and (His). For aa2, there are 7 groups: aliphatic (Gly, Ala, Val, Leu, Ile, Pro), aromatic (Phe, Tyr, Trp), OH-containing (Ser, Thr), Sulfur-containing (Cys, Met), Positively charged (His, Lys, Arg), Negatively charge (Asp, Glu) and Amide-containing (Asn, Gln). For aa3, all 20 amino acids are in the same group. From aa1 to aa3, each group is assigned a unique integer; f-g, The Venn diagrams using Theta 29/MaxDist 35 (f) and Theta 7/MaxDist 12 (g) show counts of the keys that are specific to each class, and all possibly overlapped regions among CATH classes. The numbers of total distinct keys, the specific keys for

α, β, and αβ, the keys in all three-class overlapped region are indicated; h, The pairwise similarities of the structures in a-g were calculated and similarity distribution is plotted. The weighted percent structure similarities were calculated and are shown for Theta 29/MaxDist 35, Theta 7/MaxDist 12, aa1, aa2, and aa3; i, The numbers of common keys for each protein in the CATH data set were calculated and are presented; j, The Variance vs PCA plot shows the effect of varying the numbers of bins and applying aa grouping.

Figure S15. The studies using MDS, and calculations of Venn diagram reveal that our method cannot distinguish classes of alpha, beta and alpha-beta after feature selection is applied. a-c, MDS analysis using the structures from three classes of CATH database that contain 151 to 200 amino acids in the secondary structures using Theta 29 and MaxDist 35 without (a) and with feature selections (b, IESS; c, IASS); d-f, The Venn diagrams show counts of the keys that are specific to each class, and all possibly overlapped regions among CATH classes without (d) and with feature selections (e, IESS; f, IASS). The numbers of total distinct keys, the specific keys for α, β, and αβ, and the keys in all three-class overlapped region are indicated.

Figure S16. The k-means clustering was performed using the structures with low and high sequence similarities from SCOP database and the clustering results show on obvious effect of sequence similarity on differentiation of two main types of secondary structures. a-c, The structures with low pairwise amino acid sequence similarity (< 40%) were investigated by k-means clustering method without (a) and with feature selections (b, IESS; c, IASS). The PDB IDs, chain and class information can be found in Supplementary File 12; d-f, The structures with high pairwise amino acid sequence similarity (40% - 95%) were investigated by k-means clustering

method without (d) and with feature selections (e, IESS; f, IASS). The PDB IDs, chain and class information can be found in Supplementary File 13. a-f, The numbers of the mismatched structures and Adjusted Rand Index (ARI) are indicated.

Figure S17. The PCA was performed using the structures with low and high sequence similarities from SCOP database and the results show on obvious effect of sequence similarity on differentiation of two main types of secondary structures. a-c, The structures with low pairwise amino acid sequence similarity (< 40%) were investigated by PCA without (a) and with feature selections (b, IESS; c, IASS). The PDB IDs, chain and class information can be found in Supplementary File 12; d-f, The structures with high pairwise amino acid sequence similarity (40% - 95%) were investigated by PCA without (d) and with feature selections (e, IESS; f, IASS). The PDB IDs, chain and class information can be found in Supplementary File 13.

Figure S18. The studies using MDS, and calculations of Venn diagram reveal that our method can distinguish classes of alpha and beta after feature selections are applied. a-d, MDS analysis using the structures from alpha and beta classes selected from literature, DD data set, without (a) and with feature selections (b, IESS; c-d, IASS). d, The structures containing less than 50 and more than 200 amino acids in either alpha helices or beta pleated sheet were removed from c; e-g, The Venn diagrams show counts of the keys that are specific to each class, and are in the intersection of alpha and beta without (e) and with feature selections (f, IESS; g-h, IASS). h, The structures containing less than 50 and more than 200 amino acids in either alpha helices or beta pleated sheet were removed from g. The numbers of total distinct keys, the specific keys for α and β, and the keys in the intersection are indicated.

Figure S19. Clustering, and key numbers and properties of ArsC and Prdx2 proteins. a, The sequence alignment of ArsC and Prdx2 was performed. Amino acid similarity and identity are 5.8% and 89.8% respectively; b, Clustering of ArsC and Prdx2; c, The Venn diagram shows the numbers of the specific keys and overlapping keys for ArsC and Prdx2; d, Numbers of the Total, Total Different, Total Common and Total Different Common keys were calculated; e, Theta and MaxDist of all, Common and Uncommon keys were calculated.

Figure S20. Clustering, and key numbers and properties of Hsp70 and Actin proteins. a, The Venn diagram illustrates the numbers of the specific keys and overlapping keys for Hsp70 and Actin; b, Numbers of the Total, Total different, Total Common and Total Different Common keys were calculated; c, Clustering of Hsp70 and Actin; d, The sequence alignment of Hsp70 and Actin was performed. Amino acid similarity and identity are 5.3% and 89.2% respectively; e, Theta and MaxDist of all, Common and Uncommon keys were calculated.

Figure S21. Representation of Arsc, Prdx2, Hsp70 and Actin clusters by Multidimensional Scaling method. Numbers of the distinct and specific keys are indicated.

Figure S22. The dendrogram generated by DALI server shows the clustering of the proteases, kinases and phosphatases. Proteins subclasses based on their functional classifications are labelled.

Figure S23. The dendrogram using RMSD generated by CE method shows the clustering of

the proteases, kinases and phosphatases. Proteins subclasses based on their functional classifications are labelled.

Figure S24. The dendrogram using RMSD generated by TM-align method shows the clustering of the proteases, kinases and phosphatases. Proteins subclasses based on their functional classifications are labelled.

Figure S25. The dendrogram generated by our method shows the clustering of the proteases, kinases and phosphatases. Proteins subclasses based on their functional classifications are labelled.

Figure S26. The phylogenetic analysis of the proteases, kinases and phosphatases using MEGA 7.0.

Figure S27. The PCA of the proteases, kinases and phosphatases. a, The PCA vs Variance plot shows the contributions of first 10 principle components of different methods to the variances; b, The diagram shows the separation of the clusters using the first three principle components from the RMSD matrix generated by TM-align method; c, The diagram shows the separation of the clusters using the first three principle components from the genetic distance matrix generated by Clustal W and Neighbor-Joining algorithms.

Figure S28. A small set of specific distinct keys were identified for SRC (219), Tyr phosphatase (3), chymotrypsin (11), subtilisin (65), CDK (4,297), elastase (0), Ser/Thr

phosphatase (948), trypsin (45), prothrombin (44) and plasmin (34). A total number of the distinct keys for this data set is shown.

Figure S29. The k-means clustering was performed using the ERK1 and CDK8 structures and the clustering results show more bins for Theta and MaxDist have an advantage over less bins to distinguish structurally similar proteins. a, Theta 29 and MaxDist 35 were used in the clustering study of the ERK1 and CDK8 structures; b, Theta 7 and MaxDist 12 were used in the clustering study of the ERK1 and CDK8 structures. The group having a mismatched structure is highlighted in red. a-b, The number of the mismatched structure and ARI are indicated.

Figure S1

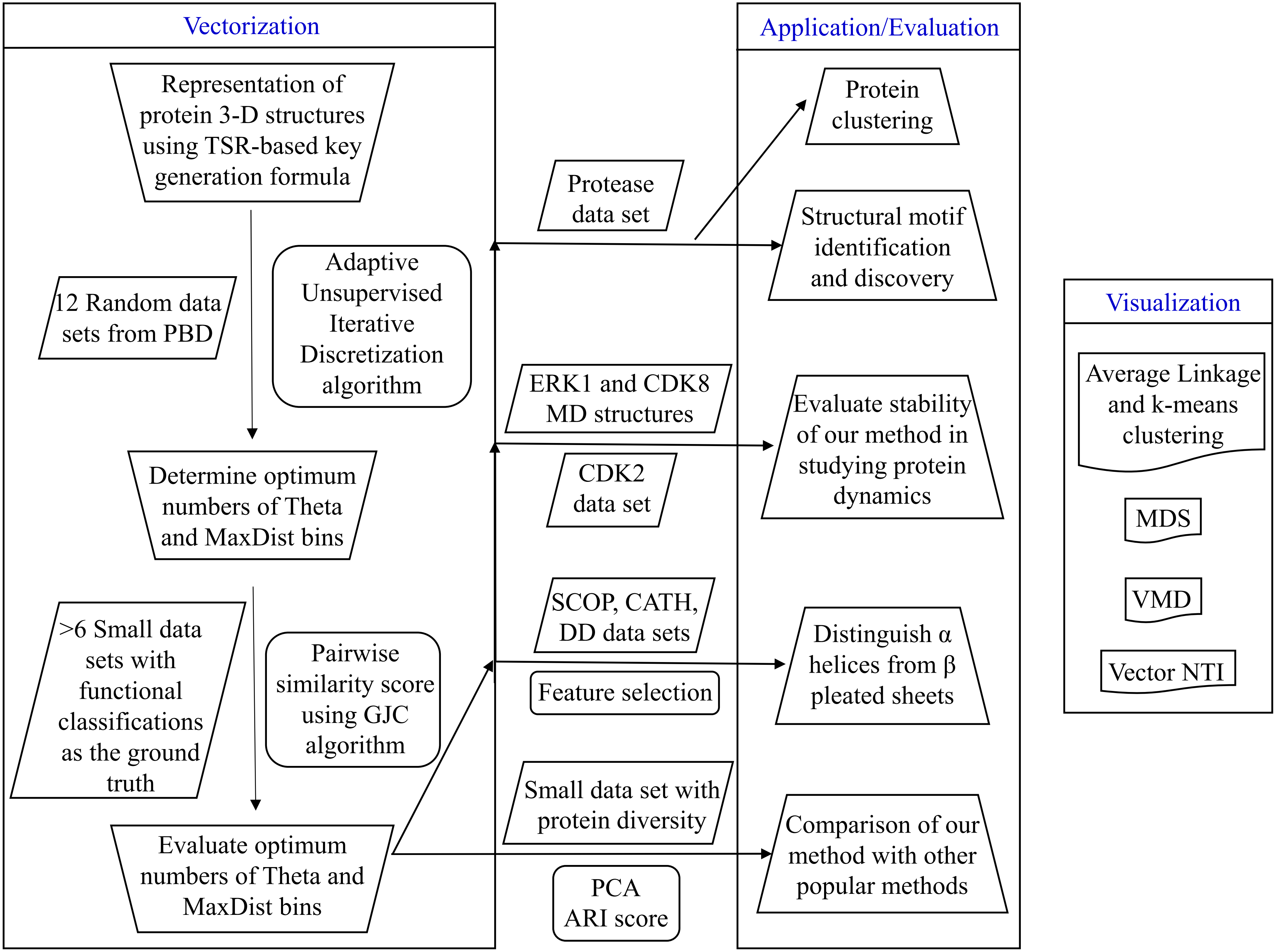
Vectorization
Representation of protein 3-D structures using TSR-based key generation formula
12 Random data sets from PBD
Adaptive Unsupervised Iterative Discretization algorithm
Determine optimum numbers of Theta and MaxDist bins
>6 Small data sets with functional classifications as the ground truth
Pairwise similarity score using GJC algorithm
Evaluate optimum numbers of Theta and MaxDist bins
Protease data set
ERK1 and CDK8 MD structures
CDK2 data set
SCOP, CATH, DD data sets
Feature selection
Small data set with protein diversity
PCA ARI score
Application/Evaluation
Protein clustering
Structural motif identification and discovery
Evaluate stability of our method in studying protein dynamics
Distinguish α helices from β pleated sheets
Comparison of our method with other popular methods
Visualization
Average Linkage and k-means clustering
MDS
VMD
Vector NTI

Figure S2

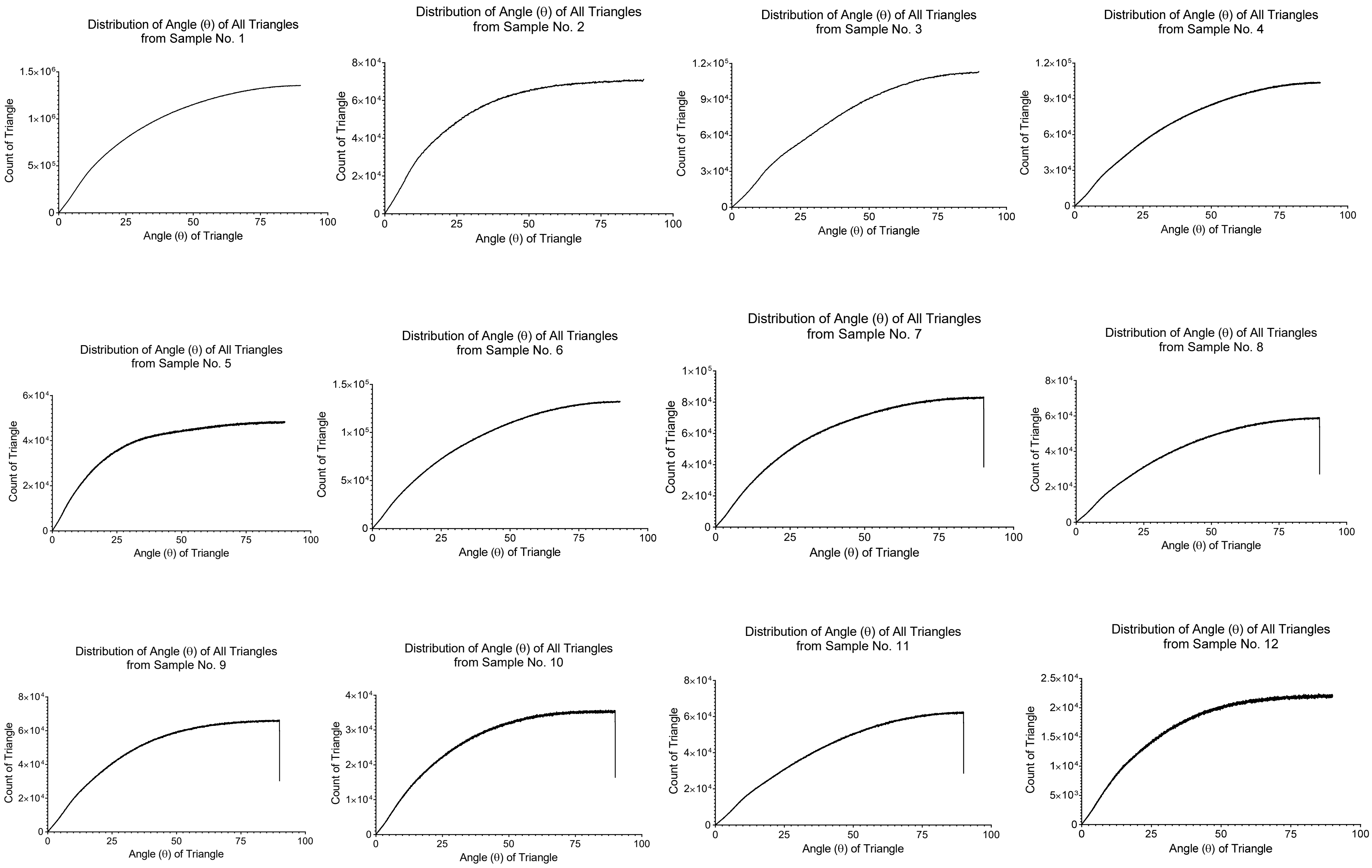

Distribution of Angle (θ) of All Triangles from Sample No. 1
Count of Triangle
Angle (θ) of Triangle
Distribution of Angle (θ) of All Triangles from Sample No. 2
Count of Triangle
Angle (θ) of Triangle
Distribution of Angle (θ) of All Triangles from Sample No. 3
Count of Triangle
Angle (θ) of Triangle
Distribution of Angle (θ) of All Triangles from Sample No. 4
Count of Triangle
Angle (θ) of Triangle
Distribution of Angle (θ) of All Triangles from Sample No. 5
Count of Triangle
Angle (θ) of Triangle
Distribution of Angle (θ) of All Triangles from Sample No. 6
Count of Triangle
Angle (θ) of Triangle
Distribution of Angle (θ) of All Triangles from Sample No. 7
Count of Triangle
Angle (θ) of Triangle
Distribution of Angle (θ) of All Triangles from Sample No. 8
Count of Triangle
Angle (θ) of Triangle
Distribution of Angle (θ) of All Triangles from Sample No. 9
Count of Triangle
Angle (θ) of Triangle
Distribution of Angle (θ) of All Triangles from Sample No. 10
Count of Triangle
Angle (θ) of Triangle
Distribution of Angle (θ) of All Triangles from Sample No. 11
Count of Triangle
Angle (θ) of Triangle
Distribution of Angle (θ) of All Triangles from Sample No. 12
Count of Triangle
Angle (θ) of Triangle

Figure S3

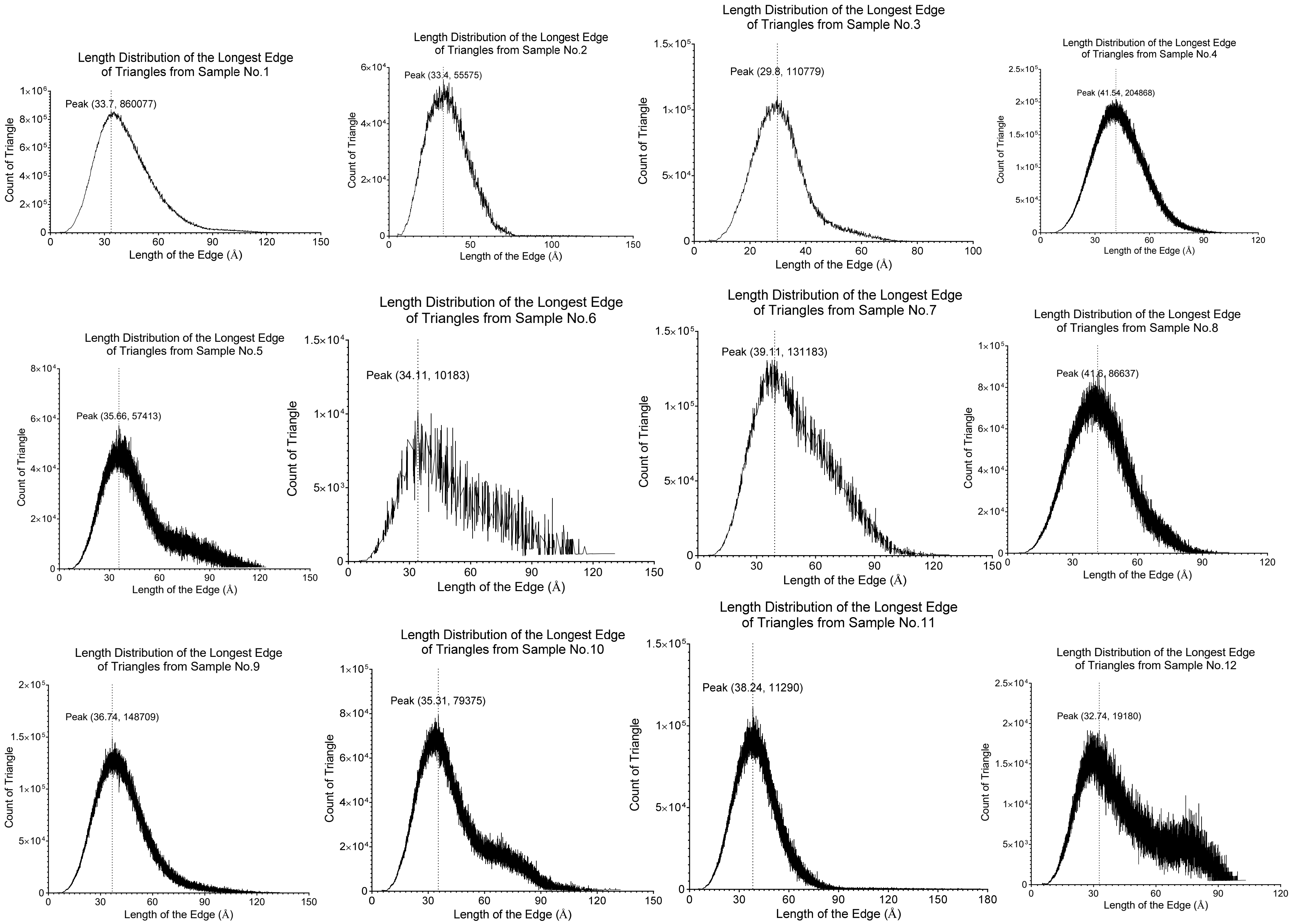

Length Distribution of the Longest Edge of Triangles from Sample No.1
Peak (33.7, 860077)
Count of Triangle
Length of the Edge (Å)
Length Distribution of the Longest Edge of Triangles from Sample No.2
Peak (33.4, 55575)
Count of Triangle
Length of the Edge (Å)
Length Distribution of the Longest Edge of Triangles from Sample No.3
Peak (29.8, 110779)
Count of Triangle
Length of the Edge (Å)
Length Distribution of the Longest Edge of Triangles from Sample No.4
Peak (41.54, 204868)
Count of Triangle
Length of the Edge (Å)
Length Distribution of the Longest Edge of Triangles from Sample No.5
Peak (35.66, 57413)
Count of Triangle
Length of the Edge (Å)
Length Distribution of the Longest Edge of Triangles from Sample No.6
Peak (34.11, 10183)
Count of Triangle
Length of the Edge (Å)
Length Distribution of the Longest Edge of Triangles from Sample No.7
Peak (39.11, 131183)
Count of Triangle
Length of the Edge (Å)
Length Distribution of the Longest Edge of Triangles from Sample No.8
Peak (41.6, 86637)
Count of Triangle
Length of the Edge (Å)
Length Distribution of the Longest Edge of Triangles from Sample No.9
Peak (36.74, 148709)
Count of Triangle
Length of the Edge (Å)
Length Distribution of the Longest Edge of Triangles from Sample No.10
Peak (35.31, 79375)
Count of Triangle
Length of the Edge (Å)
Length Distribution of the Longest Edge of Triangles from Sample No.11
Peak (38.24, 11290)
Count of Triangle
Length of the Edge (Å)
Length Distribution of the Longest Edge of Triangles from Sample No.12
Peak (32.74, 19180)
Count of Triangle
Length of the Edge (Å)

Figure S4

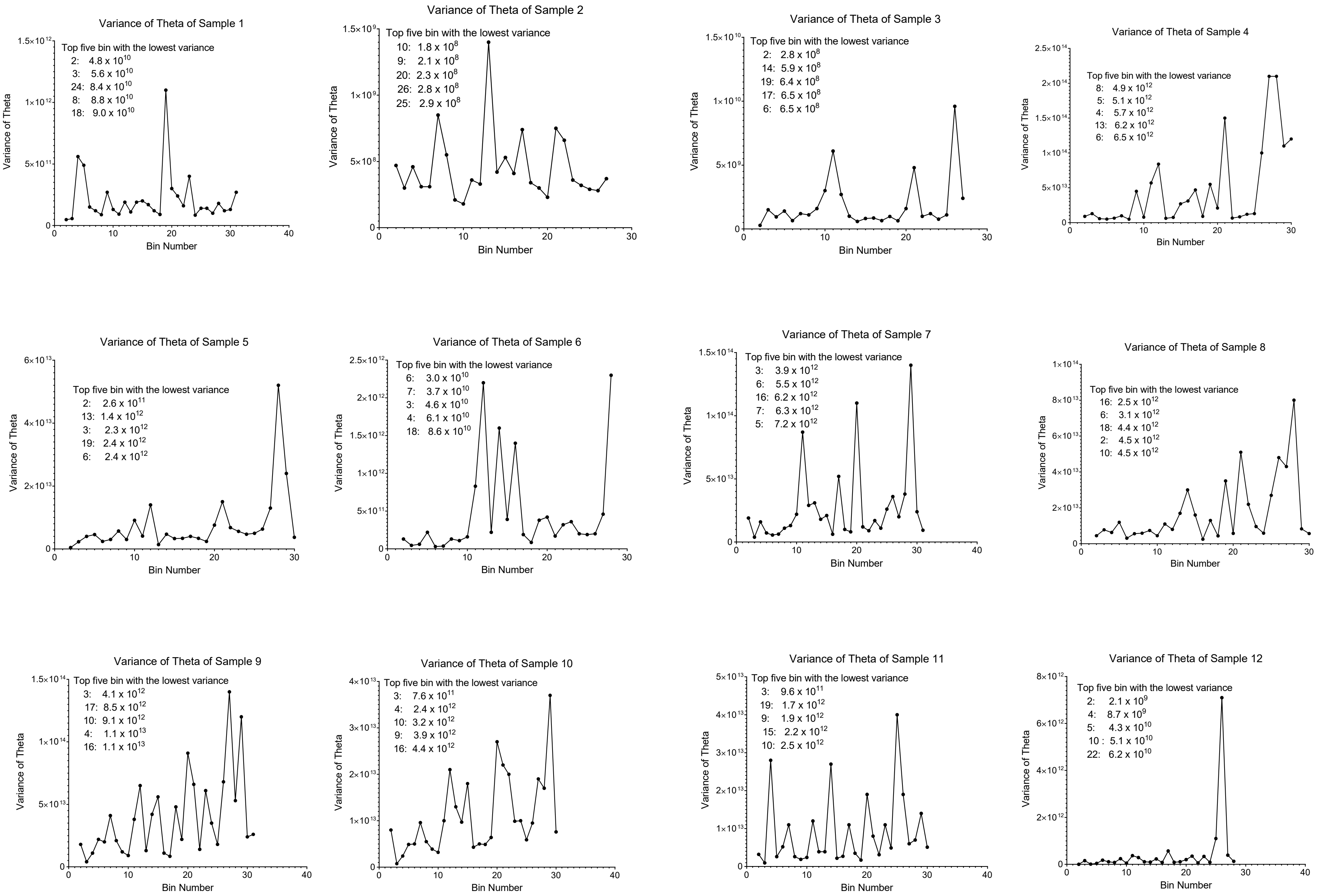
Variance of Theta of Sample 1
Top five bin with the lowest variance
2: 4.8 x 10^{10}
3: 5.6 x 10^{10}
24: 8.4 x 10^{10}
8: 8.8 x 10^{10}
18: 9.0 x 10^{10}
Variance of Theta
Bin Number
Variance of Theta of Sample 2
Top five bin with the lowest variance
10: 1.8 x 10^{8}
9: 2.1 x 10^{8}
20: 2.3 x 10^{8}
26: 2.8 x 10^{8}
25: 2.9 x 10^{8}
Variance of Theta
Bin Number
Variance of Theta of Sample 3
Top five bin with the lowest variance
2: 2.8 x 10^{8}
14: 5.9 x 10^{8}
19: 6.4 x 10^{8}
17: 6.5 x 10^{8}
6: 6.5 x 10^{8}
Variance of Theta
Bin Number
Variance of Theta of Sample 4
Top five bin with the lowest variance
8: 4.9 x 10^{12}
5: 5.1 x 10^{12}
4: 5.7 x 10^{12}
13: 6.2 x 10^{12}
6: 6.5 x 10^{12}
Variance of Theta
Bin Number
Variance of Theta of Sample 5
Top five bin with the lowest variance
2: 2.6 x 10^{11}
13: 1.4 x 10^{12}
3: 2.3 x 10^{12}
19: 2.4 x 10^{12}
6: 2.4 x 10^{12}
Variance of Theta
Bin Number
Variance of Theta of Sample 6
Top five bin with the lowest variance
6: 3.0 x 10^{10}
7: 3.7 x 10^{10}
3: 4.6 x 10^{10}
4: 6.1 x 10^{10}
18: 8.6 x 10^{10}
Variance of Theta
Bin Number
Variance of Theta of Sample 7
Top five bin with the lowest variance
3: 3.9 x 10^{12}
6: 5.5 x 10^{12}
16: 6.2 x 10^{12}
7: 6.3 x 10^{12}
5: 7.2 x 10^{12}
Variance of Theta
Bin Number
Variance of Theta of Sample 8
Top five bin with the lowest variance
16: 2.5 x 10^{12}
6: 3.1 x 10^{12}
18: 4.4 x 10^{12}
2: 4.5 x 10^{12}
10: 4.5 x 10^{12}
Variance of Theta
Bin Number
Variance of Theta of Sample 9
Top five bin with the lowest variance
3: 4.1 x 10^{12}
17: 8.5 x 10^{12}
10: 9.1 x 10^{12}
4: 1.1 x 10^{13}
16: 1.1 x 10^{13}
Variance of Theta
Bin Number
Variance of Theta of Sample 10
Top five bin with the lowest variance
3: 7.6 x 10^{11}
4: 2.4 x 10^{12}
10: 3.2 x 10^{12}
9: 3.9 x 10^{12}
16: 4.4 x 10^{12}
Variance of Theta
Bin Number
Variance of Theta of Sample 11
Top five bin with the lowest variance
3: 9.6 x 10^{11}
19: 1.7 x 10^{12}
9: 1.9 x 10^{12}
15: 2.2 x 10^{12}
10: 2.5 x 10^{12}
Variance of Theta
Bin Number
Variance of Theta of Sample 12
Top five bin with the lowest variance
2: 2.1 x 10^{9}
4: 8.7 x 10^{9}
5: 4.3 x 10^{10}
10 : 5.1 x 10^{10}
22: 6.2 x 10^{10}
Variance of Theta
Bin Number

Figure S5

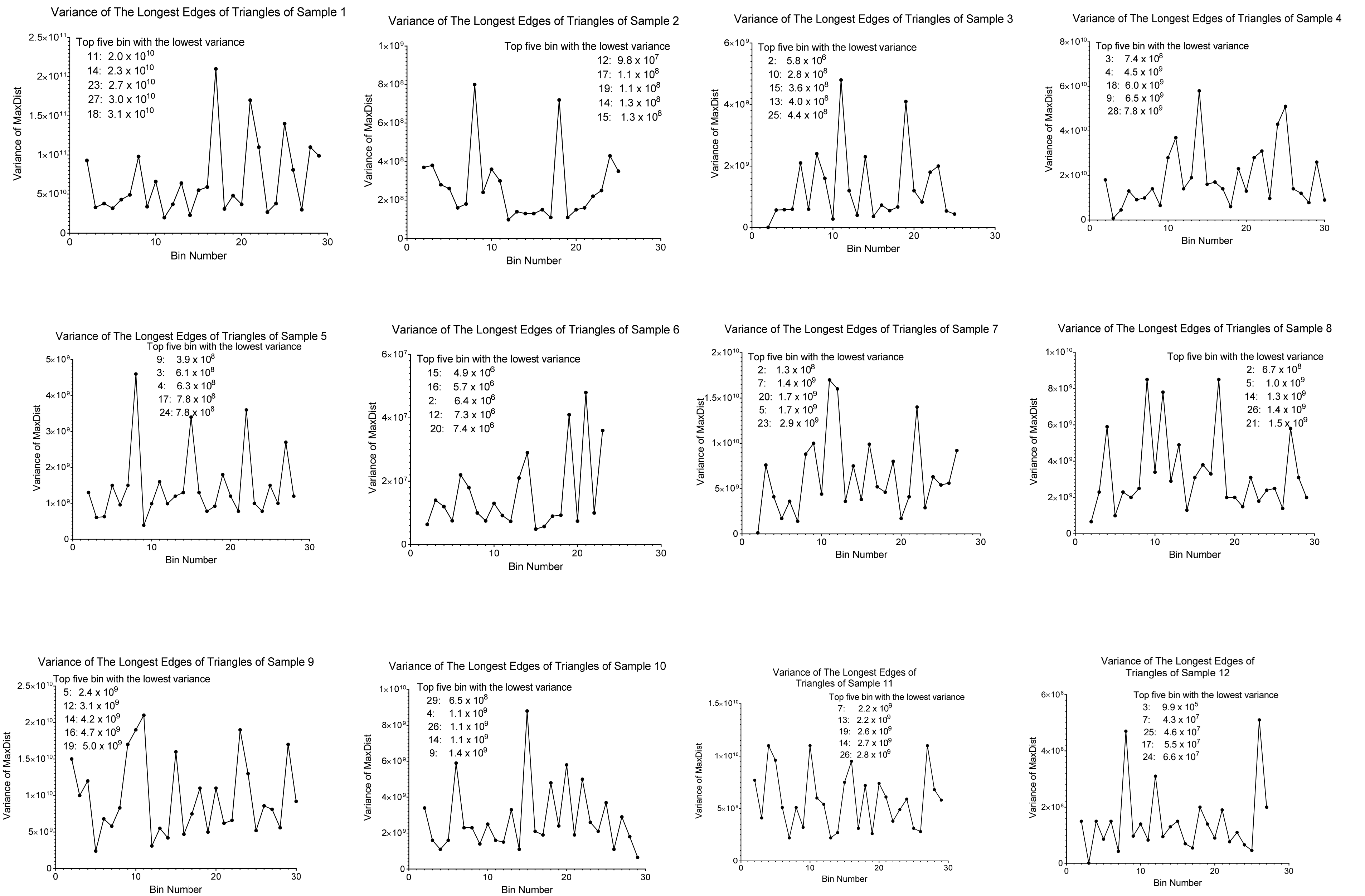
Variance of The Longest Edges of Triangles of Sample 1
Top five bin with the lowest variance
11: 2.0 x 10^10
14: 2.3 x 10^10
23: 2.7 x 10^10
27: 3.0 x 10^10
18: 3.1 x 10^10
Variance of MaxDist
Bin Number
Variance of The Longest Edges of Triangles of Sample 2
Top five bin with the lowest variance
12: 9.8 x 10^7
17: 1.1 x 10^8
19: 1.1 x 10^8
14: 1.3 x 10^8
15: 1.3 x 10^8
Variance of The Longest Edges of Triangles of Sample 3
Top five bin with the lowest variance
2: 5.8 x 10^6
10: 2.8 x 10^8
15: 3.6 x 10^8
13: 4.0 x 10^8
25: 4.4 x 10^8
Variance of The Longest Edges of Triangles of Sample 4
Top five bin with the lowest variance
3: 7.4 x 10^8
4: 4.5 x 10^9
18: 6.0 x 10^9
9: 6.5 x 10^9
28: 7.8 x 10^9
Variance of The Longest Edges of Triangles of Sample 5
Top five bin with the lowest variance
9: 3.9 x 10^8
3: 6.1 x 10^8
4: 6.3 x 10^8
17: 7.8 x 10^8
24: 7.8 x 10^8
Variance of The Longest Edges of Triangles of Sample 6
Top five bin with the lowest variance
15: 4.9 x 10^6
16: 5.7 x 10^6
2: 6.4 x 10^6
12: 7.3 x 10^6
20: 7.4 x 10^6
Variance of The Longest Edges of Triangles of Sample 7
Top five bin with the lowest variance
2: 1.3 x 10^8
7: 1.4 x 10^9
20: 1.7 x 10^9
5: 1.7 x 10^9
23: 2.9 x 10^9
Variance of The Longest Edges of Triangles of Sample 8
Top five bin with the lowest variance
2: 6.7 x 10^8
5: 1.0 x 10^9
14: 1.3 x 10^9
26: 1.4 x 10^9
21: 1.5 x 10^9
Variance of The Longest Edges of Triangles of Sample 9
Top five bin with the lowest variance
5: 2.4 x 10^9
12: 3.1 x 10^9
14: 4.2 x 10^9
16: 4.7 x 10^9
19: 5.0 x 10^9
Variance of The Longest Edges of Triangles of Sample 10
Top five bin with the lowest variance
29: 6.5 x 10^8
4: 1.1 x 10^9
26: 1.1 x 10^9
14: 1.1 x 10^9
9: 1.4 x 10^9
Variance of The Longest Edges of Triangles of Sample 11
Top five bin with the lowest variance
7: 2.2 x 10^9
13: 2.2 x 10^9
19: 2.6 x 10^9
14: 2.7 x 10^9
26: 2.8 x 10^9
Variance of The Longest Edges of Triangles of Sample 12
Top five bin with the lowest variance
3: 9.9 x 10^5
7: 4.3 x 10^7
25: 4.6 x 10^7
17: 5.5 x 10^7
24: 6.6 x 10^7

Figure S6

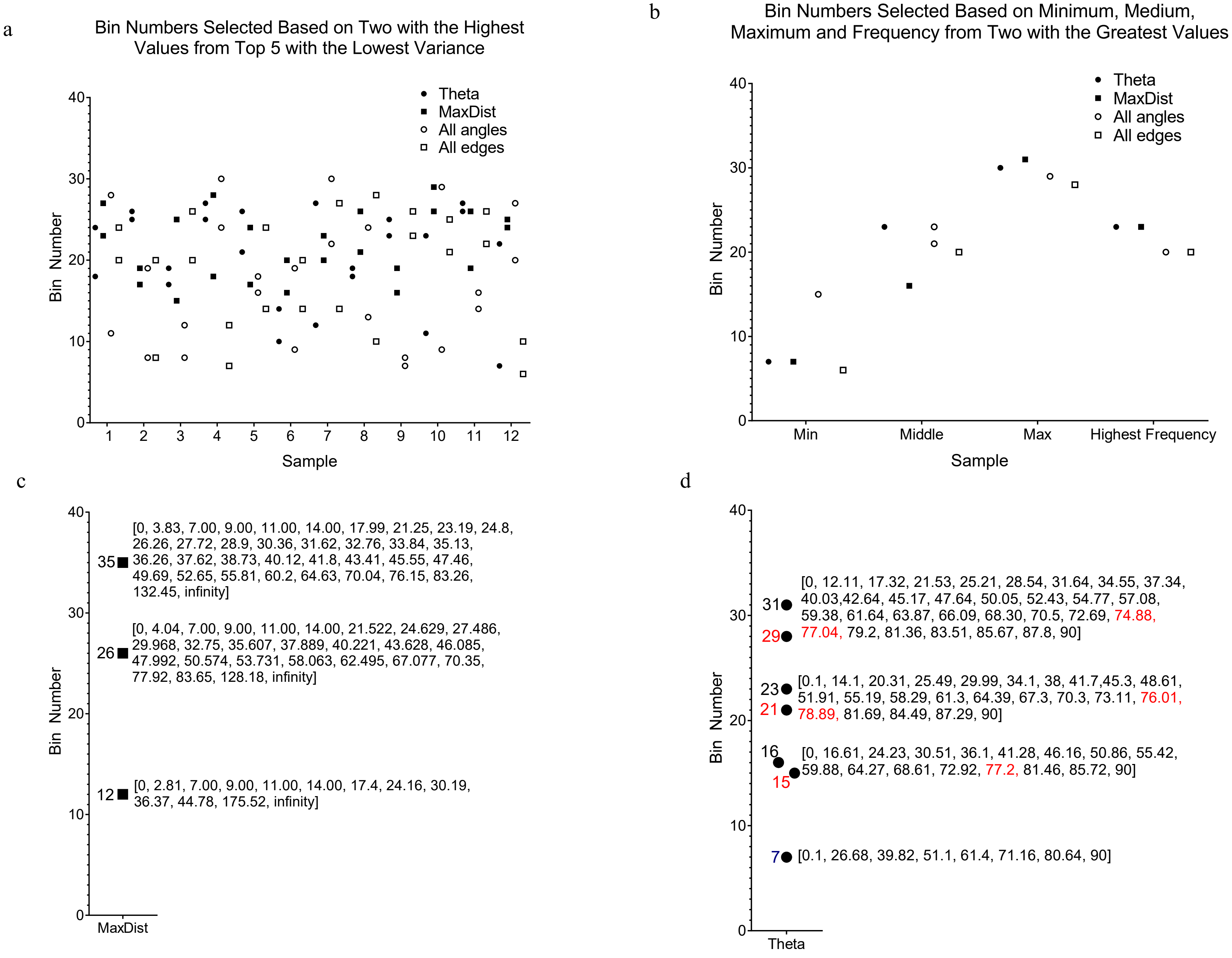
a
Bin Numbers Selected Based on Two with the Highest Values from Top 5 with the Lowest Variance
Theta
MaxDist
All angles
All edges
Bin Number
Sample
b
Bin Numbers Selected Based on Minimum, Medium, Maximum and Frequency from Two with the Greatest Values
Theta
MaxDist
All angles
All edges
Bin Number
Min
Middle
Max
Highest Frequency
Sample
c
35
[0, 3.83, 7.00, 9.00, 11.00, 14.00, 17.99, 21.25, 23.19, 24.8, 26.26, 27.72, 28.9, 30.36, 31.62, 32.76, 33.84, 35.13, 36.26, 37.62, 38.73, 40.12, 41.8, 43.41, 45.55, 47.46, 49.69, 52.65, 55.81, 60.2, 64.63, 70.04, 76.15, 83.26, 132.45, infinity]
26
[0, 4.04, 7.00, 9.00, 11.00, 14.00, 21.522, 24.629, 27.486, 29.968, 32.75, 35.607, 37.889, 40.221, 43.628, 46.085, 47.992, 50.574, 53.731, 58.063, 62.495, 67.077, 70.35, 77.92, 83.65, 128.18, infinity]
12
[0, 2.81, 7.00, 9.00, 11.00, 14.00, 17.4, 24.16, 30.19, 36.37, 44.78, 175.52, infinity]
Bin Number
MaxDist
d
31
29
[0, 12.11, 17.32, 21.53, 25.21, 28.54, 31.64, 34.55, 37.34, 40.03,42.64, 45.17, 47.64, 50.05, 52.43, 54.77, 57.08, 59.38, 61.64, 63.87, 66.09, 68.30, 70.5, 72.69, 74.88, 77.04, 79.2, 81.36, 83.51, 85.67, 87.8, 90]
23
21
[0.1, 14.1, 20.31, 25.49, 29.99, 34.1, 38, 41.7,45.3, 48.61, 51.91, 55.19, 58.29, 61.3, 64.39, 67.3, 70.3, 73.11, 76.01, 78.89, 81.69, 84.49, 87.29, 90]
16
15
[0, 16.61, 24.23, 30.51, 36.1, 41.28, 46.16, 50.86, 55.42, 59.88, 64.27, 68.61, 72.92, 77.2, 81.46, 85.72, 90]
7
[0.1, 26.68, 39.82, 51.1, 61.4, 71.16, 80.64, 90]
Bin Number
Theta

Figure S7

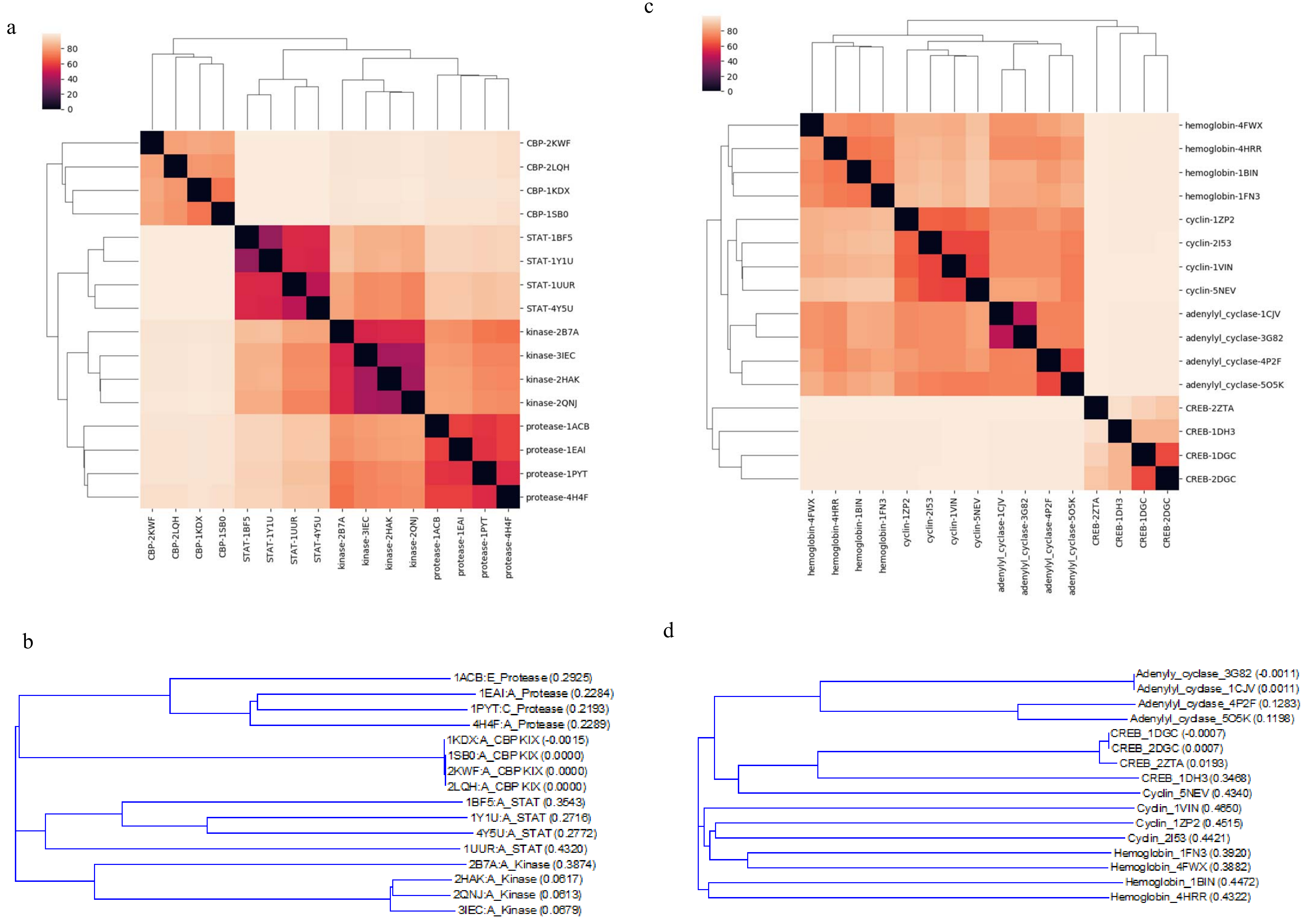
a
80
60
40
20
0
CBP-2KWF
CBP-2LQH
CBP-1KDX
CBP-1SB0
STAT-1BF5
STAT-1Y1U
STAT-1UUR
STAT-4Y5U
kinase-2B7A
kinase-3IEC
kinase-2HAK
kinase-2QNJ
protease-1ACB
protease-1EAI
protease-1PYT
protease-4H4F
b
1ACB:E_Protease (0.2925)
1EAI:A_Protease (0.2284)
1PYT:C_Protease (0.2193)
4H4F:A_Protease (0.2289)
1KDX:A_CBP KIX (-0.0015)
1SB0:A_CBP KIX (0.0000)
2KWF:A_CBP KIX (0.0000)
2LQH:A_CBP KIX (0.0000)
1BF5:A_STAT (0.3543)
1Y1U:A_STAT (0.2716)
4Y5U:A_STAT (0.2772)
1UUR:A_STAT (0.4320)
2B7A:A_Kinase (0.3874)
2HAK:A_Kinase (0.0617)
2QNJ:A_Kinase (0.0613)
3IEC:A_Kinase (0.0679)
c
80
60
40
20
0
hemoglobin-4FWX
hemoglobin-4HRR
hemoglobin-1BIN
hemoglobin-1FN3
cyclin-1ZP2
cyclin-2I53
cyclin-1VIN
cyclin-5NEV
adenylyl_cyclase-1CJV
adenylyl_cyclase-3G82
adenylyl_cyclase-4P2F
adenylyl_cyclase-5O5K
CREB-2ZTA
CREB-1DH3
CREB-1DGC
CREB-2DGC
d
Adenyly_cyclase_3G82 (-0.0011)
Adenylyl_cyclase_1CJV (0.0011)
Adenylyl_cyclase_4P2F (0.1283)
Adenylyl_cyclase_5O5K (0.1198)
CREB_1DGC (-0.0007)
CREB_2DGC (0.0007)
CREB_2ZTA (0.0193)
CREB_1DH3 (0.3468)
Cyclin_5NEV (0.4340)
Cyclin_1VIN (0.4650)
Cyclin_1ZP2 (0.4515)
Cyclin_2I53 (0.4421)
Hemoglobin_1FN3 (0.3920)
Hemoglobin_4FWX (0.3882)
Hemoglobin_1BIN (0.4472)
Hemoglobin_4HRR (0.4322)

Figure S8

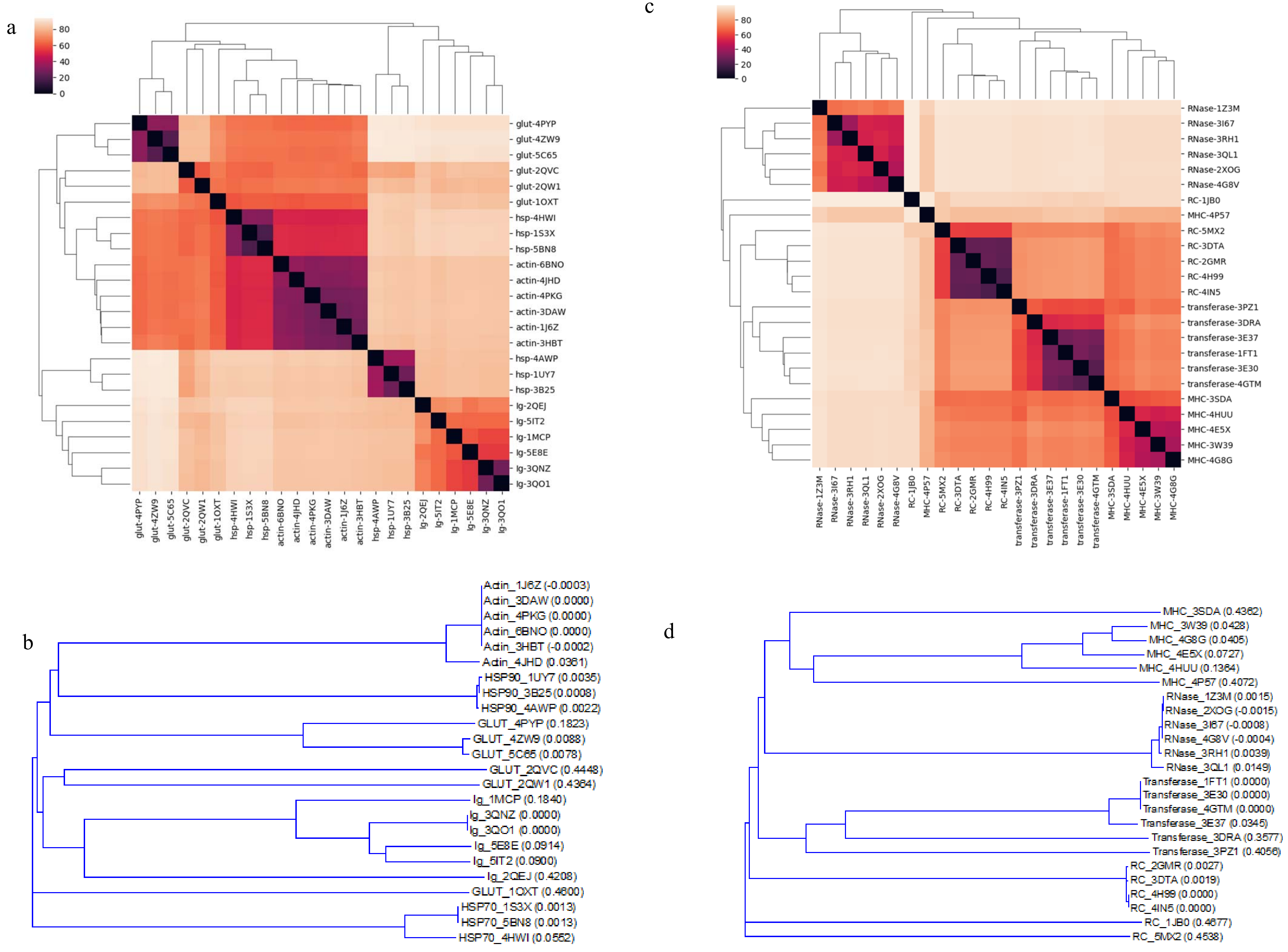
a
80
60
40
20
0
glut-4PYP
glut-4ZW9
glut-5C65
glut-2QVC
glut-2QW1
glut-1OXT
hsp-4HWI
hsp-1S3X
hsp-5BN8
actin-6BNO
actin-4JHD
actin-4PKG
actin-3DAW
actin-1J6Z
actin-3HBT
hsp-4AWP
hsp-1UY7
hsp-3B25
Ig-2QEJ
Ig-5IT2
Ig-1MCP
Ig-5E8E
Ig-3QNZ
Ig-3QO1
b
Actin_1J6Z (-0.0003)
Actin_3DAW (0.0000)
Actin_4PKG (0.0000)
Actin_6BNO (0.0000)
Actin_3HBT (-0.0002)
Actin_4JHD (0.0361)
HSP90_1UY7 (0.0035)
HSP90_3B25 (0.0008)
HSP90_4AWP (0.0022)
GLUT_4PYP (0.1823)
GLUT_4ZW9 (0.0088)
GLUT_5C65 (0.0078)
GLUT_2QVC (0.4448)
GLUT_2QW1 (0.4364)
Ig_1MCP (0.1840)
Ig_3QNZ (0.0000)
Ig_3QO1 (0.0000)
Ig_5E8E (0.0914)
Ig_5IT2 (0.0900)
Ig_2QEJ (0.4208)
GLUT_1OXT (0.4600)
HSP70_1S3X (0.0013)
HSP70_5BN8 (0.0013)
HSP70_4HWI (0.0552)
c
80
60
40
20
0
RNase-1Z3M
RNase-3I67
RNase-3RH1
RNase-3QL1
RNase-2XOG
RNase-4G8V
RC-1JB0
MHC-4P57
RC-5MX2
RC-3DTA
RC-2GMR
RC-4H99
RC-4IN5
transferase-3PZ1
transferase-3DRA
transferase-3E37
transferase-1FT1
transferase-3E30
transferase-4GTM
MHC-3SDA
MHC-4HUU
MHC-4E5X
MHC-3W39
MHC-4G8G
d
MHC_3SDA (0.4362)
MHC_3W39 (0.0428)
MHC_4G8G (0.0405)
MHC_4E5X (0.0727)
MHC_4HUU (0.1364)
MHC_4P57 (0.4072)
RNase_1Z3M (0.0015)
RNase_2XOG (-0.0015)
RNase_3I67 (-0.0008)
RNase_4G8V (-0.0004)
RNase_3RH1 (0.0039)
RNase_3QL1 (0.0149)
Transferase_1FT1 (0.0000)
Transferase_3E30 (0.0000)
Transferase_4GTM (0.0000)
Transferase_3E37 (0.0345)
Transferase_3DRA (0.3577)
Transferase_3PZ1 (0.4056)
RC_2GMR (0.0027)
RC_3DTA (0.0019)
RC_4H99 (0.0000)
RC_4IN5 (0.0000)
RC_1JB0 (0.4677)
RC_5MX2 (0.4538)

Figure S9

a

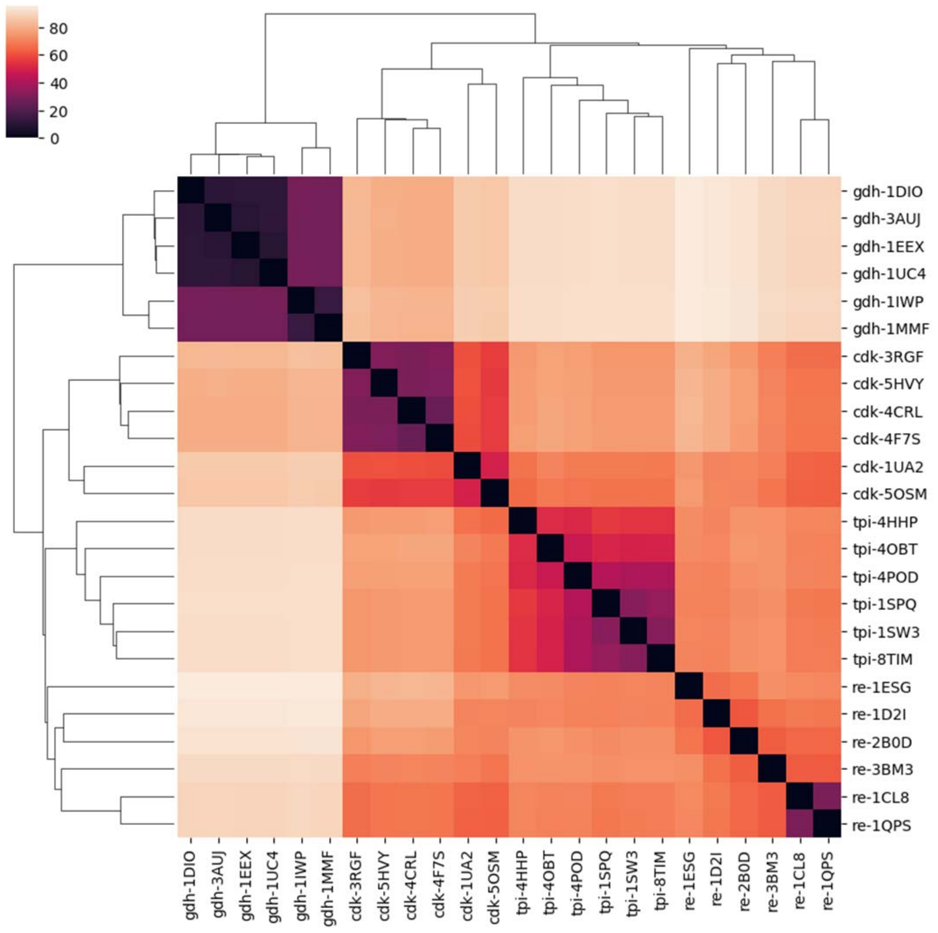
80
60
40
20
0
gdh-1DIO
gdh-3AUJ
gdh-1EEX
gdh-1UC4
gdh-1IWP
gdh-1MMF
cdk-3RGF
cdk-5HVY
cdk-4CRL
cdk-4F7S
cdk-1UA2
cdk-5OSM
tpi-4HHP
tpi-4OBT
tpi-4POD
tpi-1SPQ
tpi-1SW3
tpi-8TIM
re-1ESG
re-1D2I
re-2B0D
re-3BM3
re-1CL8
re-1QPS

b

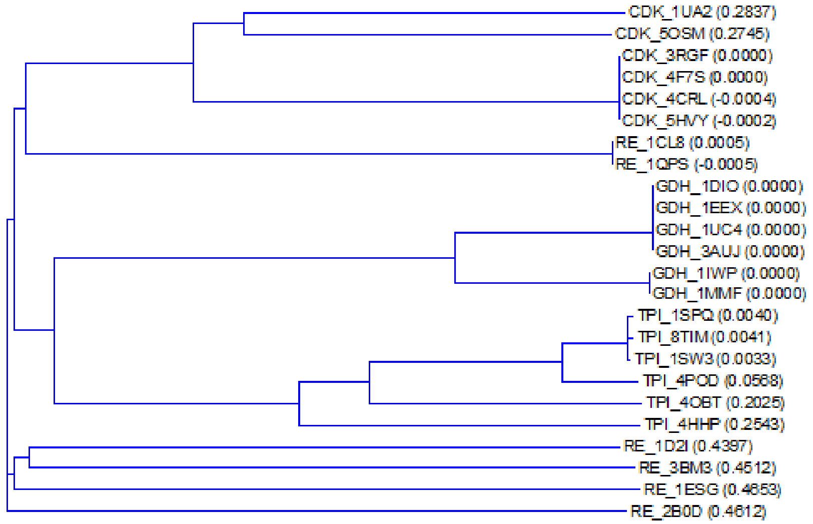
CDK_1UA2 (0.2837)
CDK_5OSM (0.2745)
CDK_3RGF (0.0000)
CDK_4F7S (0.0000)
CDK_4CRL (-0.0004)
CDK_5HVY (-0.0002)
RE_1CL8 (0.0005)
RE_1QPS (-0.0005)
GDH_1DIO (0.0000)
GDH_1EEX (0.0000)
GDH_1UC4 (0.0000)
GDH_3AUJ (0.0000)
GDH_1IWP (0.0000)
GDH_1MMF (0.0000)
TPI_1SPQ (0.0040)
TPI_8TIM (0.0041)
TPI_1SW3 (0.0033)
TPI_4POD (0.0568)
TPI_4OBT (0.2025)
TPI_4HHP (0.2543)
RE_1D2I (0.4397)
RE_3BM3 (0.4512)
RE_1ESG (0.4653)
RE_2B0D (0.4612)

Figure S10

a

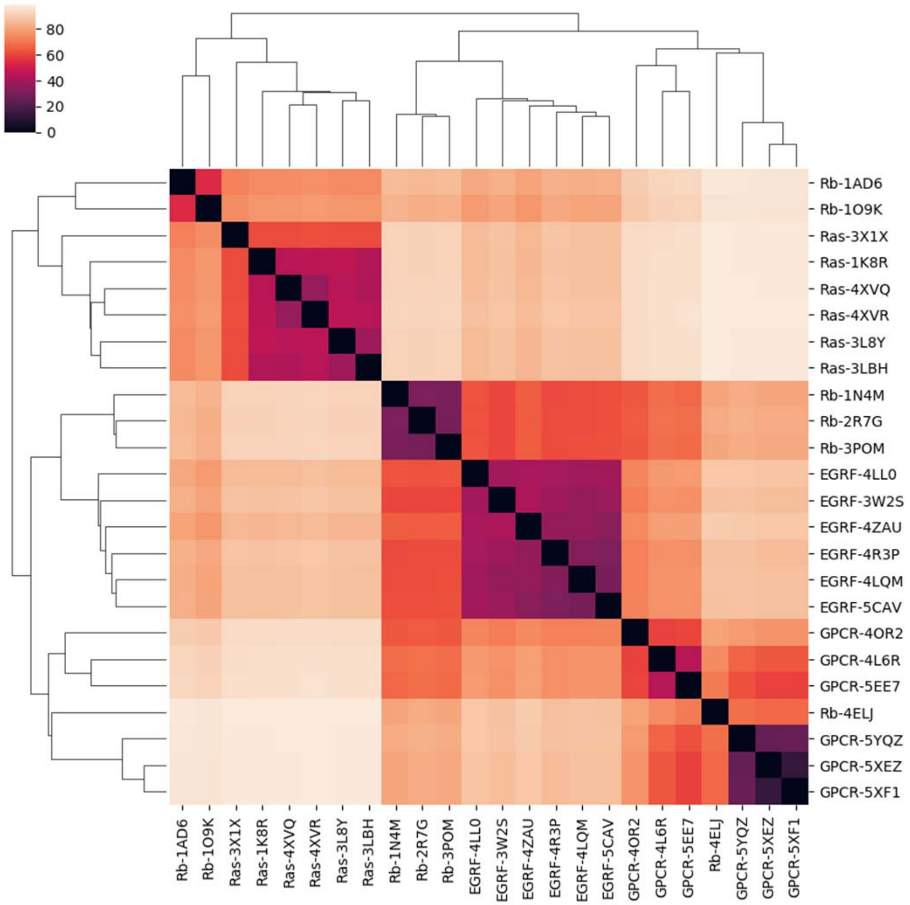

b

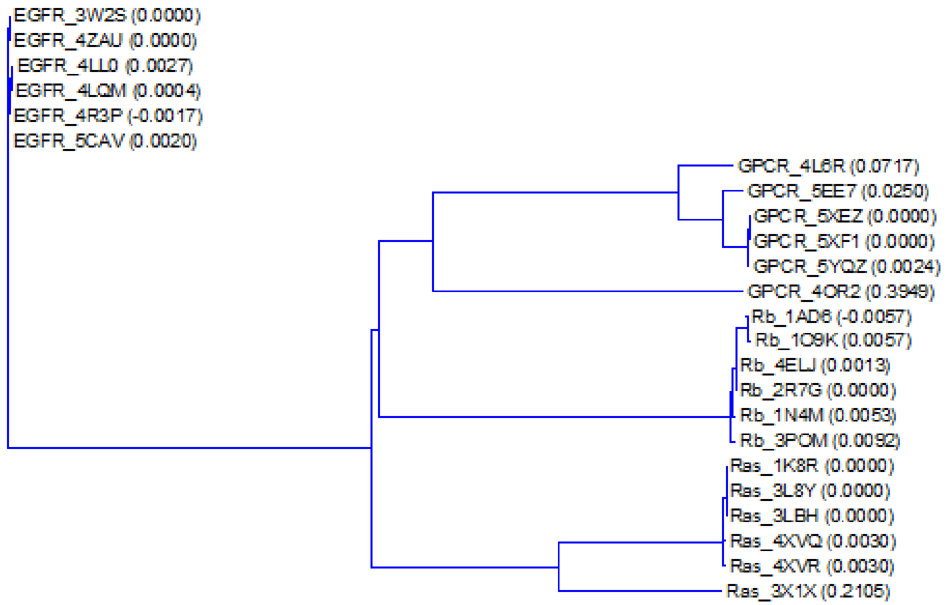

Figure S11

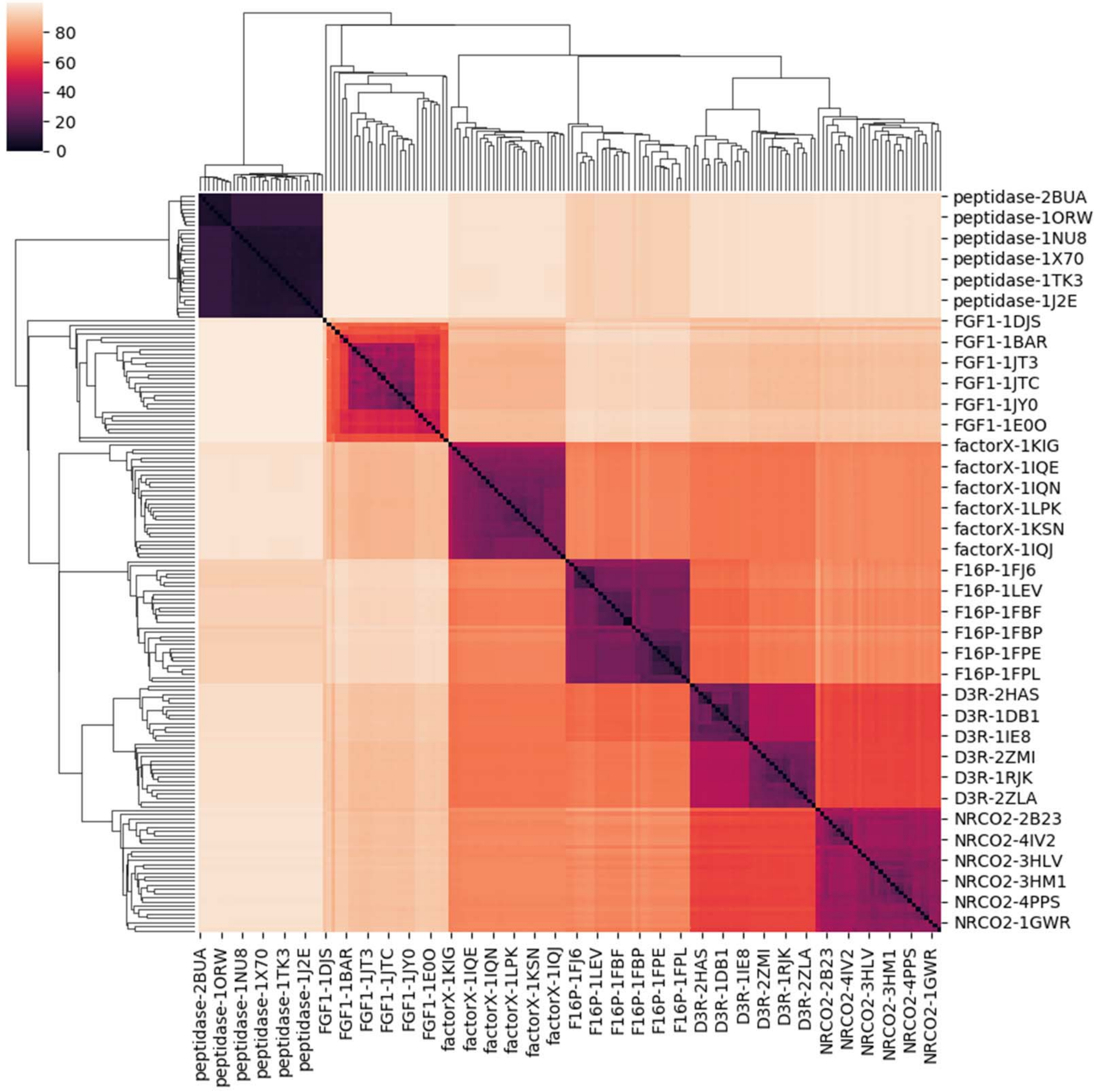
80
60
40
20
0
peptidase-2BUA
peptidase-1ORW
peptidase-1NU8
peptidase-1X70
peptidase-1TK3
peptidase-1J2E
FGF1-1DJS
FGF1-1BAR
FGF1-1JT3
FGF1-1JTC
FGF1-1JY0
FGF1-1E0O
factorX-1KIG
factorX-1IQE
factorX-1IQN
factorX-1LPK
factorX-1KSN
factorX-1IQJ
F16P-1FJ6
F16P-1LEV
F16P-1FBF
F16P-1FBP
F16P-1FPE
F16P-1FPL
D3R-2HAS
D3R-1DB1
D3R-1IE8
D3R-2ZMI
D3R-1RJK
D3R-2ZLA
NRCO2-2B23
NRCO2-4IV2
NRCO2-3HLV
NRCO2-3HM1
NRCO2-4PPS
NRCO2-1GWR

Figure S12

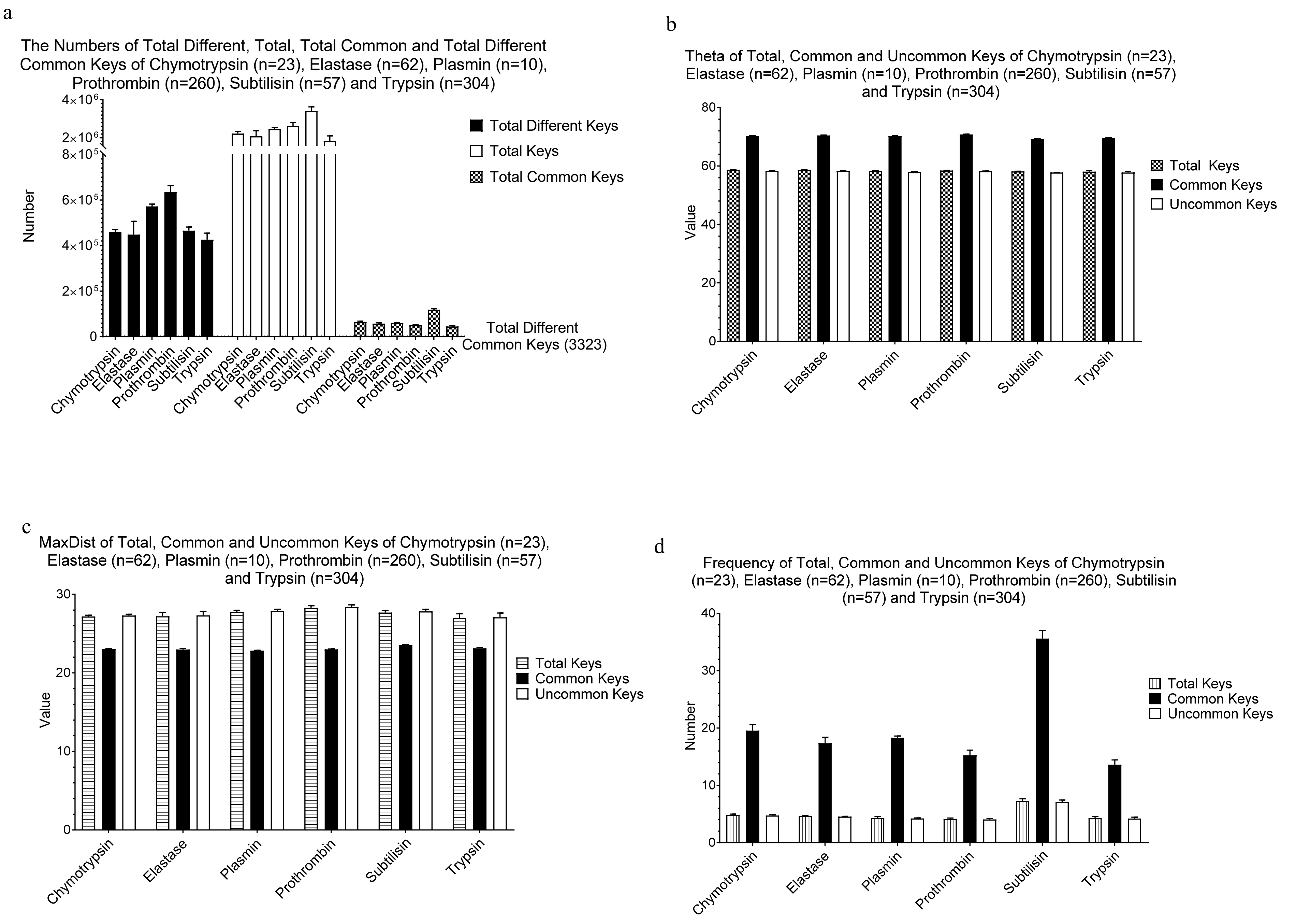
a
The Numbers of Total Different, Total, Total Common and Total Different Common Keys of Chymotrypsin (n=23), Elastase (n=62), Plasmin (n=10), Prothrombin (n=260), Subtilisin (n=57) and Trypsin (n=304)
Number
Total Different Keys
Total Keys
Total Common Keys
Total Different Common Keys (3323)
Chymotrypsin
Elastase
Plasmin
Prothrombin
Subtilisin
Trypsin
b
Theta of Total, Common and Uncommon Keys of Chymotrypsin (n=23), Elastase (n=62), Plasmin (n=10), Prothrombin (n=260), Subtilisin (n=57) and Trypsin (n=304)
Value
Total Keys
Common Keys
Uncommon Keys
c
MaxDist of Total, Common and Uncommon Keys of Chymotrypsin (n=23), Elastase (n=62), Plasmin (n=10), Prothrombin (n=260), Subtilisin (n=57) and Trypsin (n=304)
Value
Total Keys
Common Keys
Uncommon Keys
d
Frequency of Total, Common and Uncommon Keys of Chymotrypsin (n=23), Elastase (n=62), Plasmin (n=10), Prothrombin (n=260), Subtilisin (n=57) and Trypsin (n=304)
Number
Total Keys
Common Keys
Uncommon Keys

Figure S13

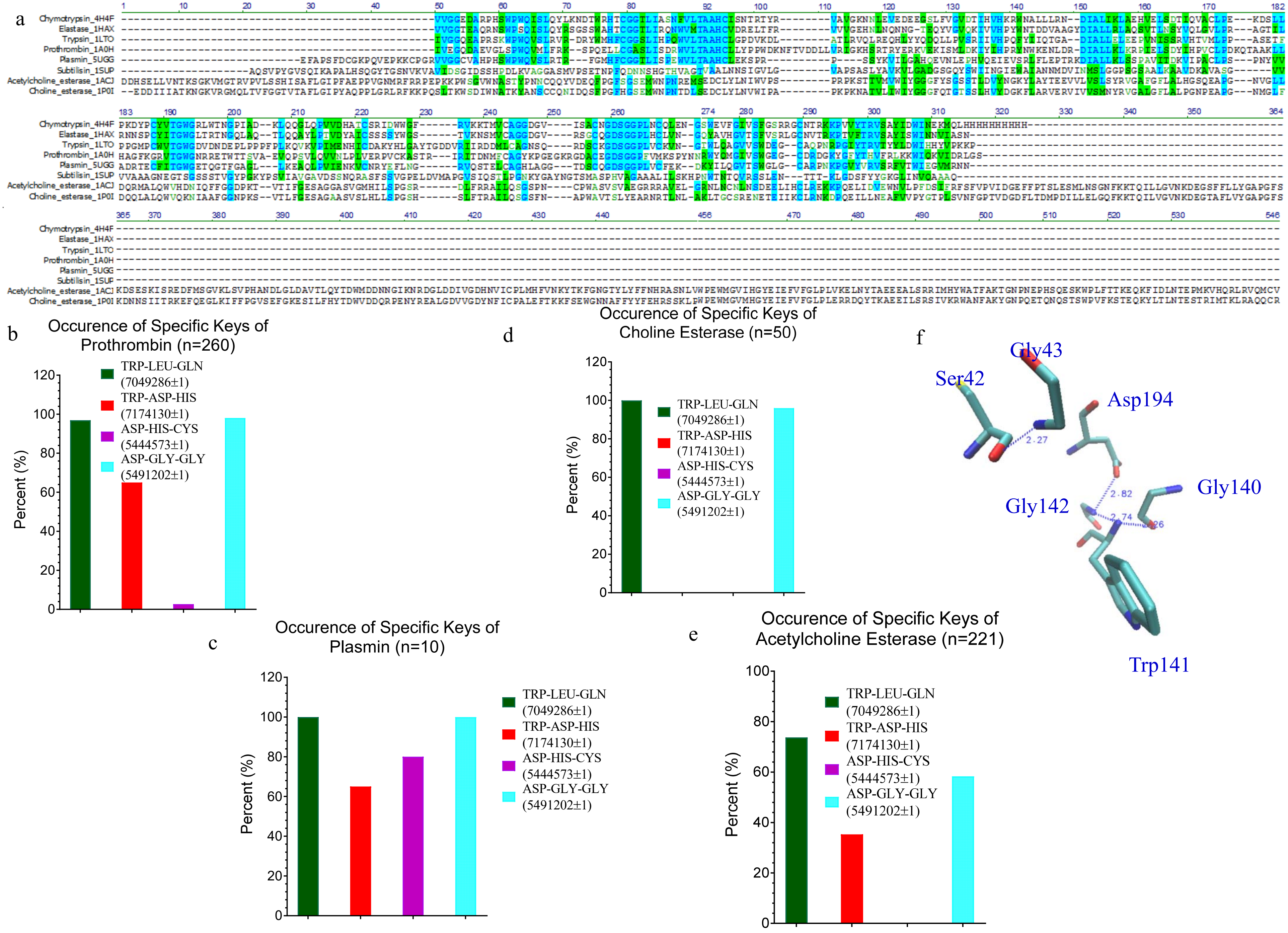
a
Chymotrypsin_4H4F
Elastase_1HAX
Trypsin_1LTO
Prothrombin_1A0H
Plasmin_5UGG
Subtilisin_1SUP
Acetylcholine_esterase_1ACJ
Choline_esterase_1P0I
b
Occurence of Specific Keys of Prothrombin (n=260)
Percent (%)
TRP-LEU-GLN (7049286±1)
TRP-ASP-HIS (7174130±1)
ASP-HIS-CYS (5444573±1)
ASP-GLY-GLY (5491202±1)
c
Occurence of Specific Keys of Plasmin (n=10)
Percent (%)
TRP-LEU-GLN (7049286±1)
TRP-ASP-HIS (7174130±1)
ASP-HIS-CYS (5444573±1)
ASP-GLY-GLY (5491202±1)
d
Occurence of Specific Keys of Choline Esterase (n=50)
Percent (%)
TRP-LEU-GLN (7049286±1)
TRP-ASP-HIS (7174130±1)
ASP-HIS-CYS (5444573±1)
ASP-GLY-GLY (5491202±1)
e
Occurence of Specific Keys of Acetylcholine Esterase (n=221)
Percent (%)
TRP-LEU-GLN (7049286±1)
TRP-ASP-HIS (7174130±1)
ASP-HIS-CYS (5444573±1)
ASP-GLY-GLY (5491202±1)
f
Gly43
Ser42
Asp194
2.27
2.82
2.74
Gly140
Gly142
Trp141

Figure S14

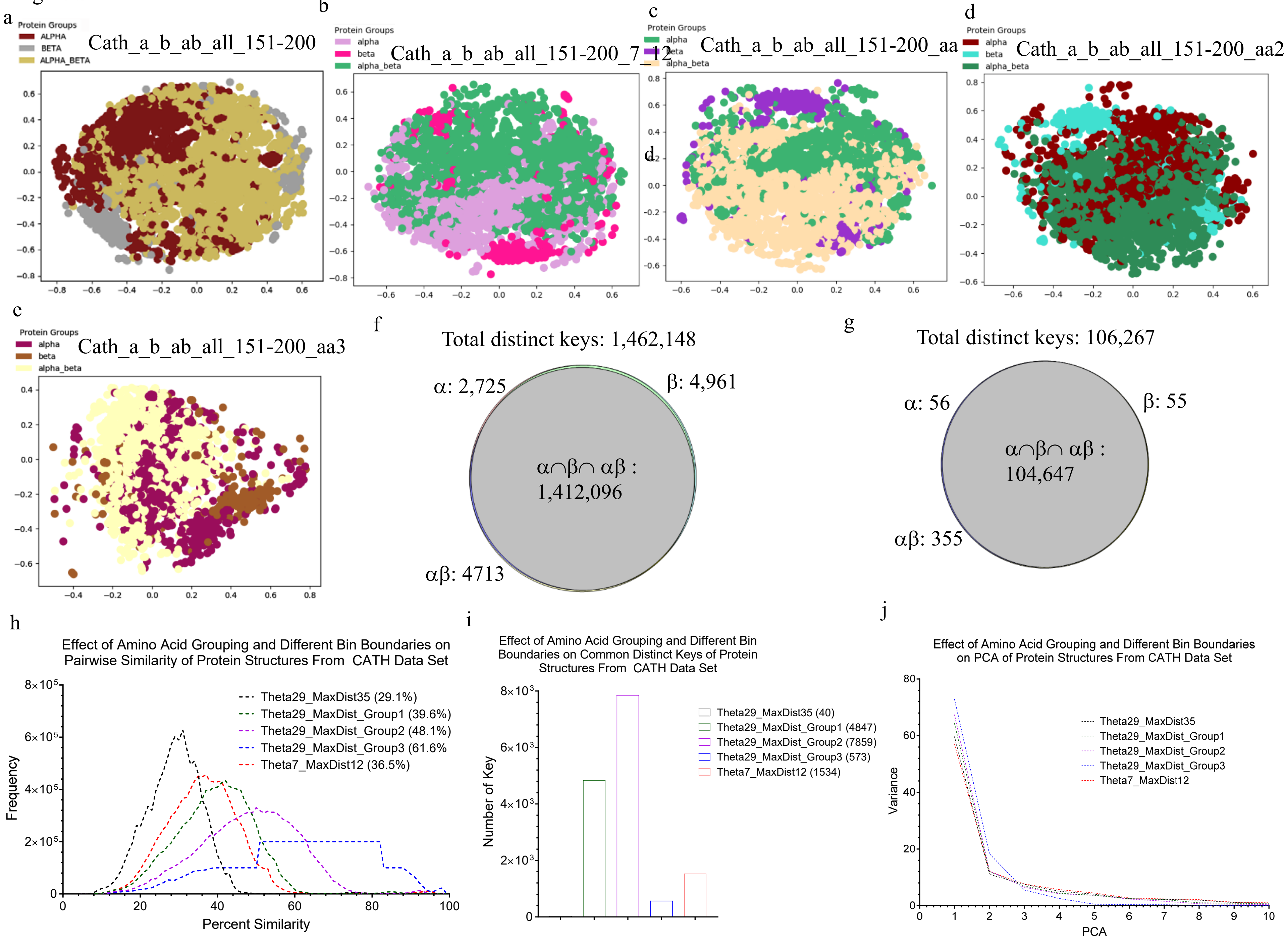
a
Protein Groups
ALPHA
BETA
ALPHA_BETA
Cath_a_b_ab_all_151-200
b
Protein Groups
alpha
beta
alpha_beta
Cath_a_b_ab_all_151-200_7_12
c
Protein Groups
alpha
beta
alpha_beta
Cath_a_b_ab_all_151-200_aa
d
Protein Groups
alpha
beta
alpha_beta
Cath_a_b_ab_all_151-200_aa2
e
Protein Groups
alpha
beta
alpha_beta
Cath_a_b_ab_all_151-200_aa3
f
Total distinct keys: 1,462,148
α: 2,725
β: 4,961
α∩β∩ αβ : 1,412,096
αβ: 4713
g
Total distinct keys: 106,267
α: 56
β: 55
α∩β∩ αβ : 104,647
αβ: 355
h
Effect of Amino Acid Grouping and Different Bin Boundaries on Pairwise Similarity of Protein Structures From CATH Data Set
Theta29_MaxDist35 (29.1%)
Theta29_MaxDist_Group1 (39.6%)
Theta29_MaxDist_Group2 (48.1%)
Theta29_MaxDist_Group3 (61.6%
Theta7_MaxDist12 (36.5%)
Frequency
Percent Similarity
i
Effect of Amino Acid Grouping and Different Bin Boundaries on Common Distinct Keys of Protein Structures From CATH Data Set
Theta29_MaxDist35 (40)
Theta29_MaxDist_Group1 (4847)
Theta29_MaxDist_Group2 (7859)
Theta29_MaxDist_Group3 (573)
Theta7_MaxDist12 (1534)
Number of Key
j
Effect of Amino Acid Grouping and Different Bin Boundaries on PCA of Protein Structures From CATH Data Set
Theta29_MaxDist35
Theta29_MaxDist_Group1
Theta29_MaxDist_Group2
Theta29_MaxDist_Group3
Theta7_MaxDist12
Variance
PCA

Figure S15

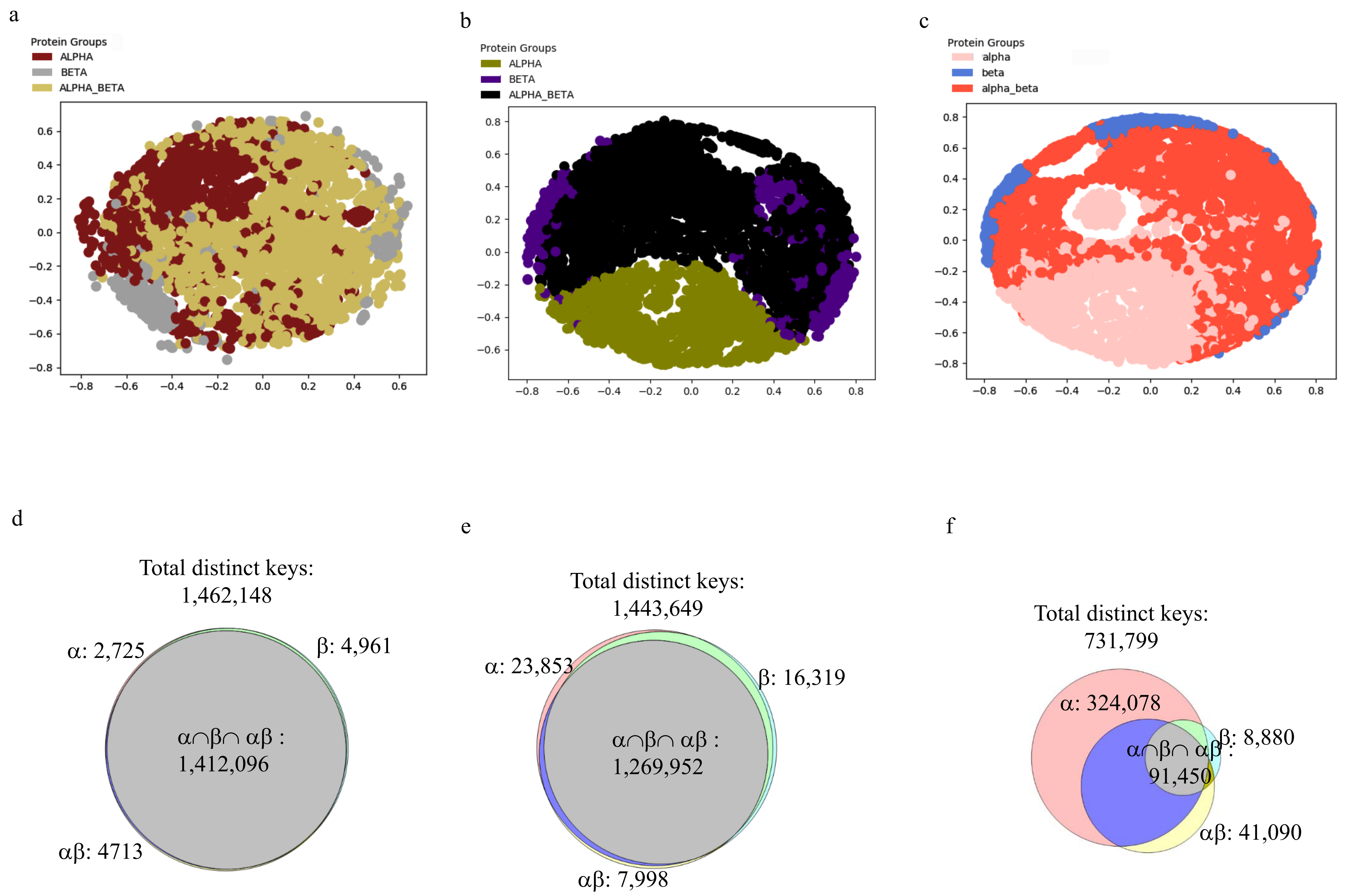

a
Protein Groups
ALPHA
BETA
ALPHA_BETA
b
Protein Groups
ALPHA
BETA
ALPHA_BETA
c
Protein Groups
alpha
beta
alpha_beta
d
Total distinct keys:
1,462,148
α: 2,725
β: 4,961
α∩β∩ αβ :
1,412,096
αβ: 4713
e
Total distinct keys:
1,443,649
α: 23,853
β: 16,319
α∩β∩ αβ :
1,269,952
αβ: 7,998
f
Total distinct keys:
731,799
α: 324,078
β: 8,880
α∩β∩ αβ :
91,450
αβ: 41,090

Figure S16

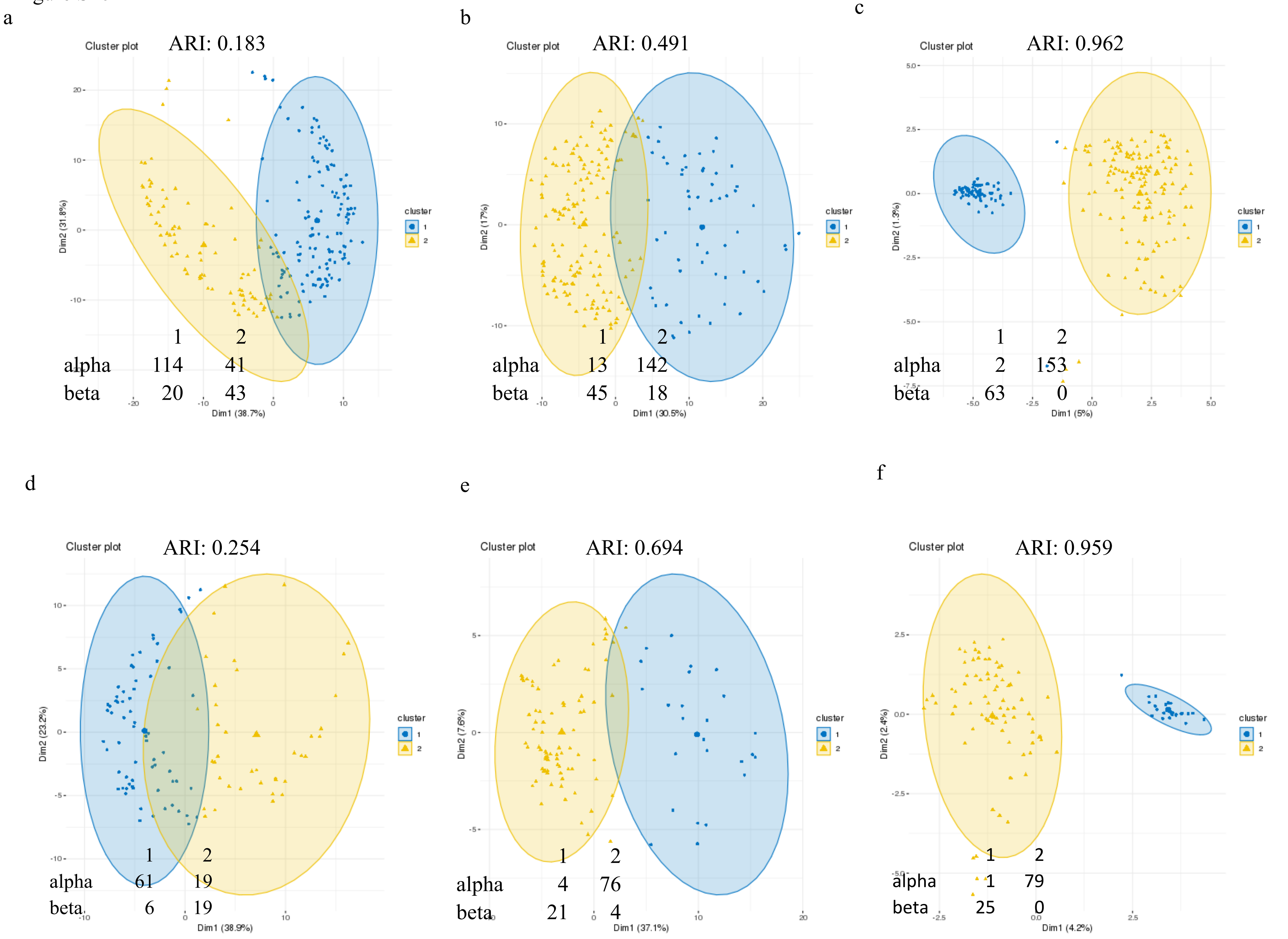

a
Cluster plot
ARI: 0.183
1 2
alpha 114 41
beta 20 43
Dim1 (38.7%)
Dim2 (31.8%)
b
Cluster plot
ARI: 0.491
1 2
alpha 13 142
beta 45 18
Dim1 (30.5%)
Dim2 (17%)
c
Cluster plot
ARI: 0.962
1 2
alpha 2 153
beta 63 0
Dim1 (5%)
Dim2 (1.3%)
d
Cluster plot
ARI: 0.254
1 2
alpha 61 19
beta 6 19
Dim1 (38.9%)
Dim2 (23.2%)
e
Cluster plot
ARI: 0.694
1 2
alpha 4 76
beta 21 4
Dim1 (37.1%)
Dim2 (7.6%)
f
Cluster plot
ARI: 0.959
1 2
alpha 1 79
beta 25 0
Dim1 (4.2%)
Dim2 (2.4%)
cluster
1
2

Figure S17

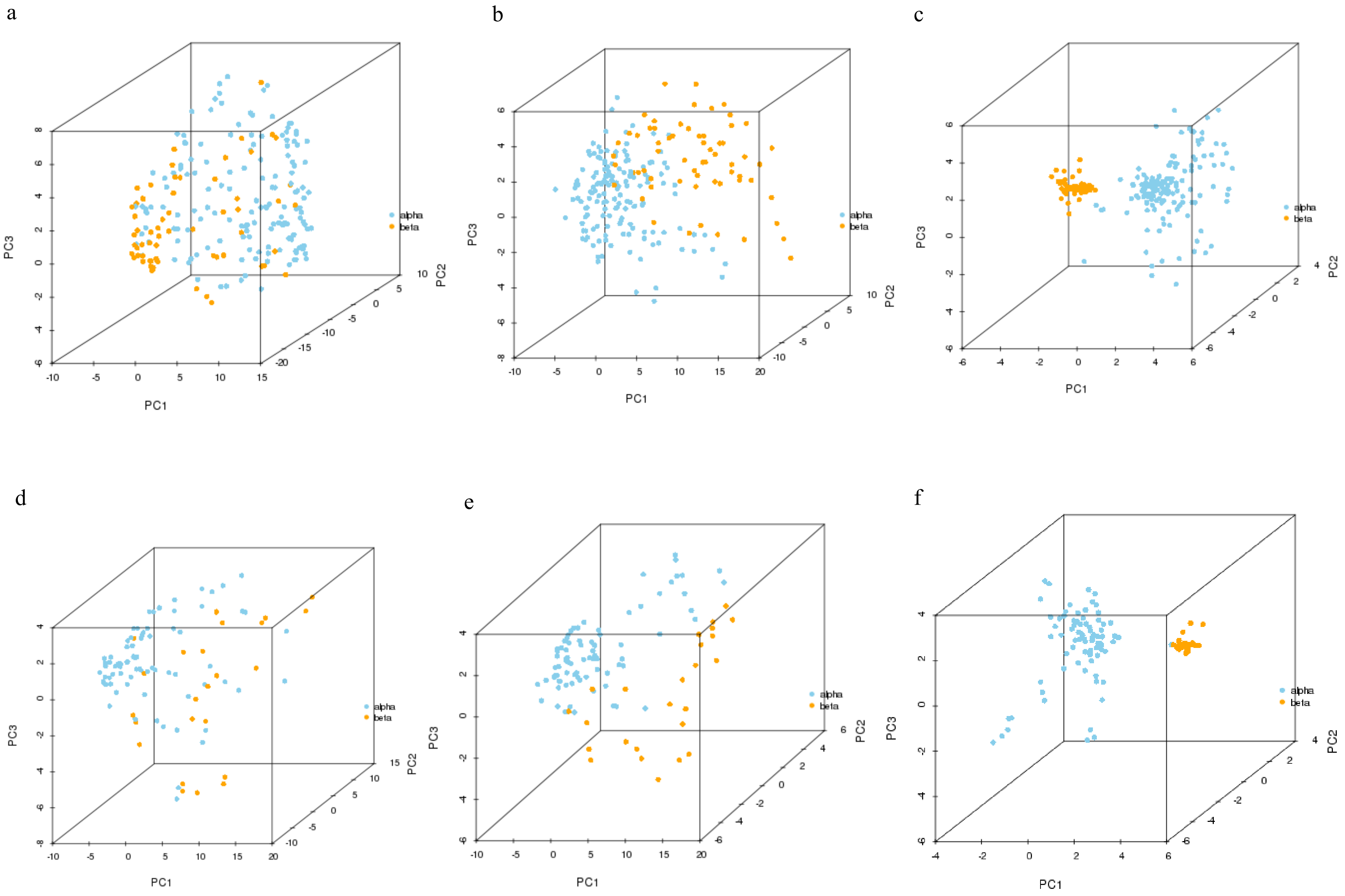
a
PC1
PC2
PC3
alpha
beta
b
PC1
PC2
PC3
alpha
beta
c
PC1
PC2
PC3
alpha
beta
d
PC1
PC2
PC3
alpha
beta
e
PC1
PC2
PC3
alpha
beta
f
PC1
PC2
PC3
alpha
beta

Figure S18

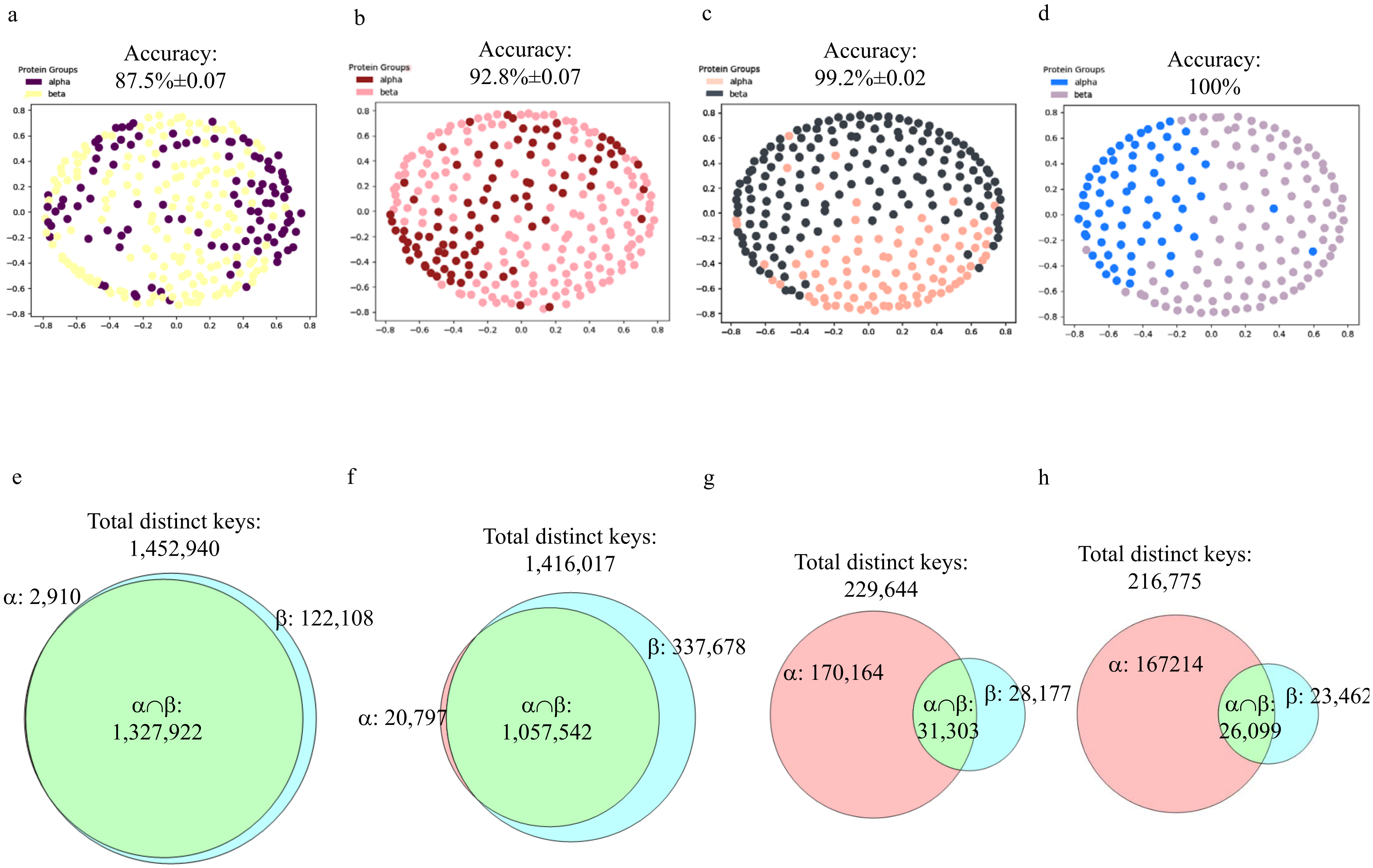

a
Accuracy: 87.5%±0.07
Protein Groups
alpha
beta
b
Accuracy: 92.8%±0.07
Protein Groups
alpha
beta
c
Accuracy: 99.2%±0.02
Protein Groups
alpha
beta
d
Accuracy: 100%
Protein Groups
alpha
beta
e
Total distinct keys: 1,452,940
α: 2,910
β: 122,108
α∩β: 1,327,922
f
Total distinct keys: 1,416,017
β: 337,678
α: 20,797
α∩β: 1,057,542
g
Total distinct keys: 229,644
α: 170,164
α∩β: 31,303
β: 28,177
h
Total distinct keys: 216,775
α: 167214
α∩β: 26,099
β: 23,462

Figure S19

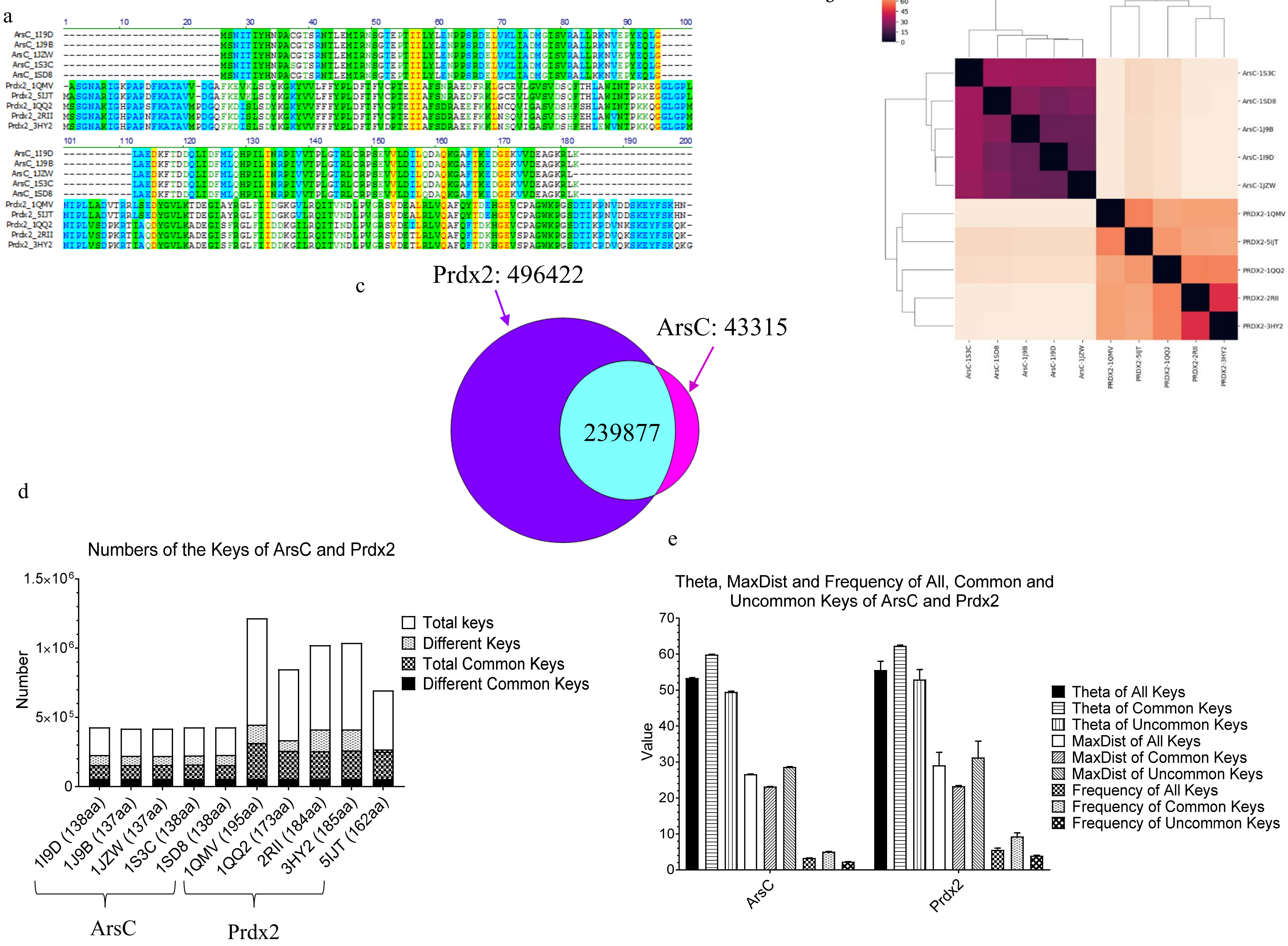

a
ArsC_1I9D
ArsC_1J9B
ArsC_1JZW
ArsC_1S3C
ArsC_1SD8
Prdx2_1QMV
Prdx2_5IJT
Prdx2_1QQ2
Prdx2_2RII
Prdx2_3HY2
b
ArsC-1S3C
ArsC-1SD8
ArsC-1J9B
ArsC-1I9D
ArsC-1JZW
PRDX2-1QMV
PRDX2-5IJT
PRDX2-1QQ2
PRDX2-2RII
PRDX2-3HY2
c
Prdx2: 496422
ArsC: 43315
239877
d
Numbers of the Keys of ArsC and Prdx2
Number
Total keys
Different Keys
Total Common Keys
Different Common Keys
1I9D (138aa)
1J9B (137aa)
1JZW (137aa)
1S3C (138aa)
1SD8 (138aa)
1QMV (195aa)
1QQ2 (173aa)
2RII (184aa)
3HY2 (185aa)
5IJT (162aa)
ArsC
Prdx2
e
Theta, MaxDist and Frequency of All, Common and Uncommon Keys of ArsC and Prdx2
Value
Theta of All Keys
Theta of Common Keys
Theta of Uncommon Keys
MaxDist of All Keys
MaxDist of Common Keys
MaxDist of Uncommon Keys
Frequency of All Keys
Frequency of Common Keys
Frequency of Uncommon Keys
ArsC
Prdx2

Figure S20

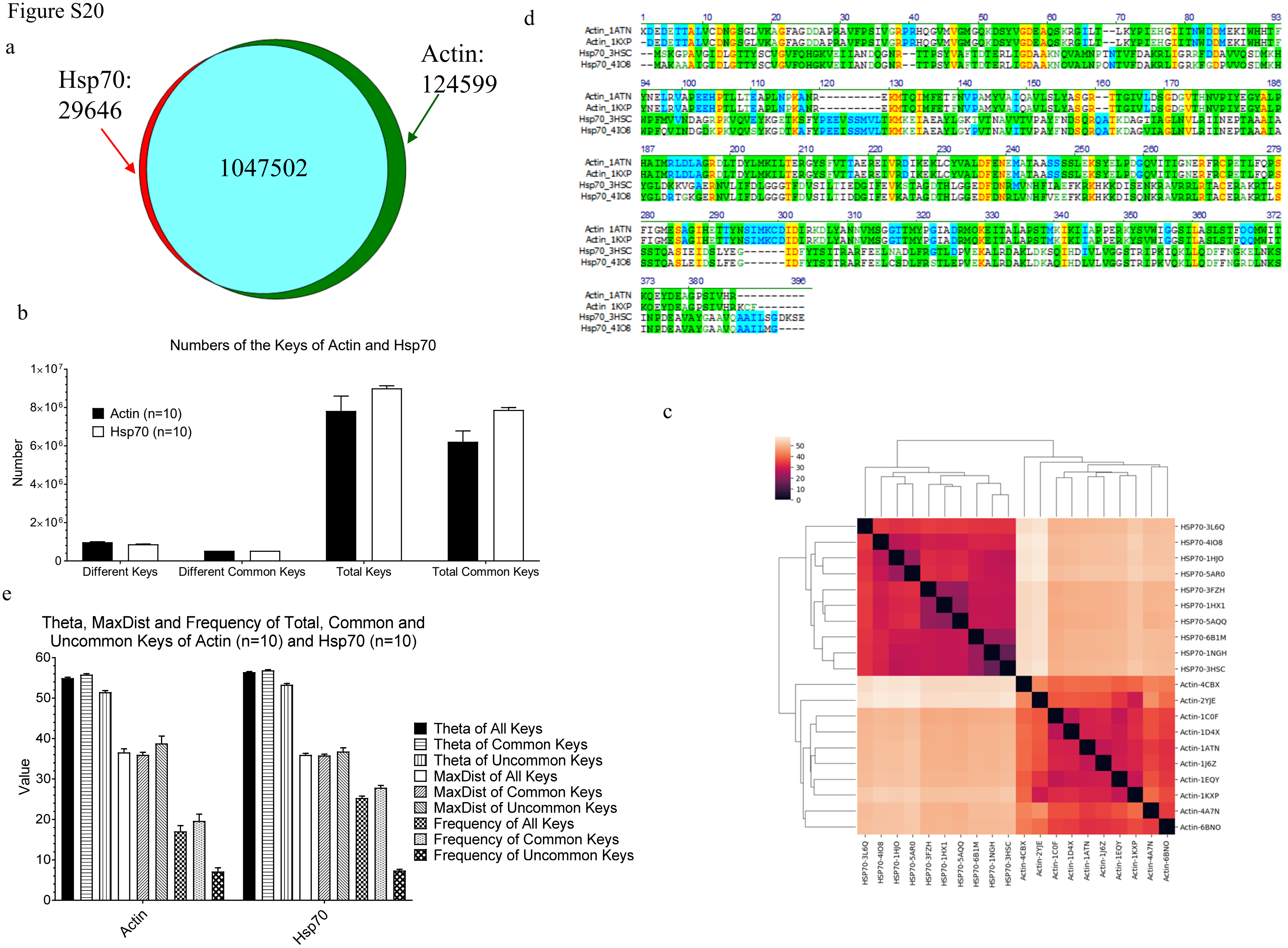
a
Hsp70: 29646
Actin: 124599
1047502
b
Numbers of the Keys of Actin and Hsp70
Actin (n=10)
Hsp70 (n=10)
Number
Different Keys
Different Common Keys
Total Keys
Total Common Keys
c
d
Actin_1ATN
Actin_1KXP
Hsp70_3HSC
Hsp70_4IO8
e
Theta, MaxDist and Frequency of Total, Common and Uncommon Keys of Actin (n=10) and Hsp70 (n=10)
Value
Theta of All Keys
Theta of Common Keys
Theta of Uncommon Keys
MaxDist of All Keys
MaxDist of Common Keys
MaxDist of Uncommon Keys
Frequency of All Keys
Frequency of Common Keys
Frequency of Uncommon Keys
Actin
Hsp70

Figure S21

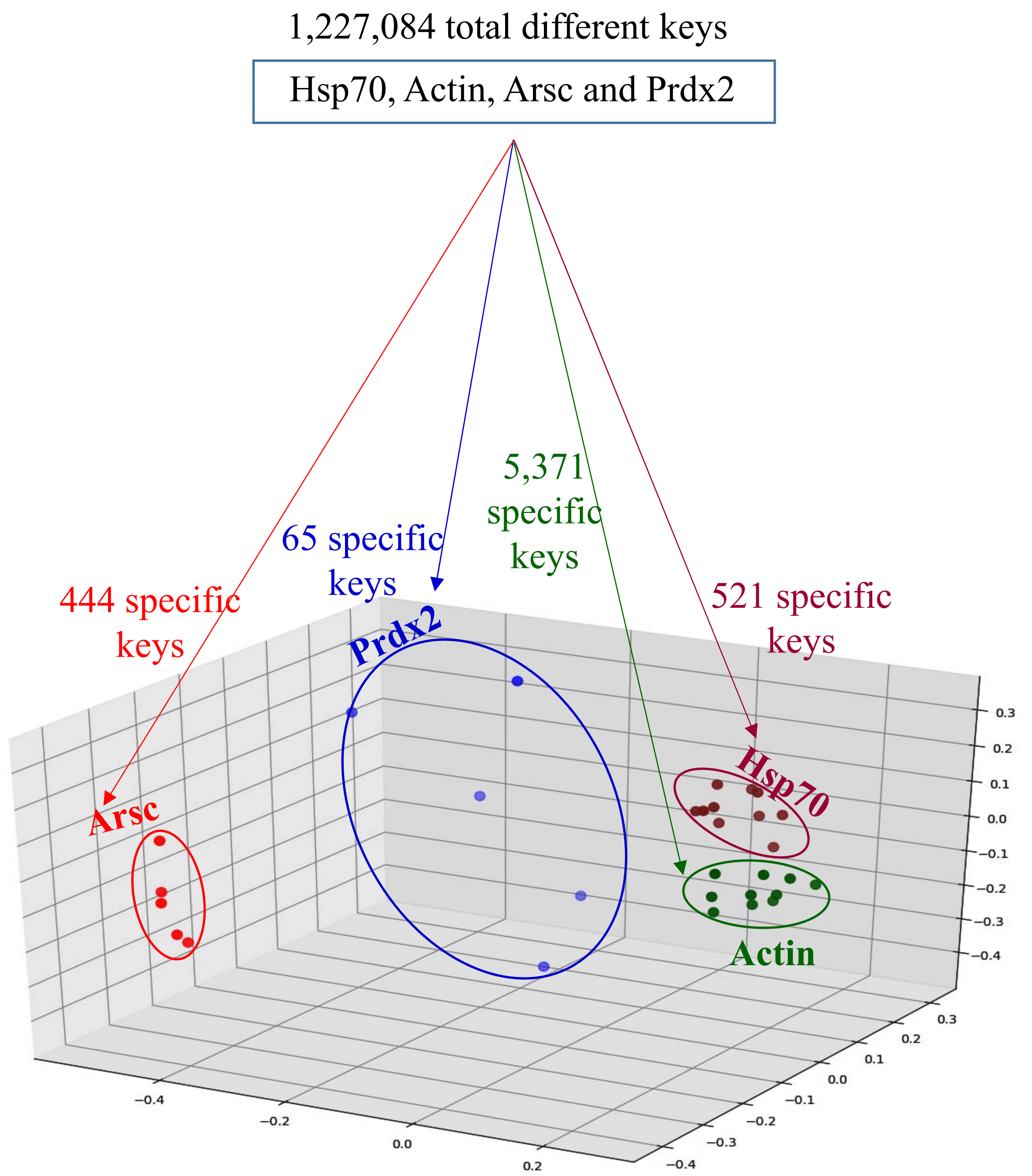
1,227,084 total different keys
Hsp70, Actin, Arsc and Prdx2
444 specific keys
65 specific keys
5,371 specific keys
521 specific keys
Arsc
Prdx2
Hsp70
Actin

Figure S22

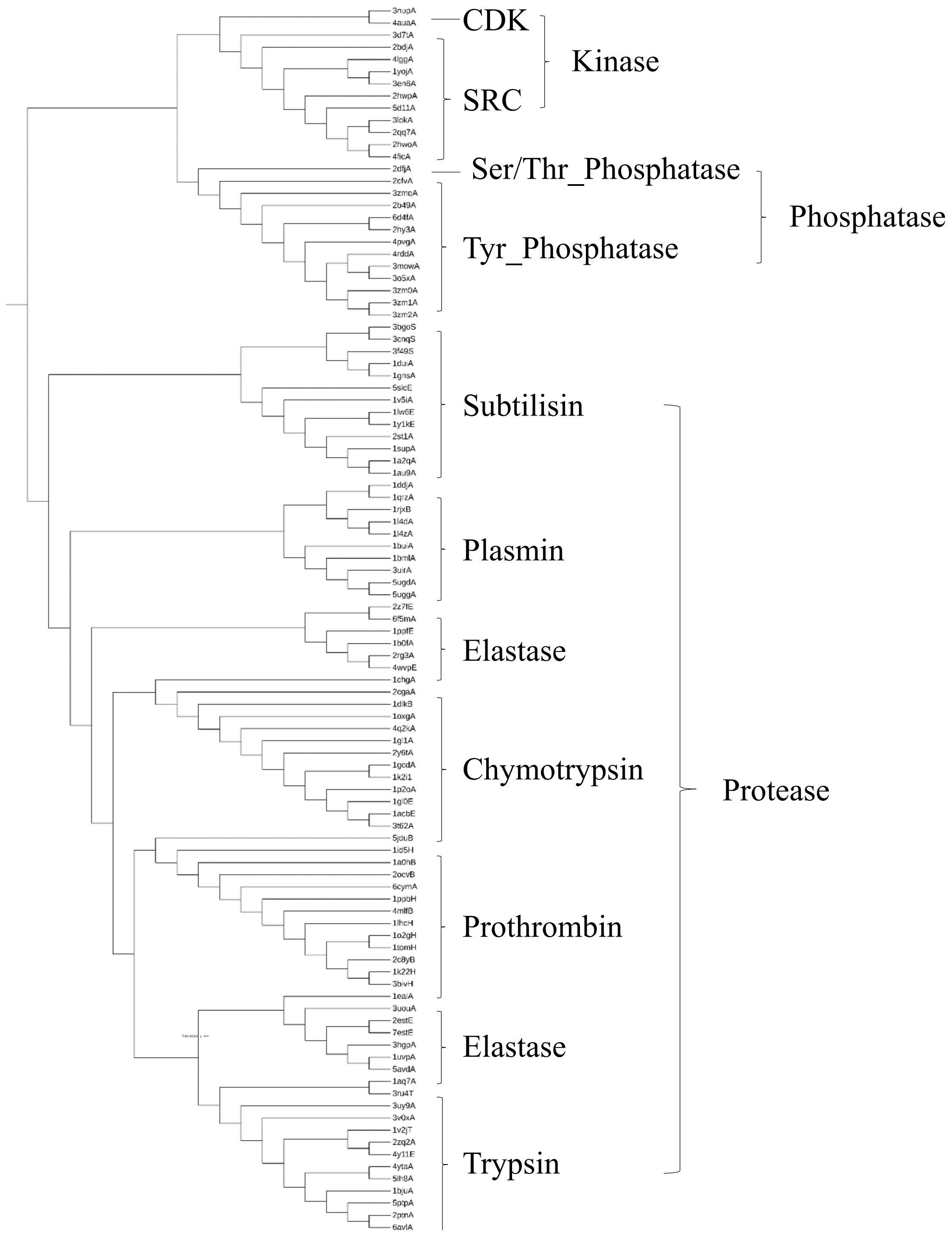

CDK
Kinase
SRC
Ser/Thr_Phosphatase
Phosphatase
Tyr_Phosphatase
Subtilisin
Plasmin
Elastase
Chymotrypsin
Protease
Prothrombin
Elastase
Trypsin

Figure S23

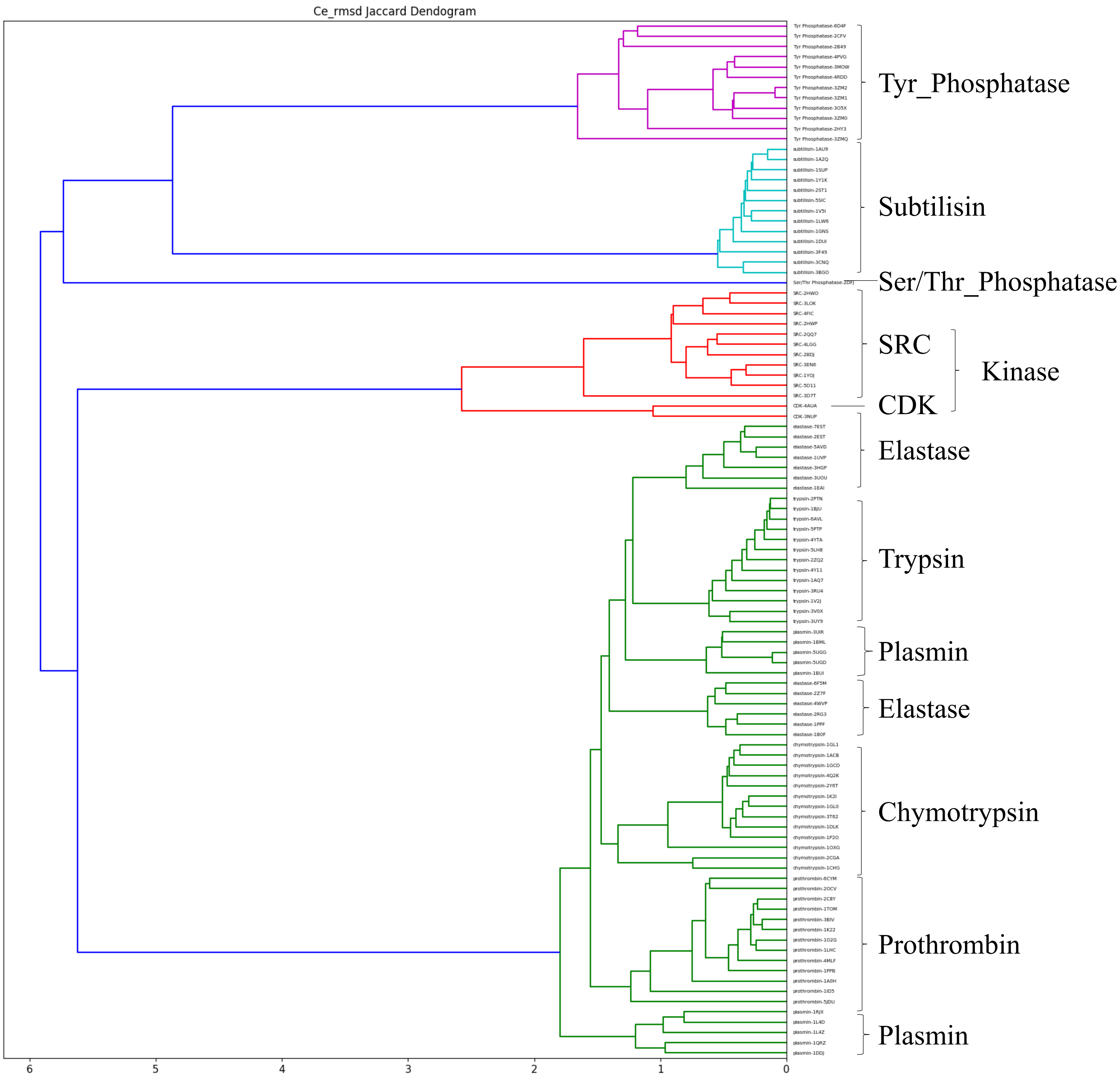
Ce_rmsd Jaccard Dendogram
Tyr Phosphatase-6D4F
Tyr Phosphatase-2CFV
Tyr Phosphatase-2B49
Tyr Phosphatase-4PVG
Tyr Phosphatase-3MOW
Tyr Phosphatase-4RDD
Tyr Phosphatase-3ZM2
Tyr Phosphatase-3ZM1
Tyr Phosphatase-3O5X
Tyr Phosphatase-3ZM0
Tyr Phosphatase-2HY3
Tyr Phosphatase-3ZMQ
subtilisin-1AU9
subtilisin-1A2Q
subtilisin-1SUP
subtilisin-1Y1K
subtilisin-2ST1
subtilisin-5SIC
subtilisin-1V5I
subtilisin-1LW6
subtilisin-1GNS
subtilisin-1DUI
subtilisin-3F49
subtilisin-3CNQ
subtilisin-3BGO
Ser/Thr Phosphatase-2DFJ
SRC-2HWO
SRC-3LOK
SRC-4FIC
SRC-2HWP
SRC-2QQ7
SRC-4LGG
SRC-2BDJ
SRC-3EN6
SRC-1YOJ
SRC-5D11
SRC-3D7T
CDK-4AUA
CDK-3NUP
elastase-7EST
elastase-2EST
elastase-5AVD
elastase-1UVP
elastase-3HGP
elastase-3UOU
elastase-1EAI
trypsin-2PTN
trypsin-1BJU
trypsin-6AVL
trypsin-5PTP
trypsin-4YTA
trypsin-5LH8
trypsin-2ZQ2
trypsin-4Y11
trypsin-1AQ7
trypsin-3RU4
trypsin-1V2J
trypsin-3V0X
trypsin-3UY9
plasmin-3UIR
plasmin-1BML
plasmin-5UGG
plasmin-5UGD
plasmin-1BUI
elastase-6F5M
elastase-2Z7F
elastase-4WVP
elastase-2RG3
elastase-1PPF
elastase-1B0F
chymotrypsin-1GL1
chymotrypsin-1ACB
chymotrypsin-1GCD
chymotrypsin-4Q2K
chymotrypsin-2Y6T
chymotrypsin-1K2I
chymotrypsin-1GL0
chymotrypsin-3T62
chymotrypsin-1DLK
chymotrypsin-1P2O
chymotrypsin-1OXG
chymotrypsin-2CGA
chymotrypsin-1CHG
prothrombin-6CYM
prothrombin-2OCV
prothrombin-2C8Y
prothrombin-1TOM
prothrombin-3BIV
prothrombin-1K22
prothrombin-1O2G
prothrombin-1LHC
prothrombin-4MLF
prothrombin-1PPB
prothrombin-1A0H
prothrombin-1ID5
prothrombin-5JDU
plasmin-1RJX
plasmin-1L4D
plasmin-1L4Z
plasmin-1QRZ
plasmin-1DDJ
6
5
4
3
2
1
0
Tyr_Phosphatase
Subtilisin
Ser/Thr_Phosphatase
SRC
Kinase
CDK
Elastase
Trypsin
Plasmin
Elastase
Chymotrypsin
Prothrombin
Plasmin

Figure S24

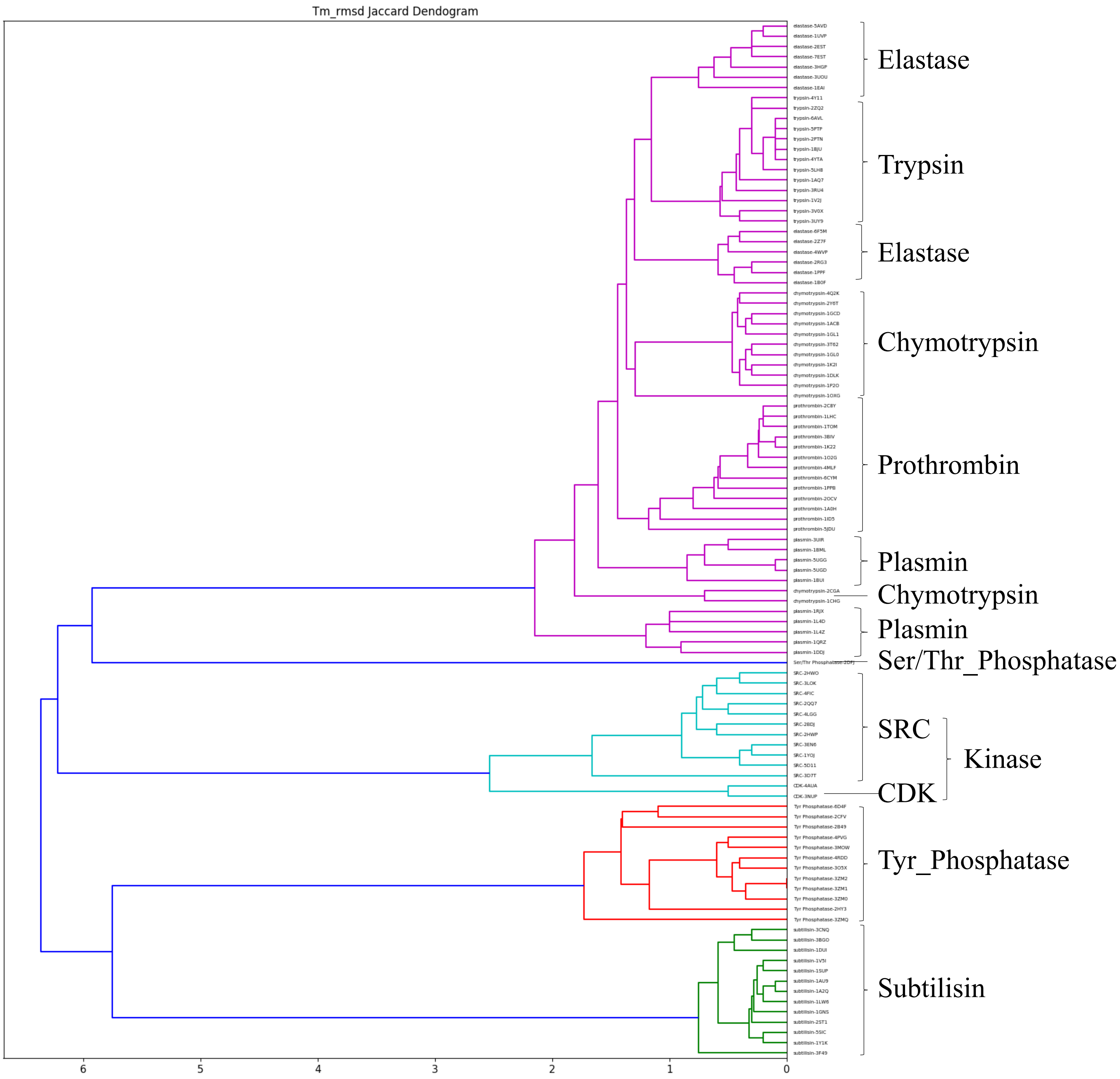
Tm_rmsd Jaccard Dendogram
Elastase
Trypsin
Elastase
Chymotrypsin
Prothrombin
Plasmin
Chymotrypsin
Plasmin
Ser/Thr_Phosphatase
SRC
Kinase
CDK
Tyr_Phosphatase
Subtilisin
6
5
4
3
2
1
0

Figure S25

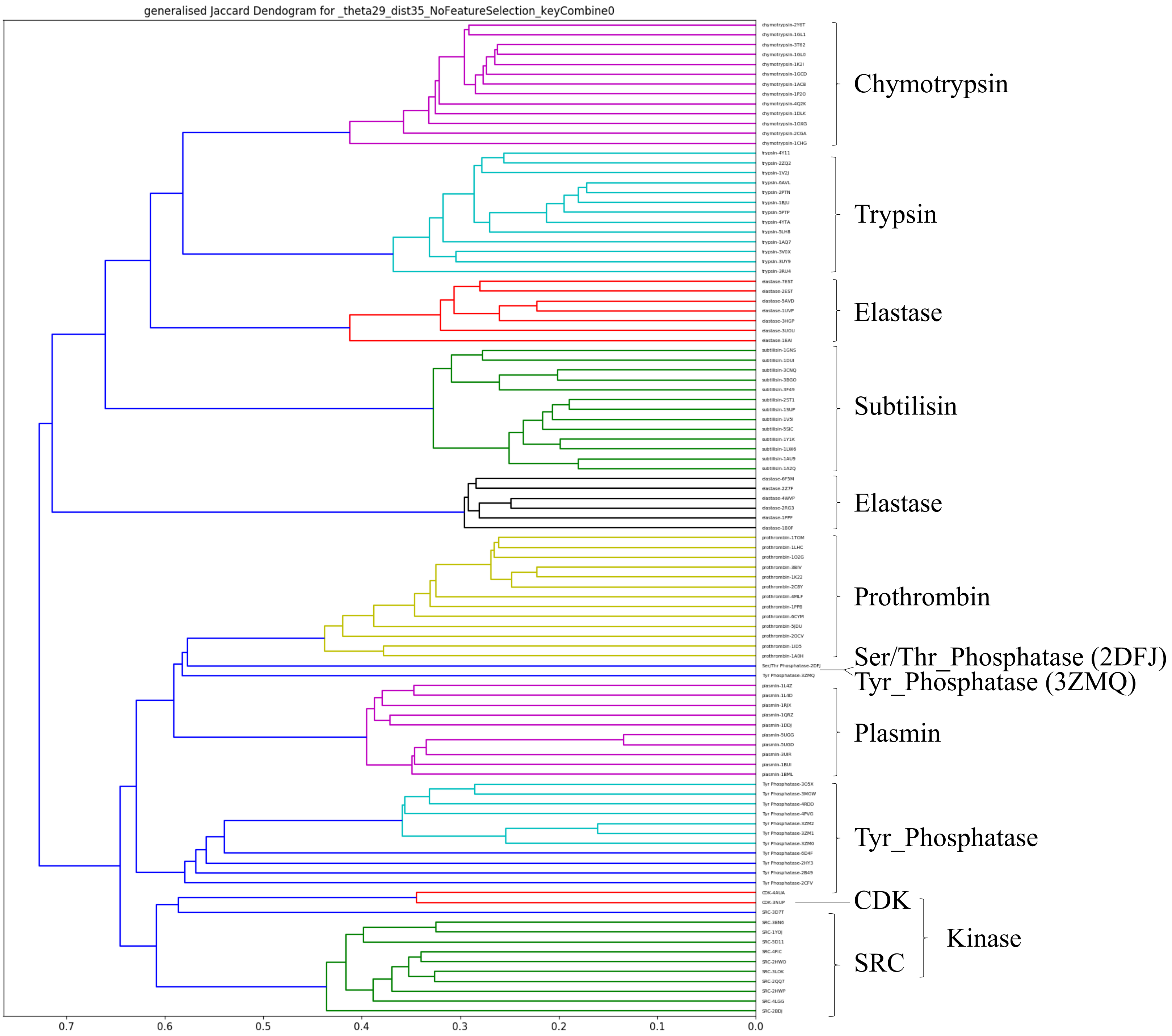

generalised Jaccard Dendogram for _theta29_dist35_NoFeatureSelection_keyCombine0
0.7
0.6
0.5
0.4
0.3
0.2
0.1
0.0
Chymotrypsin
Trypsin
Elastase
Subtilisin
Elastase
Prothrombin
Ser/Thr_Phosphatase (2DFJ)
Tyr_Phosphatase (3ZMQ)
Plasmin
Tyr_Phosphatase
CDK
Kinase
SRC

Figure S26

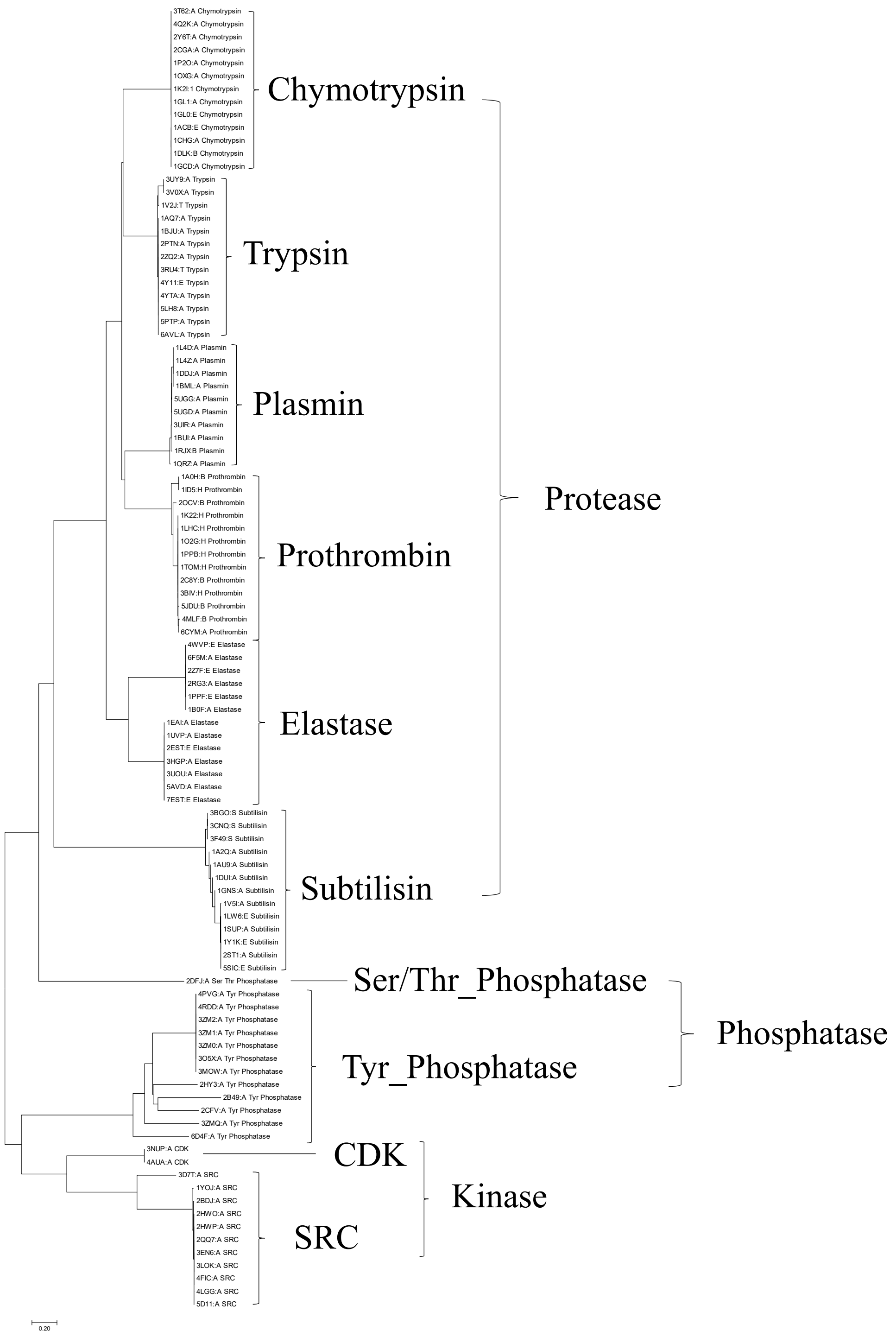
3T62:A Chymotrypsin
4Q2K:A Chymotrypsin
2Y6T:A Chymotrypsin
2CGA:A Chymotrypsin
1P2O:A Chymotrypsin
1OXG:A Chymotrypsin
1K2I:1 Chymotrypsin
1GL1:A Chymotrypsin
1GL0:E Chymotrypsin
1ACB:E Chymotrypsin
1CHG:A Chymotrypsin
1DLK:B Chymotrypsin
1GCD:A Chymotrypsin
Chymotrypsin
3UY9:A Trypsin
3V0X:A Trypsin
1V2J:T Trypsin
1AQ7:A Trypsin
1BJU:A Trypsin
2PTN:A Trypsin
2ZQ2:A Trypsin
3RU4:T Trypsin
4Y11:E Trypsin
4YTA:A Trypsin
5LH8:A Trypsin
5PTP:A Trypsin
6AVL:A Trypsin
Trypsin
1L4D:A Plasmin
1L4Z:A Plasmin
1DDJ:A Plasmin
1BML:A Plasmin
5UGG:A Plasmin
5UGD:A Plasmin
3UIR:A Plasmin
1BUI:A Plasmin
1RJX:B Plasmin
1QRZ:A Plasmin
Plasmin
1A0H:B Prothrombin
1ID5:H Prothrombin
2OCV:B Prothrombin
1K22:H Prothrombin
1LHC:H Prothrombin
1O2G:H Prothrombin
1PPB:H Prothrombin
1TOM:H Prothrombin
2C8Y:B Prothrombin
3BIV:H Prothrombin
5JDU:B Prothrombin
4MLF:B Prothrombin
6CYM:A Prothrombin
Prothrombin
4WVP:E Elastase
6F5M:A Elastase
2Z7F:E Elastase
2RG3:A Elastase
1PPF:E Elastase
1B0F:A Elastase
1EAI:A Elastase
1UVP:A Elastase
2EST:E Elastase
3HGP:A Elastase
3UOU:A Elastase
5AVD:A Elastase
7EST:E Elastase
Elastase
3BGO:S Subtilisin
3CNQ:S Subtilisin
3F49:S Subtilisin
1A2Q:A Subtilisin
1AU9:A Subtilisin
1DUI:A Subtilisin
1GNS:A Subtilisin
1V5I:A Subtilisin
1LW6:E Subtilisin
1SUP:A Subtilisin
1Y1K:E Subtilisin
2ST1:A Subtilisin
5SIC:E Subtilisin
Subtilisin
Protease
2DFJ:A Ser Thr Phosphatase
Ser/Thr_Phosphatase
4PVG:A Tyr Phosphatase
4RDD:A Tyr Phosphatase
3ZM2:A Tyr Phosphatase
3ZM1:A Tyr Phosphatase
3ZM0:A Tyr Phosphatase
3O5X:A Tyr Phosphatase
3MOW:A Tyr Phosphatase
2HY3:A Tyr Phosphatase
2B49:A Tyr Phosphatase
2CFV:A Tyr Phosphatase
3ZMQ:A Tyr Phosphatase
6D4F:A Tyr Phosphatase
Tyr_Phosphatase
Phosphatase
3NUP:A CDK
4AUA:A CDK
CDK
3D7T:A SRC
1YOJ:A SRC
2BDJ:A SRC
2HWO:A SRC
2HWP:A SRC
2QQ7:A SRC
3EN6:A SRC
3LOK:A SRC
4FIC:A SRC
4LGG:A SRC
5D11:A SRC
SRC
Kinase
0.20

Figure S27

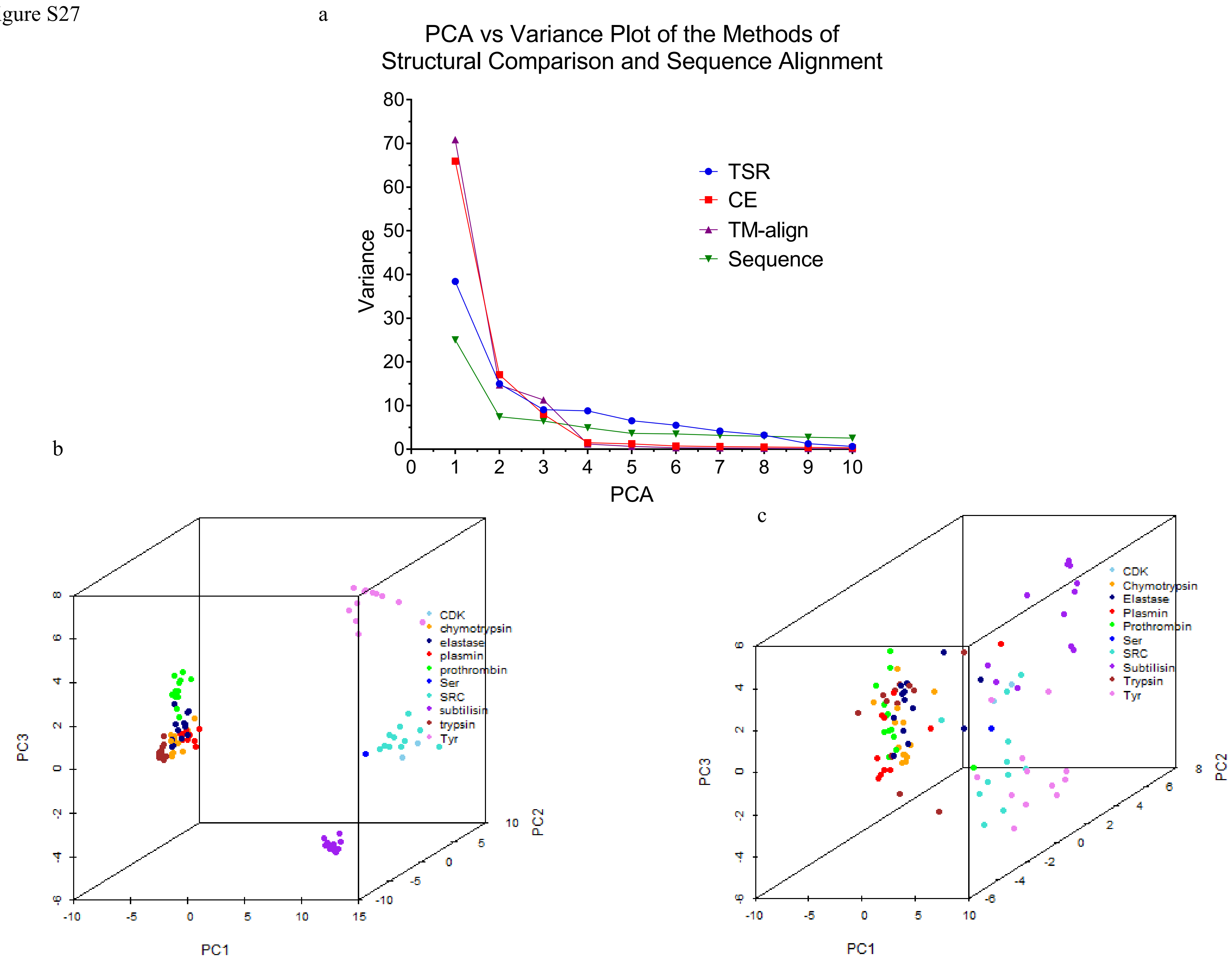

a
PCA vs Variance Plot of the Methods of
Structural Comparison and Sequence Alignment
Variance
PCA
TSR
CE
TM-align
Sequence
b
PC1
PC2
PC3
CDK
chymotrypsin
elastase
plasmin
prothrombin
Ser
SRC
subtilisin
trypsin
Tyr
c
PC1
PC2
PC3
CDK
Chymotrypsin
Elastase
Plasmin
Prothrombin
Ser
SRC
Subtilisin
Trypsin
Tyr

Figure S28

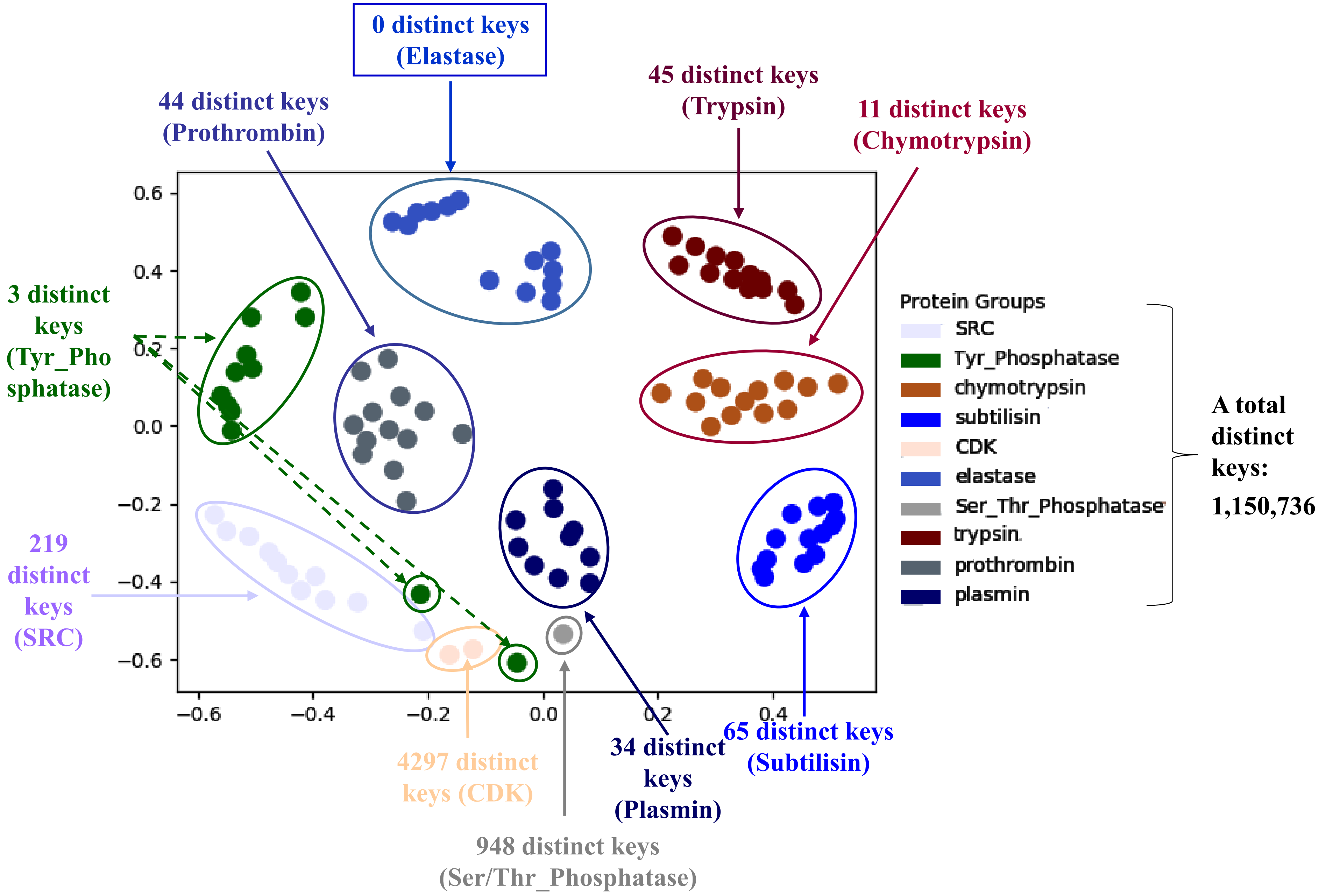
0 distinct keys (Elastase)
44 distinct keys (Prothrombin)
45 distinct keys (Trypsin)
11 distinct keys (Chymotrypsin)
3 distinct keys (Tyr_Phosphatase)
219 distinct keys (SRC)
4297 distinct keys (CDK)
948 distinct keys (Ser/Thr_Phosphatase)
34 distinct keys (Plasmin)
65 distinct keys (Subtilisin)
0.6
0.4
0.2
0.0
−0.2
−0.4
−0.6
−0.6
−0.4
−0.2
0.0
0.2
0.4
Protein Groups
SRC
Tyr_Phosphatase
chymotrypsin
subtilisin
CDK
elastase
Ser_Thr_Phosphatase
trypsin
prothrombin
plasmin
A total distinct keys: 1,150,736

Figure S29

a

Theta 29 and MaxDist 35: ARI: 1.00

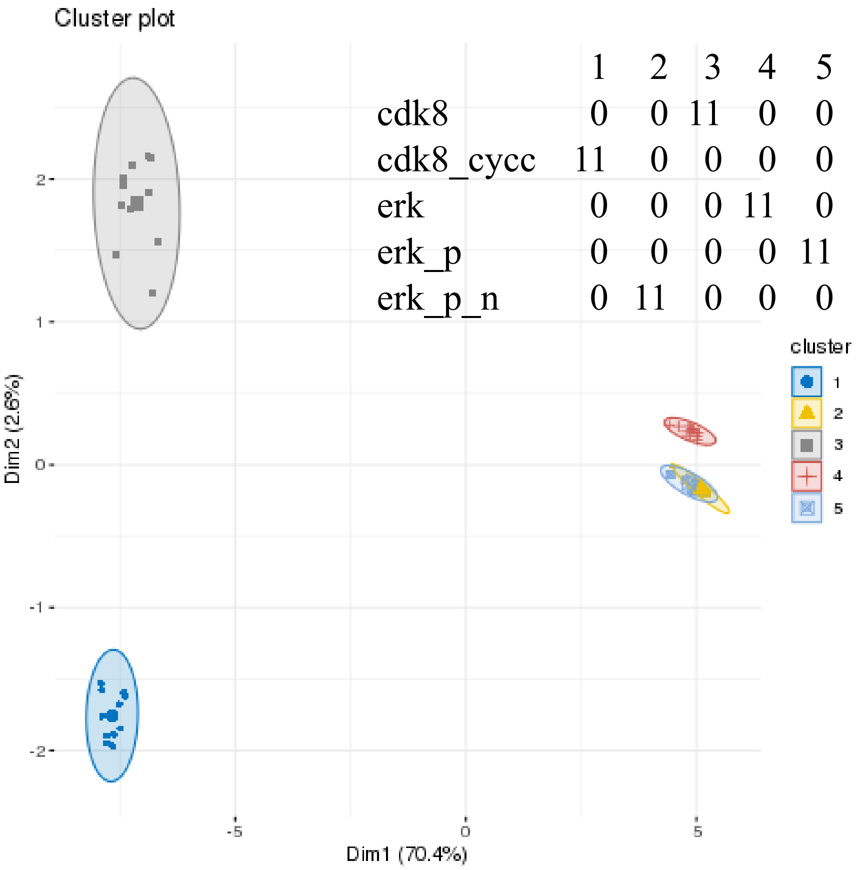

b

Theta 7 and MaxDist 12: ARI: 0.953

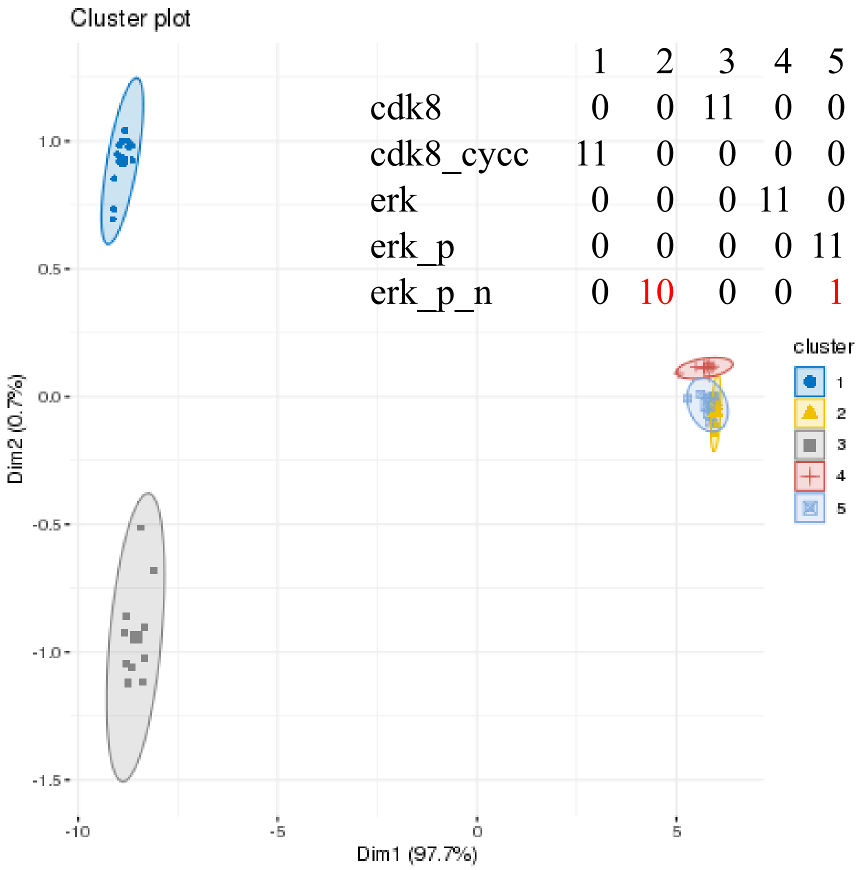

Supplement: Supplementary file 16 [file Data_Sheet_1.PDF]
